# Effect of Village Health Team Home Visits and Mobile Phone Consultations on Maternal and Newborn Care Practices in Masindi and Kiryandongo, Uganda: A Community-Intervention Trial

**DOI:** 10.1371/journal.pone.0153051

**Published:** 2016-04-21

**Authors:** Richard Mangwi Ayiasi, Patrick Kolsteren, Vincent Batwala, Bart Criel, Christopher Garimoi Orach

**Affiliations:** 1 Makerere University, School of Public Health, College of Health Sciences, P.O Box 7072, Kampala, Uganda; 2 Mbarara University of Science and Technology, P.O Box 1410, Mbarara, Uganda; 3 Institute of Tropical Medicine, Nationalestraat 150, B 2000 Antwerp, Belgium; Karolinska Institutet, SWEDEN

## Abstract

**Introduction:**

The World Health Organisation recommends home visits conducted by Community Health Workers (in Uganda known as Village Health Teams—VHTs) in order to improve maternal and newborn health. This study measured the effect of home visits combined with mobile phone consultations on maternal and newborn care practices.

**Method:**

In a community intervention trial design 16 health centres in Masindi and Kiryandongo districts, Uganda were randomly and equally allocated to one of two arms: control and intervention arms. Eight control health centres received the usual maternal and newborn educational messages offered by professional health workers and eight intervention health centres that received an intervention package for *maternal care* and *essential newborn care* practices. In the intervention arm VHTs made two prenatal and one postnatal home visit to households. VHTs were provided with mobile phones to enable them make regular telephone consultations with health workers at the health centre serving the catchment area. The primary outcome was health facility delivery. Other outcomes included antenatal attendances, birth preparedness, cord and thermal care and breastfeeding practices. Analysis was by intention-to-treat.

**Results:**

A total of 1385 pregnant women were analysed: 758 and 627 in the control and intervention arms respectively. Significant post-intervention differences were: delivery place [adjusted Odds Ratio aOR: 17.94(95%CI: 6.26–51.37); *p*<0.001], cord care [aOR: 3.05(95%CI: 1.81–5.12); *p*<0.001] thermal care [aOR: 7.58(95%CI: 2.52–22.82); *p*<0.001], and timely care-seeking for newborn illness [aOR: 4.93(95%CI: 1.59–15.31); *p* = 0.006].

**Conclusion:**

VHTs can have an effect in promoting proper cord and thermal care for the newborn and improve timely care-seeking for health facility delivery and newborn illness, because they could answer questions and refer patients correctly. However, VHTs should be supported by professional health workers through the use of mobile phones.

**Trial Registration:**

ClinicalTrials.gov NCT02084680

## Introduction

Achieving targets for MDGs 4 and 5 in developing countries has proved elusive. Each year 298,000 women die due to pregnancy related causes, two thirds being in Africa alone [1], while 2.8 million babies die within the first month of life [2]. In Uganda, maternal and neonatal mortality are estimated to be 438/100,000 and 27/1000 live births, respectively [2,3]. Two thirds of these deaths could be averted by interventions known to be cheap and effective [4,5], namely: creativity in health education, behaviour change communication [6,7] and healthy behaviours practiced at home and in the communities [8].

Access to relevant information during prenatal and immediate postnatal period is critical. The World Health Organisation (WHO) and UNICEF recommends home visits conducted by Community Health Workers (CHWs, also known as Village Health Teams in Uganda-VHTs) as a means to communicate relevant health messages for maternal and newborn health [9]. Evidence from Bangladesh, India and Pakistan [10,11], Sub Saharan Africa [12–14] including Uganda [15] reinforce this recommendation.

CHWs’ programmes are designed to bridge the gap between communities and formal health systems, but the problem with most Community Health programmes is that they operate far away from formal health systems. Moreover, CHWs are lay people with limited health knowledge. On the other hand, there is lack of appropriate programmes at the community and health facility level to address the problem of maternal and newborn care practices in Uganda [16,17].

In this study, VHTs equipped with mobile phones conducted home visits to offer maternal and newborn health education messages at household level. The objective of this study was to measure the effect of combining home visits made by VHTs with mobile phone consultations on maternal and newborn care practices. Outcome measures were maternal care practices (antenatal care attendance, birth preparation, skilled attendance at birth) and newborn care practices (hygienic cord care, thermal care, initiation of exclusive breastfeeding within one hour and care-seeking for newborn illness). Results from this study will advance the knowledge on how interactions between VHTs and professional health workers can be exploited to improve maternal and newborn care practices at the household level.

## Methodology

### Study area and population

The study was conducted in Masindi and Kiryandongo districts of Uganda from May/June 2013 to October/December 2014. At the time of conceptualising this study in 2010/2011 these two districts were one administrative unit known as Masindi district, later split to form Kiryandongo and Masindi districts as separate administrative entities. Otherwise, the ethnicity, socio-demographic characteristics and access to health care in the two districts are comparable. The combined population for the two districts is estimated at 700,000 [18]. The district health system in Uganda is structured into four-tiers. Health centre (HC) level II, III, IV and general hospital. HC II is based in a parish with an estimated service population of 20,000 inhabitants and headed by a general nurse. HC level III is located at a sub county with estimated population of 50,000 inhabitants and headed by a clinical officer. HC level IV is located in a county (average population of 150,000 inhabitants) and headed by a senior medical officer while a general hospital is located at a district (estimated population of 300,000 inhabitants) and headed by a principal medical officer. HCs of levels II and III provide mainly ambulatory care while HC IV and hospital provide inpatient services and emergency surgery. The HCs relevant for our study were levels II and III. There are 44 HCs of level II and III in this region (26 in Masindi and 18 in Kiryandongo). Eighty per cent of the population lives within a five-kilometre of the nearest Health facility. About 97% of pregnant women make at least one antenatal care consultation, but less than 50% deliver in a health facility [19].

All villages have VHTs who have been trained on community mobilisation. The guidelines for selection of VHTs and their mandate were published elsewhere [20,21]. In Uganda VHT members are selected from among members of the communities by community members themselves. VHTs could be male or female adults and their levels of education ranges from 7–11 years of schooling whereby they can read and comprehend basic communications in the English language. District health systems train VHTs to capacitate them to provide health education and mobilise communities for preventive services such as vaccination campaigns, safe water programmes and medicines distribution for deworming and treatment for malaria, diarrhoea and pneumonia under the iCCM (Integrated Community Case Management for Malaria, Pneumonia and Diarrhoea) programme.

All villages in Masindi and Kiryandongo have a fair coverage of mobile telecommunication network. Regional data suggests that up to 80% of the population can access mobile telephone networks [22].

### Study design and randomisation

This was a randomised community intervention trial. We used a pragmatic approach whereby the intervention was integrated within the routine healthcare delivery system as opposed to highly controlled experimental design [23–25]. However, standard operating procedures were developed for health workers and VHTs (see S1 File). Random allocation was at three levels-HCs, Villages then VHTs. Blinding was not necessary since randomization was at the level of HCs. HCs that offered antenatal and delivery care were eligible for randomisation. Random allocation was conducted by writing the names of HCs, VHTs or villages on small pieces of papers which were then folded to conceal the names. Two persons, each representing a study arm, but not associated to the study were asked to randomly pick folded papers from the pool of names. The process was repeated until allocation was completed for HCs, villages and VHTs. A proportionate number of 16 public HCs were selected from Masindi (10 HCs out of 26) and Kiryandongo (six HCs out of 18) then randomly assigned to control (eight HCs) or intervention (eight HCs) arm. Ten villages were randomly selected from the catchment areas of each HC. Normally, three VHTs per village are selected and trained. However, for purposes of this study, one VHT was assigned to work in two villages since the number of women to be recruited per village was estimated to be very low at 10 women per village over a period of 6–12 months. Therefore, five VHTs were randomly selected from the ten villages. Eight VHTs, two each for four of the intervention HCs, were added when it was realised that their catchment areas had wider geographical coverage, or selected villages were found to be far apart. For example, one catchment area was identified to be across a river bank that necessitated crossing by means of a ferry. Selected VHTs were given a monthly reimbursement of 10,000 Uganda shillings (equivalent to 5 USD in 2013) for their transport as suggested by the VHT policy guideline [21].

### Sample size estimation

We used the formula for cluster randomised trials suggested by Hayes and Bennett [26] to estimate the sample size of 1,388 participants from 16 clusters. This was based on 80% power to detect a 10% difference between two comparison groups with a design effect of 2.0 and Coefficient of variation between clusters, km, 0.35 considering 10% loss to follow up. The current prevalence of institutional deliveries was considered to be 23.5% [27].

### Selection of study participants and procedure

Pregnant women originating from selected villages were consecutively recruited during their first antenatal visits. Based on pre-intervention visit observations, the required sample size could be realised within seven months of initiation of the study if 80% of the women making first antenatal visits were recruited. The cut-off point of 28 weeks of gestation was assessed as suitable to achieve this end. Age of pregnancy was determined by palpation. During selection, the purpose of the study was explained and a written consent secured. Biodata including village, name of the spouse, previous and current obstetric histories were also recorded. No decline to participate were registered. Eligible pregnant women who were approached readily consented to participate in the study, partly in anticipation of material benefits. In this study we did not offer financial or material benefits to pregnant women. Consented women were issued with a counter-referral form to be delivered to the responsible VHT. The health worker further made a telephone call to notify the VHT about the newly consented woman. After receiving the phone call, VHTs located the pregnant woman and her family to make appointment for the first home visit. Three home visits were planned: The first one was conducted soon after enrolment and the second made four weeks later. The third visit was scheduled within three days after delivery of the baby. We expected the last woman recruited to deliver by August 2014 (15^th^ month of the study), however, follow-up was extended to November 2014, by three extra months.

### Intervention package

In addition to routine educational messages offered in ANC clinics, the intervention arm received a package of two closely linked components: **i**) VHTs making home visits to provide educational messages for maternal and newborn care **ii**) each VHT was equipped with a mobile phone handset capable of making unlimited phone call consultation with professional health workers in case of further clarification or advice. Health workers involved in the study were also provided with mobile phone handsets. Voice communication was preferred over short messages systems because the former had the advantage of interactive consultation between VHTs and professional health workers. No special arrangements were made for evacuating women from the health centre to hospital. However, women from both intervention and control groups could access ambulance services through the routine means of evacuation to the hospital.

### Training of VHTs

Forty-eight VHTs were selected (29 from Masindi and 19 from Kiryandongo) for training. Two VHT training sessions in Masindi and one in Kiryandongo were conducted consecutively each lasting five days. Training sessions had between 14–17 participants each. Smaller groups were preferred for the training to facilitate better learning. Training was based on the VHT handbook locally designed by the Ministry of Health in Uganda as a job aid for VHTs [28]. The handbook elaborates promotional information for pregnant women and essential newborn care practices. To maintain enthusiasm among trainees each training-day lasted between 9.00 am to 2.00 pm. The first day was dedicated to the problem of newborn care and its importance. On Day two, VHTs were taught how to conduct an educational session with the families. On Day three and Day four role-plays were conducted and pre-test in a real life situation using a field-based practicum was conducted. The practicum sessions were critiqued by fellow VHTs and improvements suggested. During the fifth day, VHTs were initiated into the actual study protocol with the aid of a standard operating procedure. Two professional health workers (one midwife and a nurse) from each of the intervention health centres were included in the training.

### Scope of home visit education

Home visit sessions adopted a group-discussion approach whereby VHTs became facilitators of a discussion rather than the provider of knowledge per se [16]. In the first home visit VHTs held discussions on two topics: *general care* (importance of: Intermittent Presumptive Treatment for malaria (IPT), regular folic acid supplements, consistent use of bed nets, maternal diet, antenatal consultations and institutionalized delivery); *danger signs in pregnancy* (Vaginal bleeding, Convulsions, fever, Water loss [drainage of amniotic fluid], abdominal pains, severe headaches, blurred vision, swelling of limbs, absent or diminished foetal movement). The second visit required VHTs to discuss about *birth preparation* (Identifying place of delivery, identify a skilled birth attendant, organizing transport, setting aside some money, Planning for emergency evacuation, Planning with a family member) and the *items needed for delivery* (Clean plastic sheet for delivery, Clean dry towel for mother and baby, New razorblade, Clean threads, Pairs of gloves). VHTs explained how to care for the newborn baby by hygienic cord care, initiation of breast feeding within the first hour after birth, avoiding pre-lacteal feeds and delayed bathing for at least three days. These topics were adapted from the WHO recommendations for home visits [9] and based on formative studies [16,19]. During home visits, VHTs could make mobile phone consultations with professional health workers on issues that they considered challenging [20]. The home visit session was concluded with a recap by the VHT highlighting important areas for the family’s attention. Each session lasted 60–90 minutes.

### Control group

In the control group the same recruitment process as in the intervention arm was applied. Participants’ bio-data were recorded in personal files. The major difference between the two groups was that the control group did not receive follow-up visits by VHTs and VHTs in the control group were not provided with mobile phones. However, pregnant women in the control group continued to receive group education routinely offered in the health centres. Details of how antenatal care is provided in routine care has been described elsewhere [16]. Women in the control group were next visited in their homes during quantitative data collection.

### Data collection

Data collection was limited to two periods February-March and October-December 2014 in order to maximise efficiencies of resources. Also, these are the seasons when the pastoralist study population is settled in one place. During each session of data collection ten research assistants and three supervisors were engaged. Supervisors ensured appropriate deployment of research assistants and checked to ensure all questionnaires were correctly filled. Prior to data collection, lists of recruited women were obtained from HCs and provided to the VHTs for guidance to women’s homes. Where the VHT was not sure especially in the control arm, local councils were asked for direction. At the household, a verbal consent was obtained to permit second data collection. All women approached consented to the second interview. Structured questionnaires were used to collect data on maternal practices such as antenatal attendance, birth preparation and place of delivery; newborn care practices such as tying and cutting of the cord, wrapping and bathing of the newborn, initiation of exclusive breastfeeding and care-seeking in case of newborn illness. The longest interval between delivery of the woman and data collection was 4–6 months. Women who were not found at home on first visit were followed up on the second day and two weeks later. Women were declared lost to follow-up if not found on third attempt.

### Primary outcomes

Health facility delivery was the primary outcome for this study. Pregnant women who delivered at home, with TBAs or along the way were grouped as *home delivery* and those who reported HC or hospital delivery were grouped as *health facility delivery* = 1. Other maternal outcomes of interest comprised of antenatal consultations and birth preparation, while newborn care practices comprised of cord care, thermal care, initiation of exclusive breastfeeding and care-seeking for newborn illness. All variables were dichotomised by building composite variables from discrete variables. Women who reported having three or more antenatal consultations were categorised as adequate and the rest were grouped as inadequate. Women were considered to be *adequately prepared* = 1 if they secured warm clothing for the baby, saved some money, decided on place of delivery, identified a caretaker and a means of transport to the health facility in case of labour. Care for the cord was considered to be *clean* = 1 if the mother reported using new strings for tying the cord, sterile instrument or new razorblade for cutting and did not apply any substance on the cord. Regarding thermal care, women were categorised as *appropriate* = 1 if they reported drying the baby before or immediately after expulsion of the placenta, wrapping the baby before or soon after expulsion of placenta and delayed bathing for 24 hours or more. Breastfeeding was considered *appropriate = 1*, if the mother reported initiating breastfeeding within six hours of delivery and did not give any pre-lacteal feeds. Care-seeking for newborn illness was considered to be *timely* when a mother reported seeking for care within 24 hours on recognition of symptoms of newborn illness. A newborn was considered to have *complete* = 1 vaccination status if they had received both Polio 0 and BCG vaccines at the time of data collection.

### Data analysis

Data were entered in EpiData version 3.1 ("The EpiData Association" Odense, Denmark) and exported to STATA version 12 (StataCorp, College Station, Texas 77845 USA) for analysis. Analysis was by intention-to-treat whereby participants were kept in the groups in which they were randomised and final analysis excluded those with ‘*missing of data*’ about outcomes of interest (lost to follow up, had abortions) [29]. The dataset was declared a survey data by using *svy*-commands in STATA. Pre-intervention household and individual characteristics were compared between control and intervention arms and their differences estimated using the chi-square test statistic. Pre-intervention differences were considered to have occurred by chance, however covariates that showed significant differences were included in multi-variable analysis. Since individuals within a cluster are likely to be correlated we used cluster level analysis to cater for intra-cluster correlation. Odds Ratios were calculated using random effects, employing the *xtmelogit* command in STATA because our outcomes were of binary nature.

### Multilevel analysis

Unadjusted models were fitted with the primary outcome and treatment arms only. Covariates that showed statistical significance (*p*-value <0.05) were fitted into the backward regression model. The generic STATA command was:

xtmelogit [primary outcome] [trial arm] X1 X2 X3…|| hc level:, covariance (unstructured) OR where, **X** = explanatory variables**; hc level** = Health Centre level; **OR** = Odds Ratio

Only covariates that showed *p*-values equal ≤0.05 were left, then the Odds Ratio, confidence interval and *p*-value of the treatment arm were reported as adjusted values.

### Ethical considerations

The study was approved by the Higher Degrees and Ethics Committee of the School of Public Health, Makerere University in March 2012 and in March 2014 registered with clinicalTrials.gov: NCT02084680. This registration was done when recruitment of study participants had already started because the first author (RMA) became aware about the need for registration after reviewing other trials studies in the literature. Participants provided written consent at the time of enrolment and verbal consent during post-intervention evaluation thereafter. Personal files for recruited women were kept in locked cupboards which were accessible only to the research team members. The dataset for this study is also available as: S2 File.

### Quality control

In addition to role-plays conducted during the training, three researchers with social science background periodically participated in some of the home visit sessions to observe how VHTs steered the discussions. In order to minimize the effects of an observer whereby women may be shy to participate [30], researchers were allowed to also participate in the discussions. Pregnant women were randomly selected and interviewed to ensure that the sessions were conducted as required. Where disparities were noticed, refresher trainings were organized for all VHTs and the health workers. Consequently, two refresher trainings were conducted for both VHTs and health workers in the months of October 2013 and January 2014 by the first author assisted by the two district trainers. The two-days refresher trainings focused on key maternal and newborn care practises. The same training group as the initial training were used.

### Limitations to the intervention

Health worker attrition was an important setback because it delayed the recruitment of study participants. For example, two health workers got six months’ study leave, three health workers were transferred to non-participating health centres within the district, and one got employment in another district local government. Some female staff got 60 days’ maternity leave and combined with 30 days’ annual leave making 90 working days out of duty station. Again midwives frequently attended workshops causing further delays in the recruitment process. Interestingly, we did not register attrition among VHTs among VHTs during the study period [20].

## Results

### Participant characteristics

**Fig 1:**
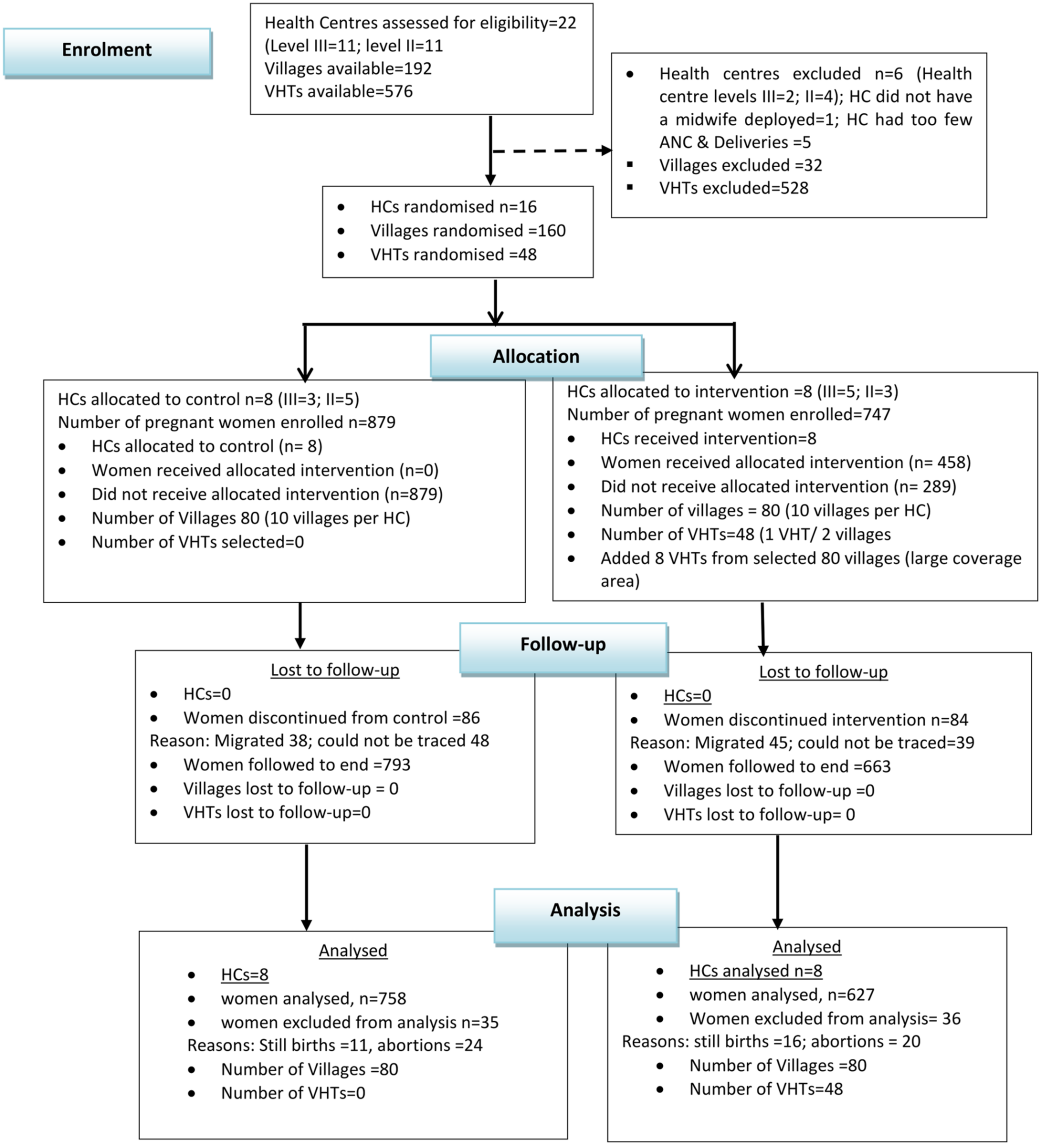
Trial Profile using the CONSORT Flow Diagram.

Pre-intervention characteristics in the treatment arms were comparable for religion, level of education, outcome of previous pregnancy (live birth or abortions) and gestation age at recruitment. However, there were significant differences in individual and household characteristics such as age of the mother, ethnicity, and sources of income, parity and number of antenatal consultations in previous pregnancy (Table 1). In total 1,644 pregnant women were enrolled (893 control and 751 intervention). In the intervention arm about two-thirds (64%) of the women reported at least one home visit by a VHT. About ten per cent of women in each arm 87/879 (control) and 84/751 (intervention) were declared ‘lost to follow-up’ because they could not be traced after the intervention while; 35/879 and 36/751 among control and intervention arms respectively were declared ‘missing of data’ [29] because they did not have the outcome of interest. The final analysis centred on 1385 with 758 in control and 627 in intervention arms. (See Fig 1).

**Table 1:** Baseline characteristics of control and intervention arms.

Variable	Control	Intervention	*p*-value
	n(%)	n(%)	
**Household characteristics n = 1,385**			
**Age of woman**			
13–24 yrs.	398 (52.5)	377 (60.1)	
≥25 yrs.	360 (47.5)	250 (39.9)	0.04
**Ethnicity**			
Migrant	593 (78.2)	424 (67.6)	
Indigenous	165 (21.8)	203 (32.4)	<0.001
**Religion**			
Other religion	59 (7.8)	37 (5.9)	
Christian	699 (92.2)	590 (94.1)	0.17
**Education**			
None/primary	635 (83.8)	525 (83.7)	
Secondary/tertiary	123 (16.2)	102 (16.3)	0.98
**Marital status**			
Single/separated	70 (9.2)	88 (14.0)	
Living with spouse	688 (90.8)	539 (86.0)	0.005
**Source of income**			
None/housewife	365 (48.2)	359 (57.3)	
Regular/stable income	393 (51.8)	268 (42.7)	0.001
**Selected Obstetric characteristics**			
**Number of pregnancy**			
Second/more	629 (83.0)	456 (72.7)	
First	129 (17.0)	171 (27.3)	<0.001
**Number of ANC visits in previous pregnancy (n = 1,085)** *			
0–3 visits	238 (37.8)	231 (50.7)	
4/more visits	391 (62.2)	225 (49.3)	<0.001
**Outcome of last pregnancy**			
Abortion/stillbirth	44 (7.0)	27 (5.9)	
Term/live baby	585 (93.0)	429 (94.1)	0.48
**Where last delivery took place (n = 1,046)** *			
Home/way to facility	314 (52.1)	204 (46.1)	
Health facility	289 (47.9)	239 (53.9)	0.05
**Attendant at birth**			
Non-professional	301 (49.9)	184 (41.5)	
Professional health worker	302 (50.1)	259 (58.5)	0.007
**Gestation age at recruitment**			
> 20 weeks	381 (50.3)	346 (55.2)	
≤ 20 weeks	377 (49.7)	281 (44.8)	0.07
**Level of health Centre**			
Level II	514 (67.8)	198 (31.6)	
Level III	244 (32.2)	429 (68.4)	<0.001

*Excludes women that did not carry pregnancy to term

### Delivery of the intervention

A total of 355 mobile phone consultations were recorded. Two thirds, 239/355 (67.3%) were related to the mother and the rest were related to the newborn. The most common prenatal mobile phone consultations were concerned with onset of labour (26.8%) while newborn illnesses such as fever, pneumonia and convulsions (33.6%) were the most common condition for the newborn [See Table 2].

**Table 2:** Mobile phone consultations made by VHTs to professional health workers.

Condition	Frequency	Percent
**Conditions related to pregnant women (239)**		
Labour related	64	26.8
Others (diet, physical activities, body weakness)	61	25.5
Abdominal & Back Pain	40	16.7
Fever, Cough & Flu	35	14.6
Vaginal Bleeding	21	8.8
Drainage of Liquor	18	7.5
**Conditions related to newborn baby (116)**		
Fever, Cough, Pneumonia & Convulsions	39	33.6
Thermal care	26	22.4
Breastfeeding	23	19.8
Cord	16	13.8
Others (excessive crying, sleep, emptying of bowels)	12	10.3

### Rates of prenatal and newborn care practices

Attendance of four antenatal care visits was similar in both arms (39% among control and 37% among intervention). Regarding birth preparations, about four out of five secured warm clothe among both arms. But a higher proportion among intervention compared with control arm had secured some money (58% vs 36%), and planned for place of delivery (43% vs. 11%). Health facility delivery was three times higher among intervention (87%) compared with control arm (28%) (see Table 3).

**Table 3:** Post-intervention prenatal and newborn care practices.

Variables	Control n(%)	Intervention n(%)
**Prenatal care practices**		
**Number of prenatal care visits to health centre**		
One	70(9.2)	56(8.9)
Two	148(19.5)	36(5.7)
Three	147(19.4)	155(24.7)
Four	293(38.7)	231(36.8)
More than four	100(13.2)	149(23.8)
**Birth preparation (multiple response)**		
Secured warm clothing	604 (79.7)	553 (88.2)
Saved some money	273 (36.0)	362 (57.7)
Decided on delivery place	80 (10.6)	271 (43.2)
Identified a caretaker	115 (15.2)	209 (33.3)
Identified a means of transport	116 (15.3)	287 (45.8)
**Place of delivery**		
Health centre	142 (19.0)	433(71.8)
Hospital	58(7.8)	92(15.3)
Home	360(48.3)	65(10.8)
On way	43(5.8)	5(0.8)
Traditional Birth Attendant	137(18.4)	4(0.7)
Nursing home	6(0.8)	4(0.7)
**Newborn care practices**		
**Material for tying the cord**		
Clean thread	514(69.4)	531(88.4)
Material from old cloth	142(19.2)	49(8.2)
Rages	85(11.5)	21(3.5)
**Instrument for cutting cord**		
New razorblade	408(55.1)	300(49.9)
Sterile instrument	305(41.2)	284(47.3)
Used razorblade	16(2.2)	1(0.2)
Sharp objects in household	12(1.6)	16(2.7)
**Application of substance on the cord (multiple response)**		
Baby powder	279(36.8)	156(24.9)
Animal wastes	6(0.8)	1(0.2)
Soot powder	116(15.7)	48(8.0)
Herbal medicines	44(5.9)	22(3.7)
**Time of drying newborn**		
Before placenta was delivered	438(59.1)	380(63.2)
Soon after placenta delivered	216(29.2)	148(24.6)
After entire delivery process	25(3.4)	21(3.4)
Not dried just wrapped	62(8.4)	52(8.7)
**Time of wrapping newborn**		
Before placenta delivered	497(67.1)	394(65.6)
Soon after placenta delivered	239(32.3)	198(33.0)
Long after delivery	5(0.7)	9(1.5)
**Timing of first bath after delivery**		
Immediate	101(13.6)	14(2.3)
≤ 6hrs	230(31.0)	36(6.0)
7-23hrs	252(34.0)	77(12.8)
Second day	132(17.8)	189(31.5)
Third day	20(2.7)	206(34.3)
After third day	6(0.8)	79(13.1)
**Timing of initiation of breastfeeding**		
Within 1 hrs.	347(46.8)	331(55.1)
1–6 hrs.	234(31.6)	152(25.3)
7-23hrs.	100(13.5)	73(12.2)
≥24hrs.	60(8.1)	45(7.5)
**Offer of Pre-lacteal feeds**		
Water only	20(2.6)	24(3.8)
Water + sugar	46(6.1)	40(6.4)
Water + glucose	98(12.9)	75(12.0)
Water + tea	21(2.8)	13(2.1)
**Newborn vaccination status**		
Polio 0	376(51.1)	389(65.5)
BCG	711(96.6)	565(96.1)
**Newborn illness**		
Yes	192(25.9)	156(26.3)
**Time for care seeking**		
Within 1hr.	16(8.3)	31(19.9)
Within2- 6hrs	34(17.7)	50(32.1)
Within 7–24 hrs.	70(36.5)	54(34.6)
>24hrs later	72(37.5)	21(13.5)

The practice of using clean material for tying the cord was relatively high among the control (88%) and intervention (96%) arms. Nearly all women (96% among control and 98% among intervention) used a clean instrument for cutting the cord. Similarly, the practice of drying the newborn soon after delivery was high among control and intervention arms (89% vs. 88%), almost all women (99% among both arms) wrapped the newborn soon after delivery. But, delayed bathing of the newborn for three days or more was only 4% among control arm compared with 47% among intervention arm. Regarding breastfeeding, 47% vs. 55% initiated within the first hour after birth among control and intervention arms respectively. Equal proportions had received BCG (97% vs. 96% among control and intervention arms) and equal proportions (25%) reported newborn illness in the control and intervention arms. But care-seeking for newborn illness was two-and-half times higher among intervention (20%) compared to control arm (8%) (see Table 3).

### Prenatal differences

After building composite variables from discrete responses, all prenatal outcomes were higher in the intervention compared to the control arm, but these were not always statistically significant differences. More than eight in ten (85%) among the intervention arm made three or more antenatal visits compared to seven in ten (71%) of pregnant women among the control. Birth preparation was considered to be adequate among half (51.8%) among intervention women compared with one-fifth (20.8%) among the control. Pregnant women that made health facility delivery were three times higher among the intervention (90%) compared to the control arm (28%). (See Table 4). In the unadjusted models, there is some evidence that the intervention could have a positive effect on the outcomes of interest, with significant differences in birth preparation [2.58(1.00–6.65); *p* = 0.05] and place of delivery [18.21(6.02–55.03); *p*<0.001]. In the adjusted models, only place of delivery [17.94(6.26–51.37); *p*<0.001] remained statistically significant.

**Table 4:** Prenatal and newborn outcome differences between control and intervention women.

Indicator	Control	intervention	uOR(95%CI)	*p*-value	aOR(95%CI)	*p*-value
**ANC visits**						
0–2	218 (28.8)	92 (14.7)				
3/more	540 (71.2)	535 (85.3)	1.57(0.55–4.50)	0.40	1.82(0.65–5.09)	0.26
**Birth preparation**						
Inadequate	600(79.2)	302(48.2)				
Adequate	158(20.8)	325(51.8)	2.58(1.00–6.65)	0.05	2.59(0.81–8.30)	0.11
**Delivery place**						
Home/TBA	540 (72.4)	74 (12.3)				
Health facility	206 (27.6)	529 (87.7)	18.21(6.02–55.03)	<0.001	17.94(6.26–51.37)	<0.001
**Cord care**						
Unclean	530 (71.5)	246 (40.9)				
Clean	211 (28.5)	355 (59.1)	3.0 (1.79–5.13)	<0.001	3.05(1.81–5.12)	<0.001
**Thermal care**						
Inadequate	608 (82.1)	185 (30.8)				
Adequate	133 (17.9)	416 (69.2)	8.10(2.49–26.30)	<0.001	7.58(2.52–22.82)	<0.001
**Breastfeeding & pre-lacteal feeds**						
Inappropriate	255(34.4)	196(32.6)				
Appropriate	486(65.6)	405(67.4)	1.42(0.79–2.57)	0.24	1.26(0.70–2.29)	0.44
**Newborn vaccination status**						
Incomplete	367(49.9)	215(36.6)				
Complete	369(50.1)	373(63.4)	2.16(0.92–5.08)	0.08	1.79(0.89–3.62)	0.89
**Newborn care seeking**						
Untimely	72(37.5)	21(13.5)				
Timely	120(62.5)	135(86.5)	4.27(1.41–12.96)	0.01	4.93(1.59–15.31)	0.006

### Newborn differences

Similarly, all newborn care practices were proportionately higher among the intervention compared to the control arm. However, these differences were not always statistically significant. In the preliminary analysis, nearly two thirds (60%) among intervention women were considered to practice clean cord care compared to one third (30%) among the control women. Seven in ten (69.2%) among the intervention women were categorised as *appropriate thermal care* compared to two in ten (18%) among the control arm. Breastfeeding practices were considered to be similar whereby two thirds among intervention (67%) and control women (66%) were assessed to provide appropriate breastfeeding. Care-seeking for newborn illness was assessed to be timely among 86.5% in the intervention compared with 62.5% among the control women. (Table 4).

In the preliminary analysis, cord care [3.0 (1.79–5.13); *p*<0.001], thermal care [8.10(2.49–26.30); *p* = 0.001], and timely care-seeking for newborn illness [4.27(1.41–12.96); *p* = 0.01] showed significant associations with the intervention. After further analysis, cord care [3.05(1.81–5.12); *p*<0.001], thermal care [7.58(2.52–22.82); *p*<0.001], and timely care-seeking for newborn illness [4.93(1.59–15.31); *p* = 0.006] remained statistically significant. The rest of the outcomes showed odds ratios greater than one but these relationships were not statistically significant. (Table 4).

## Discussion

Although pre-intervention characteristics among the control and intervention arms of the study were comparable, there were significant differences regarding number of pregnancies, number of antenatal care visits and level of HCs. Our study achieved 64% of home visits conducted by VHTs among women allocated to intervention arm while mobile phone consultations were mostly made for labour related conditions and newborn illnesses. There were statistically significant associations between the intervention and care-seeking for health facility delivery and newborn illnesses; thermal and hygienic cord care practices for the newborn. However, there was no statistically significant relationship between the intervention and some prenatal or newborn outcomes such as antenatal care consultations, birth preparation and initiation of breastfeeding within six hours after birth.

Although allocation of HCs was random, the control and intervention arms received uneven proportions of HCs II & III. The control arm had four HCs of level II and four HCs of level III. The intervention arm had two HCs of level II and six HCs of level III. Therefore, pre-intervention differences between control and intervention arms can be explained by variation in the distribution of HC levels in each arm. Data from Health Management Information Systems shows that HCs of level II tend to receive more outpatients including antenatal care consultations compared to HCs of level III. The difference in utilisation between HCs of level II and III explains why there were significantly more women recruited in HCs of level II among the control group compared to HCs of level II in the intervention group. The same argument can be extended to explain significant difference in antenatal care attendance in control arm compared to the intervention arm. These differences could have affected our results. However, random allocation, no decline to participate in the study and inclusion of covariates with significant pre-intervention differences during the analysis this potential limitation could have been mitigated.

Like the UNEST study conducted in eastern Uganda, our study achieved over 50% home visits by VHTs within the prenatal and immediate postnatal period [12]. Our aim was to ensure each mother in the intervention arm was visited at least once, but this was not possible to attain because of large geographical areas in some cases and low incentives for VHTs to travel long distances. Other problems such as interruptions in mobile phone networks and absence from duty stations were highlighted previously [20]. Most common phone calls were made for labour-related conditions and newborn illness such as fever, cough, pneumonia and convulsions. Frequent phone calls for these maternal and newborn conditions explains the significant differences in care-seeking for delivery care and newborn illness among intervention women compared to control women. Statistically significant relationship between the intervention and care-seeking for delivery and newborn illness is corroborated with qualitative findings from the same intervention reported elsewhere [20]. Timely care-seeking was possible in part because VHTs made prior phone calls to professional health workers to alert them of a referral [20]. In the qualitative evaluation of this intervention [20], VHT social status was improved and the referrals that they made were better accepted because the people that they referred to the HCs received prompt care meaning that their trips were not in vain. The improved status of VHTs coupled with prompt care provided to the women helps to improve community confidence in the health system therefore promoting higher utilisation of maternal and newborn care services.

Studies conducted elsewhere in Uganda demonstrated that VHTs successfully implemented a programme for identification and referral of sick newborns [15,31]. Also, in Ghana the *Newhints* study that deployed CHWs to provide health education through home visits showed that the largest change recorded was for care-seeking during ill-health [13]. Care seeking in case of pregnancy and labour is a positive contribution of this intervention because skilled attendant at birth is a proven intervention for improving maternal and newborn survival since most pregnancy complications are known to occur during the perinatal period [1,2]. Similarly, early care-seeking for newborn illness is an important finding because disease progression in newborn babies is rapid. Prompt recognition of newborn illness, early referral and timely care-seeking is likely to avert unnecessary mortality. However, over one in ten of women in the intervention arm still sought for care after 24 hours in case of newborn illness. Delayed care-seeking is likely to jeopardise newborn benefits derived from this study. Future interventions should seek to ensure that the proportion of timely care-seeking especially for newborn illness is elevated through community surveillance system for newborn illness [31].

This study demonstrated that home visits combined with mobile phone consultation with professional health workers encourages women to provide better thermal care and practice hygienic cord care for their newborn babies. These changes in thermal and cord care were important achievements because during the formative stages most women bathed their newborn babies soon after delivery and they applied potentially infectious substances on the cord [16,19]. Exposures to coldness and unhygienic handling of the cord are some of the predisposing factors to newborn morbidity and mortality. Improved newborn care practices are likely to improve newborn survival in Masindi and Kiryandongo. It is also likely that appropriate cord care could be mediated through health facility delivery as earlier shown in the formative study [19]. Also in this study women in the intervention arm were significantly more likely to report health facility delivery further reinforcing the need for promoting health facility delivery.

Although the practice of drying and wrapping of the newborn were comparable among women in both arms of the study, nearly 80% women in the control group had already bathed their newborn within 24 hours. Therefore, early bathing of the newborn is an important driver for inappropriate thermal care for newborns in Masindi and Kiryandongo districts. Future interventions aiming to promote better thermal care should emphasis delayed bathing as a key strategy for behaviour change.

Our study did not record significant differences in birth preparation, exclusive breastfeeding or newborn vaccination although emphasis was laid on all these care practices during home visits. The criterion to assess birth preparation was based on a mother having prepared at least three out of the five possible options. This measure could have been too stringent unlike in a study conducted in India where constituent elements of birth preparation were assessed independently [32]. Indeed, we assessed separately discrete components for birth preparation and found significant differences (results not shown). The offer of pre-lacteal feeds for newborn babies was a deterrent factor for proper implementation of breastfeeding practices. Commonly, women and their families give pre-lacteal feeds based on the assumption that the newborn might be hungry when there is no breast milk coming [16,19]. The phenomenon of offering pre-lacteal feeds merits further investigations in order to device targeted interventions to mitigate it. Regarding newborn vaccination status, we did not demonstrate significant differences partly because vaccination status during the formative stages was high [19]. Therefore, to show small changes in differences between intervention and control arms would require a larger sample size.

WHO recommends five home visits (three prenatal and 2 postnatal). We implemented three visits only (two prenatal and one postnatal). Five home visits, if implemented in a study, may be more effective in raising the rates of women achieving appropriate outcomes. As earlier mentioned [20], attrition among health workers, resulting from further training, workshops conducted in the district headquarters and transfer of health workers coupled with annual and maternity leaves were important bottlenecks. Absence from duty stations affected coordination between VHTs and professional health workers therefore interrupting the permanence and continuity of services for maternal and newborn care. Permanence and continuity of services are important quality measures for general health care including maternal and newborn care [33].

Our study warrants merit because it goes beyond the short messages services (*sms-*based) method of providing educational messages that have been tried in other parts of sub-Saharan Africa [34]. Sms-based approaches for sending educational messages have some limitations: First, few women, especially in rural settings, own mobile phones. Second, in our study context, ethnic diversity is high and so are levels of illiteracy that would make sms-based interventions logistically a daunting challenge. We overcame these potential problems by providing mobile phones to an intermediary group, the VHTs and professional health workers who were based at the HCs. Interaction between VHTs and pregnant women with the possibility of consulting a professional health worker extends the hopes of applying voice mobile phone consultations for improving maternal and newborn health in sub-Saharan Africa. Moreover, the direct cost of making phone calls was about five thousand Uganda shillings per phone per month (equivalent of $2.5 in 2013) is likely to become cheaper, in the long-term, given the declining global costs of acquiring and using mobile phones.

The intervention was a pragmatic community-based randomised trial in which routine health system structures were not manipulated for the intervention to take place [24] implying that findings from this study can be replicated in similar settings.

Unlike a trial study conducted in India [32], our study recorded relatively high loss to follow-up of about 15% vs. 4% in India. However, like in Bangladesh [35,36] the loss to follow-up was comparable with our study 10% and 11% for control and intervention arms respectively. We identified one important reason for loss to follow-up in our study. Half of the population in Masindi and Kiryandongo is comprised of internal migrants and pastoralists. Internal migrants come to work in sugarcane plantations and sugar factory and some are not permanently settled in Masindi and Kiryandongo. Pastoralists tend to move from place to place in search for pasture and water.

Participants providing socially accepted responses could have been a potential bias contributing to differences between control and intervention arms. However, we think that socially accepted answers were minimal because during the qualitative study to evaluate the intervention VHTs and women expressed high level of enthusiasm and willingness to practice recommended maternal and newborn care practices [20]. Data were collected from a few days to six months after delivery. It is possible that women could have forgotten some of the practices such as birth preparation and breastfeeding habits. But, we considered that pregnancy, childbirth and care for the newborn is such a memorable experience that women are likely to recall most of the events that took place around this time.

Selection bias was possible in our study because, recruitment of pregnant women into the study was done consecutively with a cut-off point of 28 weeks of gestation. However, formative study had shown that nearly all pregnant women make at least one ANC visit during their pregnancy therefore giving a chance for all women to be recruited. Nevertheless, the cut-off point of 28 weeks meant that only women who were seeking care earlier could be recruited. However, the cut-off point was justified in this study because sufficient time was needed to make the number of home visits and allow time for the intervention to take effect.

We feared for contamination during the initial stages of randomisation. However, after randomisation we realised that the nearest HCs between control and intervention arms were 10–20 kilometres apart therefore mitigating the problems of contamination.

## Conclusion

VHTs can have an effect in promoting proper cord and thermal care for the newborn and improve timely care-seeking for health facility delivery and newborn illness through home visits, because they could answer questions and refer patients correctly. However, VHTs should have some degree of credibility [20] and should be supported by professional health workers through the use of mobile phones. Community problems such as offering pre-lacteal feeds and early bathing of the newborn should be investigated further and appropriate solutions identified.

## Supporting Information

S1 File Operating procedures for Health Workers. (DOCX)

S2 File Dataset underlying the study. (DTA)

S3 File Trial Protocols. (DOCX)

S4 File Consort Checklist. (DOCX)
