# Supplementary material for: Effect of Village Health Team Home Visits and Mobile Phone Consultations on Maternal and Newborn Care Practices in Masindi and Kiryandongo, Uganda: A Community-Intervention Trial
Source: PLoS One. 2016 Apr 21;11(4):e0153051. doi: 10.1371/journal.pone.0153051 (PMC4839625; doi:10.1371/journal.pone.0153051)
Supplement: S1 File — (DOCX) [file pone.0153051.s001.docx]

**Standard Operating Procedures for Health Workers**

1. Health Worker at the health centre will identify eligible pregnant woman for inclusion in to the study:

***Include if:***

- 1. **Pregnant women making first ANC visit for the current pregnancy**
  2. **All pregnant women found to be up to 28 weeks of pregnancy**

***Exclude if:***

- 1. **Pregnant woman above 28 weeks**
  2. **Pregnant woman found to be bed ridden or mentally disturbed**

1. Health worker will explain purpose of the study and seek for permission to participate (***refer to consent form***)
2. Health Worker will administer a consent form on acceptance (***refer to consent form***)
3. Health worker will open a separate file for each consenting woman and enter details in personal file (***Data collection tool for enrolled mother***)
4. Health worker will fill in all the sections **A**, **B** & **C** of data collection tool for enrolled mother (numbers 1-19)
5. Health worker will fill in the form: **ANC data collection tool** during each prenatal visit made by the pregnant woman
6. Health worker will help pregnant woman to identify a suitable VHT from her village (list of all VHT members, their villages and telephone numbers will be made available)
7. Health worker will notify by a phone call the identified VHT (list of all VHTs and mobile phone contacts will be made available)
8. Health worker will periodically check the patients records to remind VHT about appointment dates for the pregnant woman (calls made at least one week before appointment date)

**Standard Operating procedures for VHT**

1. After the notification phone call from health worker, VHT will visit home of the pregnant woman to make appointment
2. Appointment will depend on availability of woman and other potential caregivers (spouse, auntie, in-laws)
3. The VHT will use available checklist to ensure that all relevant topics are discussed (VHT provides an environment/platform for discussion and NOT Prescription of ideas)[***Using the information checklist for VHTs***]
4. VHT will encourage the woman or potential caregivers to ask questions for clarification (VHT can make a phone call to the health worker for clarity or to reinforce the discussions they have had with the family
5. VHT will note down areas of disagreement in the patients logbook
6. VHT will write in the logbook for the mother to record major concerns

**Information checklist for VHTs**

**Information that will be routinely offered to women and family members:**

**These sessions will be referred to as *‘dialogue sessions’***

**First home visit-(dialogue session one)**

1. ***General prenatal care-prevention and treatment of malaria***
2. Use of nets,
3. Anti-malarial,
4. Folic acid,
5. At least four checkups,
6. Diet and nutrition
7. ***Birth preparation***
8. *Identifying place of delivery,
9. Identify a skilled birth attendant,
10. *Organising transport,
11. *Setting money aside,
12. *Planning for emergency,
13. *Planning with a family member
14. ***Prepare items routinely required for clean delivery***
15. *Clean plastic cloth for delivery,
16. *Clean dry towel for mother and baby
17. *New razorblade
18. *Clean threads
19. *Pairs of gloves
20. ***Danger signs***
21. Vaginal bleeding,
22. Convulsions, fever,
23. Water loss,
24. Abdominal pains,
25. Severe headaches,
26. Blurred vision,
27. Swelling of limbs,
28. Absent or diminished foetal movement)
29. Whenever there are unclear issues regarding pregnancy, the VHT will make phone consultations with the health worker during each visit

**Second home visit-(dialogue session two)**

1. ***Caring for the newborn baby***
2. Skin-to-skin contact,
3. Early breastfeeding,
4. Exclusive breastfeeding,
5. Delayed bathing,
6. Clean cord care,
7. Thermal protection)
8. ***Care seeking in case of ill health*** or feeling unwell, or unusual behaviour of baby, failure to feed, warm to touch, vomiting, discharging umbilicus
9. **During each visit the VHT can make a telephone consultation with the health worker (*please fill in the phone logbook whenever a telephone call is mad*e)**

**Third home visit (within three days post delivery)-(dialogue session three)**

Observe for newborn care practices (Initiation and exclusive Breastfeeding; thermal care; care for the cord) [***please do not be judgemental about what you observe***]

Discuss with mother and other caregivers information on newborn care practices

Inquire if there is any problem/challenge for which they need further clarification (***VHT can call health worker for clarity***)

Encourage regular breastfeeding (every two hours) and avoid other feeds. Discuss family planning and its benefits for the mother and baby.
