# Supplementary material for: Effect of Village Health Team Home Visits and Mobile Phone Consultations on Maternal and Newborn Care Practices in Masindi and Kiryandongo, Uganda: A Community-Intervention Trial
Source: PLoS One. 2016 Apr 21;11(4):e0153051. doi: 10.1371/journal.pone.0153051 (PMC4839625; doi:10.1371/journal.pone.0153051)
Supplement: S3 File — (DOCX) [file pone.0153051.s003.docx]

A community trial to measure the effect of individual prenatal education with phone reminders offered to pregnant women in Masindi and Kiryandongo, Western Uganda

**PhD Full Proposal**

**Candidate:**

Richard Mangwi Ayiasi MBChB (MUK), MPH (ITM)

**Registration number**: 2011/HD07/18278U

Makerere University, College of Health Sciences, School of Public Health

**Supervisors:**

**Prof. Patrick Kolsteren**-University of Ghent/Institute

of Tropical Medicine-Antwerp

**Dr. Christopher Garimoi Orach**-Makerere University

College of Health Sciences-School of Public Health

**Prof. Bart Criel**-Institute of Tropical Medicine-Antwerp

**Institution:**

**A PhD research proposal submitted to Makerere University College of Health Sciences, School of Public Health**

**January 2012**

**Table of contents**

[Acronyms and Abbreviations iv](#_Toc367179421)

[Operational definition of terms v](#_Toc367179422)

[Abstract vi](#_Toc367179423)

[Chapter one 1](#_Toc367179424)

[1. Introduction and Background 1](#_Toc367179425)

[1.1 Neonatal mortality 2](#_Toc367179426)

[i) Global perspective 2](#_Toc367179427)

[ii) Neonatal mortality-the Ugandan perspective 2](#_Toc367179428)

[1.2 MDGs and Neonatal mortality indices 3](#_Toc367179429)

[1.3 Maternal mortality 4](#_Toc367179430)

[1.4 Prenatal educational interventions 5](#_Toc367179431)

[1.5 The District Health System (DHS) in Uganda 7](#_Toc367179432)

[1.5.1 Structure 7](#_Toc367179433)

[1.5.2 Activity packages 7](#_Toc367179434)

[1.5.3 Staffing 7](#_Toc367179435)

[Chapter Two 10](#_Toc367179436)

[2. Review of the literature 10](#_Toc367179437)

[2.1 Antenatal care 10](#_Toc367179438)

[2.2 ANC – a leverage for skilled attendance 10](#_Toc367179439)

[2.3 Delivery of the comprehensive ANC care model 11](#_Toc367179440)

[2.4 Proposed intervention 12](#_Toc367179441)

[2.5 Community-based approaches for maternal and newborn care 13](#_Toc367179442)

[2.6 The role for CHWs 14](#_Toc367179443)

[2.7 Conceptual flaws with CHWs 14](#_Toc367179444)

[2.8 Organisation of CHWs 15](#_Toc367179445)

[2.9 The Village Health Teams: a means for health communication 16](#_Toc367179446)

[2.10 Redefining the role of CHWs 16](#_Toc367179447)

[2.11 Electronic media in health-mHealth 17](#_Toc367179448)

[Chapter Three 19](#_Toc367179449)

[3.0 Problem Statement, Study Justification, Conceptual framework and Objectives 19](#_Toc367179450)

[3.1 Problem Statement 19](#_Toc367179451)

[3.2 Study Justification 20](#_Toc367179452)

[3.3 Conceptual frame and study Objectives 21](#_Toc367179453)

[3.4 Conceptual framework 21](#_Toc367179454)

[3.5 Study Objectives 22](#_Toc367179455)

[3.6 General objective 22](#_Toc367179456)

[3.7 Specific objectives 22](#_Toc367179457)

[Chapter Four 23](#_Toc367179458)

[4.0 Study Methodology 23](#_Toc367179459)

[4.1 Study site and population 23](#_Toc367179460)

[4.2 Sub-study one 24](#_Toc367179461)

[4.2.1 Introduction 24](#_Toc367179462)

[4.2.2 Research Questions 25](#_Toc367179463)

[4.2.3 Objectives 25](#_Toc367179464)

[4.2.4 General 25](#_Toc367179465)

[4.2.5 Specific 25](#_Toc367179466)

[4.2.6 Methodology 25](#_Toc367179467)

[4.2.7 Sample size 26](#_Toc367179468)

[4.2.8 Study variables 26](#_Toc367179469)

[4.2.9 Data collection 27](#_Toc367179470)

[4.2.10 Data analysis plan 27](#_Toc367179471)

[4.2.11 Sub study one-Summary of methodology 29](#_Toc367179472)

[4.3 Sub-study two 30](#_Toc367179473)

[4.3.1 Introduction 30](#_Toc367179474)

[4.3.2 General objective 31](#_Toc367179475)

[4.3.3 Specific objective 31](#_Toc367179476)

[4.3.4 Methodology 31](#_Toc367179477)

[4.3.5 Sample size and technique 31](#_Toc367179478)

[4.3.6 Sampling procedure 32](#_Toc367179479)

[4.3.7 Study variables 33](#_Toc367179480)

[4.3.8 Data collection and analysis plan 33](#_Toc367179481)

[4.3.9 Summary of objectives and methodologies for sub study two 35](#_Toc367179482)

[4.4 Sub study three-the intervention 36](#_Toc367179483)

[4.4.1 Introduction 36](#_Toc367179484)

[4.4.2 General objective 36](#_Toc367179485)

[4.4.4 Methodology 37](#_Toc367179486)

[4.4.5 Study design 37](#_Toc367179487)

[4.4.6 Inclusion and exclusion criteria: 37](#_Toc367179488)

[Exclusion criteria: 38](#_Toc367179489)

[4.4.7 Study variables 39](#_Toc367179490)

[Independent/factor: 39](#_Toc367179491)

[4.4.8 Sample size 39](#_Toc367179492)

[4.4.9 Sampling procedure 40](#_Toc367179493)

[4.4.11 Study profile: sub study three 43](#_Toc367179494)

[5.0 Ethical issues and considerations 44](#_Toc367179495)

[5.1 Study limitations 44](#_Toc367179496)

[5.2 References 45](#_Toc367179497)

[Annex 1 Knowledge and perception of PHC workers 52](#_Toc367179498)

[Annex 2 Interview guide for FGD for sub study one 59](#_Toc367179499)

[Annex 3 interview guide for KIIs for sub study one 61](#_Toc367179500)

[Annex 4 Focal persons for the Key-Informant Interviews for sub study one 63](#_Toc367179501)

[Annex 5 Sampling procedure for sub study two 64](#_Toc367179502)

[Annex 6: data collection guide for Structured Interview for lactating women sub study two 65](#_Toc367179503)

[Annex 7: Key Informant Interview guide with elderly caregivers and traditional birth attendant for sub study two 72](#_Toc367179504)

[Annex 8: Interview guide for In-Depth Interviews with sub groups of lactating women 74](#_Toc367179505)

[Annex 9: Sub study three Flow-diagram for educational intervention 75](#_Toc367179506)

[Annex 10: List of eligible Health Centres by HSD 76](#_Toc367179507)

[Consent form 77](#_Toc367179508)

[Annex 11: Consent form for Health workers and managers 77](#_Toc367179509)

[In case of any doubts please call Richard Mangwi on mobile number 0772 829 377 or John Ssempebwa on 0703 944404 the chairman of the Higher Degrees and Ethics Committee of the School of Public Health Annex 12: Consent form for Elderly care givers and Traditional Birth Attendants 78](#_Toc367179510)

[Annex 12: Consent form for Elderly care givers and Traditional Birth Attendants 79](#_Toc367179511)

[Annex 13: Consent form for prenatal women attending ANC clinic at primary care facility 83](#_Toc367179512)

[Annex 14: Budgets 87](#_Toc367179513)

[i) Budget Sub study one: 87](#_Toc367179514)

[ii) Budget Sub study two: 88](#_Toc367179515)

[iii) Sub study three: Community trial: 89](#_Toc367179516)

[Annex 15: SOPs: Standard Operating Procedure and intervention packages 92](#_Toc367179517)

[i) Standard operating procedure and intervention package for educational intervention during ANC 92](#_Toc367179518)

[ii) Standard Operating procedure for the use of mobile cell phones 94](#_Toc367179519)

[iii) Standard Operating Procedure for Health Assistants based at the health facility 94](#_Toc367179520)

# Acronyms and Abbreviations

ANC Antenatal care

CHW Community Health Worker

DHO District Health Officer

FGD Focus Group Discussions

HA Health Assistant

LMNP Last Normal Menstrual Period

MDGs Millennium Development Goals

MOH Ministry of Health

MS Medical Superintendent

UDHS Uganda Demographic and Health Survey

UNO United Nations Organisation

VHT Village Health Teams

WHO World Health Organisation

# Operational definition of terms

*Primary healthcare facility* The first contact point for formal healthcare - In this case the first contact for which pregnant women access antenatal care i.e. health centres II, III, IV and ANC services at hospitals

*Prenatal service* -as prescribed by the WHO history taking, physical examinations, laboratory investigation, prescriptions, administration of folic acid and Ferrous tablet, assessment for referral, dialogue and discussions about pregnancy, dangers in pregnancy, hygiene, immunisations and birth preparedness

*Technical maternal and newborn services*-more clinical services for example history taking, physical examination like palpation, drawing of blood samples, estimating pelvic adequacy and assessing for referral

*Less technical maternal and newborn services*-services of a promotional nature health education, counselling, birth preparation which entails dialogue and discussions with the pregnant women and their attendants/caregivers

*Individual ANC educational interventions*- information given to a pregnant woman attending ANC based on the unique problems or demands or needs that she presents with during the current pregnancy as opposed to uniform information provided to all prenatal women

*Consistent ANC visits*-timely and completed four ANC visits

*Health worker with a promotional profile* a health worker with a formal employment in the district health system, whose primary training is health prevention and health promotion, does not routinely take part in clinical services like physical examinations, prescriptions of medicines or surgery – in this case we refer to the Health Assistant (HA)

*Homecare practices*-care provided to prenatal women and newborn babies at home based on knowledge and perception of the care givers (elements of care considered in this study are: nutritional care for pregnant women, care for the cord, warmth for newborn babies, and initiation of breastfeeding and neonatal immunisation status)

*Elderly caregivers*-these are usually women in the households, about 50 years or older. They provide neonatal care and advice to family members within the household sometimes extending to the immediate neighbours. As opposed to TBAs, they are not transactional. They provide expert opinion and demonstrate actual care to postnatal women within the household or immediate neighbourhood

*Institutional deliveries*- this term will be considered synonymous with *skilled attendance* and *facility delivery*. It refers to deliveries that take place in formal healthcare facilities in the case of Uganda Health centre II, III, IV or hospital, irrespective of the category of staff conducting the delivery

*Timely return ANC visits*-this is the subsequent visit made within one week of actual date of scheduled appointment during the same pregnancy

# Abstract

**Introduction**: Achieving targets for MDGS 4 and 5 in developing countries has proved elusive. Evidence suggests that a balance between preventive and health promotion interventions during the prenatal period can lead to consistent ANC utilisation and hence increased institutional delivery and reduction of maternal and neonatal mortality. In practice however, antenatal care is organised and delivered in favour of the technical clinical services dwarfing less technical health education interventions, as such, over 90% make first ANC visits but less than 40% make completed four visits, about 40% institutional delivery and less than 20% postnatal visits within six weeks. Evidence from Asia and Africa has demonstrated that community interventions with CHWs visiting individual pregnant women improve maternal and neonatal outcomes. Whether a combined package of prenatal education by CHWs at the primary health care facility and use of standardized mobile phones reminders to pregnant women can lead to improved selected maternal and neonatal outcomes in a low income setting is not known.

**Objective and Hypothesis**: This study aims at measuring the effect of standardized prenatal education interventions offered by Community Health Workers on uptake and utilisation of selected maternal and newborn healthcare services offered at the primary care facility to contribute to the reduction of maternal and neonatal mortality. This study is therefore designed to test the hypothesis that offering a package of standardized prenatal educational interventions at the primary healthcare facility combined with standardized telephone reminders targeting individual pregnant women can lead to timely return ANC visits, completed four ANC visits, increased institutional delivery and improved newborn homecare practices.

**Methodology**: This study will be done in Masindi and Kiryandongo districts in western Uganda. We plan to conduct three sub studies: Sub study one, a descriptive cross-sectional study with healthcare workers and health managers; using structured questionnaires, Focus Group Discussions and Key Informant Interviews. Sub study two is a cross-sectional population based observational study targeting lactating women, traditional birth attendants and elderly caregivers; using Key Informant Interviews with TBAs and Elderly caregivers, In-Depth Interviews and Structured Interviews with lactating mothers. Sub study three a community intervention trial allocating health centres to two different arms (routine ANC care only; routine ANC care and CHWs offering standardized prenatal education at the primary care facility plus standardized mobile phone reminders through CHWs).

**Statistical analysis plan**: Qualitative data will be audio taped, transcribed verbatim, translated in to English and analysed for emerging themes. Quantitative data will be entered in to Epidata 2002 computer software package, cleaned and exported to stata version 10.0 for analysis. Data on knowledge and perception of health workers together with data on common maternal and newborn homecare practices will inform the design for sub study three. In sub study three we will measure timely return ANC visits, completed antenatal care visits, number of institutional deliveries and homecare practices in the two arms. Analysis will be done in stages: univariate to generate frequencies, bi-variate analysis will be done to establish associations between variables of interest. Chi-square tests will be used to establish significant associations; at the Multivariate level, analysis will be run to get Odds Ratio values, 95% CI for the OR and the respective p-values will be presented.

**Dissemination and utilisation**: Results of this study will be shared and discussed with district local authorities and the Ministry of health officials; presentations will be made in national and international conferences and; publications in peer reviewed journals. These findings will specifically inform the ongoing debate on antenatal care service improvement and contribute to the attainment of MDGs 4 and 5.

# Chapter one

# Introduction and Background

Achieving targets for MDGS 4 and 5 in developing countries has proved elusive. Each year 340,000 women die due to pregnancy related causes, half of this being in Africa, while 4 million babies die within the first month of life and an equal number die as stillbirths ([Hogan M, Kyle J F et al. 2010](#_ENREF_40)). In Uganda, maternal mortality is estimated to be 435/100,000 live births while newborn mortality is estimated at 29/1000 live births translating to about 45,000 deaths in the first month of life ([MOH 2010a](#_ENREF_60)). Two thirds of these deaths could have been averted by interventions that are known to be cheap and effective ([Jones G SR, Black RE et al. 2003](#_ENREF_45); [Vishwajeet K, Aarti K et al. 2010](#_ENREF_94)), namely: creativity in health education and behaviour change communication ([Ronsmans C and Wendy J 2006](#_ENREF_79); [UNICEF 2009](#_ENREF_87)); institutional deliveries; healthy behaviours practiced at home and in the communities ([Lawn J, Pyande M et al. 2006](#_ENREF_52)).

Evidence suggests that consistent ANC utilisation leads to increased institutional delivery and reduction of maternal and neonatal mortality ([Ornella L, Seipati Mothebesoane-Anoh et al. 2006](#_ENREF_72); [WHO 2010](#_ENREF_113)). In most developing countries however, the picture is such that over 90% make first ANC visits, less than 40% make completed four visits, about 40% returning for institutional delivery and less than 20% postnatal visits within six weeks. In Masindi and Kiryandongo districts, for example, while first ANC attendance is about 90%, completed ANC visits is 30% and institutional deliveries 23.5% ([DHO 2010](#_ENREF_28)).

Improvement in institutional delivery requires a balance between preventive and health promotion interventions during the prenatal period. In practice however, antenatal care is organised and delivered in favour of the technical clinical services dwarfing equally important less technical health education interventions.

The intricacies between maternal and newborn health is evident and a coordinated continuum of care is one such approach to simultaneously address the challenge of maternal and newborn deaths ([WHO 2005](#_ENREF_107); [Bahl, Qazi et al. 2010](#_ENREF_4); [WHO 2010](#_ENREF_113)). Reduction of maternal and newborn deaths to levels stipulated by the Millennium Development Goals (MDGs) calls for integration of maternal, newborn and child health interventions across the continuum of care ([WHO 2005](#_ENREF_107); [WHO 2010](#_ENREF_113));

broadly defined in two dimensions-as the *time of care* – prenatal to post-natal and *place of care-*household and communities to health facility ([de Graft-Johnson, Kerber et al. 2006](#_ENREF_26)).

In its current form of implementation the integrated comprehensive care model in ANC has laid excessive emphasis towards more technical services such as measuring blood pressure and screening for HIV/AIDS, dwarfing equally important educational interventions. Meanwhile, the use of *Community Health Workers* programs with the potential to enlist community participation and empower communities ([Barber and Gertler 2008](#_ENREF_7)) has so far yielded controversial results ([Bhutta Z, Zohra S et al. 2010](#_ENREF_12)). Gross imbalances exist between technical clinical services on the one hand and educational interventions on the other with the technical services predominating maternal and newborn care at the primary care facilities.

A critical challenge for health systems in sub Saharan Africa today regarding maternal and neonatal health is how best to package and deliver the known interventions to the households in a manner that is effective and acceptable to the ordinary population ([Bhutta Z and Seema 2005](#_ENREF_11); [Freeman P, Perry H et al. 2009](#_ENREF_31); [Shiffman J 2010](#_ENREF_81)) in other words bridging the ‘know-do’ gap ([Bhutta Z and Seema 2005](#_ENREF_11)).

## 1.1 Neonatal mortality

## i) Global perspective

The slow decline of under-five mortality in sub Saharan Africa ([Haines, Sanders et al. 2007](#_ENREF_35); [WHO 2009](#_ENREF_112)) is attributable to a stagnated neonatal mortality ([WHO 2008a](#_ENREF_110)). Neonatal mortality in sub Saharan Africa makes significant contribution towards child mortality; it constitutes two thirds of deaths among children one year and below ([Kapp 2008](#_ENREF_46)); and up to 45% of deaths among children below five years ([WHO 2008a](#_ENREF_110)). Leading causes of neonatal mortality in Africa have been well documented ([Renay W, Ronsmans C et al. 2003](#_ENREF_77)), at the same time efficacious interventions with potentials to mitigate them have equally been prescribed ([Dharma S, Osrin D et al. 2004](#_ENREF_27); [Costello, Filippi et al. 2007](#_ENREF_21); [Baqui A, Shams El-Arifeen et al. 2008](#_ENREF_6); [Kapp 2008](#_ENREF_46); [WHO 2008a](#_ENREF_110); [Perry H, Freeman P et al. 2009](#_ENREF_75)). To make these low cost interventions available to those who need them most poses a major policy gap ([Bhutta Z and Seema 2005](#_ENREF_11); [Costello, Filippi et al. 2007](#_ENREF_21)).

## ii) Neonatal mortality-the Ugandan perspective

The newborn mortality in Uganda is estimated at 29/1000 live births translating to about 45,000 deaths in the first month of life ([MOH 2010a](#_ENREF_60)). About 82% of these deaths is caused by three conditions: sepsis (32%); birth asphyxia (26%) and; low birth weight (25%); while maternal mortality is estimated to be 435/100,000 live births with major causes of maternal mortality in Uganda identified as haemorrhage (30%), infection (19%), obstructed labour (10%), pregnancy induced hypertension ( 15%) and abortions (16%) ([MOH 2010a](#_ENREF_60)).

Uganda covers an area of 241,000 square kilometres with a population of 32.2 million inhabitants and population density of 133 inhabitants per square kilometre and a rural population of 80%. The total fertility rate is 6.7 annual growth rate of 3.2% per annum and contraceptive prevalence rate of 24%. Under five mortality of 137/1000 live births; infant mortality is 75/1000 live births. About 72% of the population lives within 5km radius from the nearest health facility and 61% of the approved position for the workforce that is filled ([MOH 2010b](#_ENREF_61)) .

The current national health policy document for Uganda ([MOH 2010c](#_ENREF_62)) underscores the roles that promotive and preventive services can play in increasing access to essential services. This policy document further elaborates the need to ensure that people understand their health rights and responsibilities and improve awareness by causing change in attitudes, practices and behaviours. Various national policy documents ([MOH 2010a](#_ENREF_60); [MOH 2010b](#_ENREF_61); [MOH 2010c](#_ENREF_62)) have laid specific emphasis on the continuum of care both across time period (pregnancy-delivery-post-natal) and across various levels of care (community-first referral and second referral level).

## 1.2 MDGs and Neonatal mortality indices

In September 2000, world leaders met in New York and endorsed the famous millennium declaration ([UNO 2000](#_ENREF_88)) which culminated in to the eight Millennium Development Goals (MDGs). MDG 4 specifically aims at reducing by two thirds the indices of child mortality (referring to 2000 indicators) by the year 2015. Five years away, sub Saharan Africa has not shown significant reduction even when global child mortality has reduced over the last decade. This stagnation in child mortality has been largely attributable to the neglect for neonatal survival interventions ([Shiffman J 2010](#_ENREF_81)).

Indicators regarding neonatal mortality are, first of all, conspicuously missing in the Millennium Development Goals indicators; secondly, reproductive health programs until recently, have placed emphasis on maternal and child health issues ([Shiffman J 2010](#_ENREF_81)) ignoring the unique importance of neonatal survival in reducing child morbidity and mortality ([Bang A, Bang R et al. 1999](#_ENREF_5); [Shiffman J 2010](#_ENREF_81)).

Thirdly, because most deliveries still take place at home very little information is available to communicate the magnitude of neonatal morbidities and mortalities ([Victora C, Craig E R et al. 2010](#_ENREF_91)).

In the absence of concrete data, neonatal morbidity and mortality as a major driver of child mortality, for a long time, remained invisible; moreover most neonates die in the first 24 hours of life without a name let alone a birth certificate ([WHO 2008a](#_ENREF_110)) and; fourth, traditionally, neonates are considered a transition towards real personhood. At this stage the mother and baby are considered unclean and so in case of ill health they seek traditional care ([Nirmala N, Prasanta T et al. 2010](#_ENREF_68)). Fifthly, amidst all these challenges health systems in sub Saharan Africa have also been weak fragmented and ill prepared ([Schellengberg J A, Victoria C G et al. 2003](#_ENREF_80)) to address particularly the preventive and promotional aspects of healthcare ([Haines, Sanders et al. 2007](#_ENREF_35)).

## 1.3 Maternal mortality

Global maternal mortality has reduced but in sub Saharan Africa the trends have either stagnated or got worse ([WHO 2006b](#_ENREF_108); [WHO 2008b](#_ENREF_111)). Of all global maternal deaths, 99% are in the developing countries ([WHO 2006b](#_ENREF_108); [Zarocostas 2009](#_ENREF_118)). Given the current rate of MMR decline of less than 1% per annum ([WHO 2007](#_ENREF_109)), which falls short of the proposed rate of 5.5% per annum ([WHO 2008b](#_ENREF_111)), MDG targets will not be met ([Zarocostas 2009](#_ENREF_118)).

The high maternal deaths occurs at the time when the causes of maternal deaths are well known with available and cost-effective strategies of preventing them ([WHO 1996](#_ENREF_102); [WHO 2006b](#_ENREF_108)). Direct medical causes of maternal deaths have been well documented; haemorrhage, hypertensive disorders in pregnancy (HDP), sepsis ([Hoestermann C, Ogbaselassie G et al. 1996](#_ENREF_39); [Mutyaba S and Mmiro F 2001](#_ENREF_64)) contributing to about 60% of maternal deaths with 43% of the deaths occurring within the first 24 hours post partum ([Hoestermann C, Ogbaselassie G et al. 1996](#_ENREF_39)).

There is evidence to suggest that early initiation of ANC ([Ochako R, Fotso J et al. 2011](#_ENREF_71)) combined with at least four attendance improves the uptake of skilled attendance at birth ([Nikiema B, Beninguisse J et al. 2009](#_ENREF_66)). Skilled attendance at birth as a cheap and affordable solution has persistently remained very low even with increased availability of services across many developing countries ([Magoma M, Requejo J et al. 2010](#_ENREF_56)). Studies across developing countries have shown that skilled birth attendance at delivery can be significantly increased by interventions such as community mobilisation and behavioural change communications ([Daly and Saadah 1999](#_ENREF_22); [Mikey R, Glenn L et al. 2008](#_ENREF_58); [Newlands D, Yugbare-Belemsaga D et al. 2008](#_ENREF_65); [Xiaoning L, Hong Yan et al. 2010](#_ENREF_116)).

## 1.4 Prenatal educational interventions

Underlying challenges with achieving prenatal educational interventions are numerous: inadequate counselling during antenatal, limited access to care even when danger signs are recognised early and conflicting traditional care practices ([MOH 2010a](#_ENREF_60)).

Even when up to 97% of women attend ANC visit at least once, only 17% make the first visit in the first trimester; 34% of them initiate it late in the third trimester while 47% attend all the four recommended visits ([UDHS 2006](#_ENREF_85)). Late initiation and incomplete attendance of ANC visits at the health facility significantly compromise efforts of health education and counselling for prenatal women.

The high drop-out rates in ANC can, in many ways, be compared to high drop-out rates seen in pre-ART clinics attributed to low counselling at the various clinics. One such study conducted in Uganda showed that educational interventions and counselling is generally lacking in many health facilities ([Lubega, Nsabagasani et al. 2009](#_ENREF_55)) leading to high drop-out rates during HIV/AIDS care. The same argument could be extended to maternal and newborn health (personal communication).

Similar findings were shown in Tanzania ([Magoma M, Requejo J et al. 2010](#_ENREF_56)) where lack of sufficient educational interventions during ANC was associated with low utilisation of skilled delivery at birth. Some of the reasons advanced for the low counselling in health facilities are explained by the primary focus of health workers being on disease and its management rather than on people and their wider environment.

Studies conducted in Asia ([Choudhury and Ahmed 2011](#_ENREF_19)) among the ultra-poor populations suggests that specific sub-class of people required a tailored educational intervention in order to attain a behaviour change in the uptake of pre-and post-natal services.

In Uganda there is the on-going work on the “Uganda Newborn Survival Study” in the Iganga-Mayuge Demographic Surveillance Sites (DSS), under the stewardship of Makerere University School of Public Health in collaboration with *Karolinska Institutet* ([Waiswa P 2010](#_ENREF_96)). It explored newborn care practices in communities and the formal healthcare system making major revelations: low uptake of newborn care practices in the homes especially among the poorer segments of communities, delay in seeking for care associated with delays in recognition of symptoms and homecare practices that are potentially harmful to the newborn. These findings provide us with relevant information on what to do, but does not tell us *how to do it.*

Available data about the prevalence and determinants of potentially harmful newborn homecare practices ([Byaruhanga R, Nsungwa-Sabiiti et al. 2010](#_ENREF_15); [Waiswa P 2010](#_ENREF_96)) in Uganda, point to care practices being deeply rooted in culture and beliefs.

These studies recommend that to effect change in behaviour requires interventions early in pregnancy. However, the study by Waiswa et al ([Waiswa P, Stefan P et al. 2010](#_ENREF_99)), was conducted in a small and homogenous population of the DSS compromising generalizability of its results.

In part, this study aims to collect data on prevalent homecare practices (maternal nutrition for pregnant women; thermal care, care for the cord and pre-lacteal feeds for the newborn) and their rationale among an ethnically diverse (multi-lingual, multi-national) population of Masindi and Kiryandongo districts; identify harmful practices to be discouraged and healthy ones that can be promoted.

This study seeks to identify challenges with delivering prenatal educational interventions at the primary care facility; assess the rationale for homecare practices in the community and use these findings to design and deliver tailored educational intervention to suit individual needs for pregnant women visiting ANC clinics. This approach will ensure appropriate and focused prenatal messages with the potential to achieve timely return ANC visits, institutional deliveries, improved prenatal and newborn homecare practices, ultimately contributing to the reduction in maternal and neonatal mortality.

This study will overcome the limitations encountered by a small and homogenous population of the DSS in Iganga-Mayuge. Findings from this study will also augment the works of Nabiwemba in establishing a proxy community-based estimation of low neonatal birth weights and the one of Nalwadda in the roles of CHWs in the identification and referral of sick neonates (personal communications) contributing to neonatal survival and mortality reduction.

## 1.5 The District Health System (DHS) in Uganda

## 1.5.1 Structure

In Uganda the health system is typically organised on the basis of a decentralised structure of the District Health System (DHS) as an autonomous unit with the operational or implementation unit being the Health Sub District (HSD) (*see figure 1*). The district health system therefore comprises of a District Health Office; Health Sub District and a network of health centres. The district health system is headed by a District Health Officer (DHO); he is assisted by a team of health professionals who together constitute the District Health Team (DHT).

At the second level, the DHS is made of a series of Health Sub Districts (HSDs) whose administrative structure resembles that of the DHT (comprised of a Medical superintendent, hospital administrators, a nursing officer, accountant and personnel officer). The HSD is made up of a satellite of smaller health units usually referred to as Health centres (HC). Health centres are designed to offer a minimum package of activity of which ANC and delivery services are part.

## 1.5.2 Activity packages

Health centres are further categorised as HC II, III or IV depending on the activity package that it should deliver.

The HC II is essentially an outpatient unit with curative consultation and ambulatory care. Increasingly, however, the health centre II level provides ANC care and conducts deliveries for pregnant women and where a DHS deems it necessary they deploy a qualified midwife in addition to the other staffs. Meanwhile, the HC III is expected to provide outpatients consultations, with an inpatient bed capacity of about 15 beds. It provides laboratory diagnostics, ANC and delivery of pregnant women.

## 1.5.3 Staffing

Throughout the hierarchy of the district health system (DHT, HSD, HC) there exists a unique group of health workers who are non clinical and more promotional in nature. These are the ‘*Health Inspectors’* and the ‘*Health Assistants’*: the DHS is expected to deploy a District Health Inspector (DHI) at the district and HSD levels; a health assistant at each of the health centre II & III. The health inspectors and health assistants are a specific cadre whose training curriculum is basically health promotion and health education.

In practice Health Inspectors and Health Assistants participate in a wide range of health issues at the district ranging from health education to home improvements and hygiene. These groups of cadres found more relevance during the colonial and post-colonial era where hygiene and sanitation was enforced through bylaws by the colonial and immediate post-colonial governments.

Although the posts of *health assistants* have remained on the health work force structure in Uganda their role is no-longer well appreciated hence they are usually not appropriately deployed rendering them underutilised and redundant in most cases (personal communications). Yet they could be optimally utilised to deliver health relevant information to pregnant women at the health facilities. They have been extensively utilised in CBDOTS TB programs, EPI and improving latrine coverage which are organised under vertical programs, but have not been specifically engaged for maternal and newborn care services.

This intervention seeks to engage Health Assistants, who have a promotional profile, at the health centres that are providing ANC and delivery care.

The health assistant will be assigned to organise and supervise a team of CHWs who will be providing educational interventions to prenatal women attending ANC clinics; he will ensure that the pregnant women attending ANC clinics receive consistent messages ([Hill, Manu et al. 2008](#_ENREF_38)); that there is continuity of information between the health centre and CHWs in the communities, reinforcing this continuum by the use of mobile cell phones.

Information provided at the primary care level will include, information about pregnancy, birth plans and care for the newborn, emergency conditions and emergency responses ([WHO 2002b](#_ENREF_104)) and; information and dialogue on specific health challenges that a pregnant woman will present with.

Figure 1 **DHS structure**

Figure 1 elaborates structure of the district health system illustrating the position & distribution of the health workers with a promotive profile (Health Inspector, Health Assistant and Medical Social worker).

Ministry of Health

**Central Level**

District Medical Office

**DHS managerial level-District**

**Public Health Specialist**

**District Health Inspector**

**HSD Health Inspector**

**DHS operational level-HSD**

**Medical Social Worker**

**Public Health Nurse**

Private-For-Profit clinics

Private-Not-For-Profit Health centre III

Public health centre III

**Health Assistants**

Private-Not-For-Profit Health centre II

Public health centre II

**PHC facility-** Provides MAP to Community

**Community Health Workers/village health teams**

**Traditional Birth Attendants**

**PHC**-Primary Health Care facility

**LHS**-Local Health System

**HSD**-Health Sub-District

*Source: author*

# Chapter Two

# Review of the literature

## 2.1 Antenatal care

Previous reviews have been critical about the provision of ANC care as a screening tool for risk mothers ([Carroli, Rooney et al. 2001](#_ENREF_16); [Carroli, Villar et al. 2001](#_ENREF_17); [Villar J, Carroli G et al. 2001](#_ENREF_92); [Villar, Ba'aqeel et al. 2001](#_ENREF_93)). Subsequent recommendations maintained ANC as an important public health intervention but should have an orientation towards the provision of a comprehensive package ([WHO 2002a](#_ENREF_103); [WHO 2002b](#_ENREF_104)). Two mutually reinforcing interventions should be delivered in the comprehensive care package during ANC: **1)** The more technical intervention which includes history taking, clinical examinations, laboratory investigations, treatment and assessment for referral ([WHO 2002a](#_ENREF_103)) and; **2)** Health education and counselling which entails dialogue that creates an interface between medical conditions and socially relevant issues regarding ANC: promotion of healthy lifestyles among pregnant women; birth plans including preparation for unexpected events; preparation for parenting especially caring for the newborn baby ([WHO 2005](#_ENREF_107)). This process of communication maintains the link between individual prenatal women and healthcare providers. One such communication strategy has been CHWs interventions.

## 2.2 ANC – a leverage for skilled attendance

The presence of a health professional during the entire continuum of care for maternal and newborn care, more especially during intra-partum, has the greatest potential of reducing maternal and neonatal mortality and morbidities ([WHO 2010](#_ENREF_113)). Significant reduction in maternal and neonatal mortality can be achieved through early initiation and consistent attendance of four targeted ANC visits ([Ornella L, Seipati Mothebesoane-Anoh et al. 2006](#_ENREF_72)), institutional deliveries and postnatal care in the first seven days following delivery ([Warren C, Daly P et al. 2006](#_ENREF_100)). Consistent ANC attendance brings additional advantages: use of insecticide treated bed nets; uptake of IPT and PMTCT services and; exclusive breast feeding ([Agho E, Dibley M et al. 2011](#_ENREF_1)).

The provision of preventive and promotional care to pregnant woman and the newborn baby across the continuum therefore requires that more technical services, educational interventions and counselling are given a balanced attention ([WHO 2002a](#_ENREF_103); [WHO 2002b](#_ENREF_104)). In practice however, attempts to provide preventive and promotive services to pregnant women and newborns at the primary care level tend to take a mechanical dimension. Health workers mainly concentrate on measuring and recording a series of clinical observations concluded with filling in the ANC card. No deliberate efforts are taken to interpret or discuss findings and its implications for the mother, her pregnancy or/and the newborn ([Titaley C, Hunter C et al. 2010](#_ENREF_84)). The same observation is made among health workers using other preventive and promotive interventions like the growth-monitoring program ([Roberfroid D, Lefevre P et al. 2005](#_ENREF_78)).

Health workers make the assumption that the information available in the ANC card is difficult science and therefore incomprehensible to the ordinary patient ignoring the fact that women understand better what is going on (right or wrong) with their pregnancies or their newborn babies. Because of the absence of a clear communication from the side of the health worker, other system-wide challenges notwithstanding, pregnant women and their families do not appreciate the necessity for regular ANC visits, institutional deliveries and post-natal attendance ([Magoma M, Requejo J et al. 2010](#_ENREF_56)).

To the contrary, ANC is utilised by some to confirm pregnancy ([Choudhury and Ahmed 2011](#_ENREF_19)), while for others possession of an ANC card among pregnant women is understood to be a ‘passport’ for gaining access to the health facility in case of unforeseen emergency health facility delivery (personal communications).

## 2.3 Delivery of the comprehensive ANC care model

Improving maternal and newborn survival by the delivery of an integrated comprehensive healthcare package through a continuum of care has been widely endorsed and advocated for ([WHO 2010](#_ENREF_113)). In this model a primary healthcare worker provides a series of both clinical and promotional interventions to a pregnant mother during ANC, through the intra-partum at delivery to the postnatal period.

Unfortunately, most pregnant mothers for varied reasons ([Waiswa P, Kemigisa M et al. 2008](#_ENREF_97)), attend the first antenatal visit and never return for subsequent visits, rarely completing the predetermined four visits for the entire gestation period. Moreover, many of them make the first visit late in to the second or third trimester ([WHO 2008a](#_ENREF_110)). This late attendance coupled with high attrition rates in ANC present a ‘missed opportunity’ for care-seeking mothers to receive a comprehensive package of preventive and promotional services.

Skilled attendance at birth although widely advocated for, amidst several health system challenges, has equally not gained any significance in sub Saharan Africa ([Waiswa P, Peterson S et al. 2010](#_ENREF_98))

A skilled attendant as defined by WHO is an accredited health professional, such as a midwife, a doctor or nurse ([WHO 2004](#_ENREF_114)). Inherent in this definition is the central role of the skilled attendant in the health system ([WHO 2004](#_ENREF_114)) acting as a link between the community and the tertiary level of care. During labour, only about 46% of the expected mothers get in to some contact with the formal healthcare system ([WHO 2009](#_ENREF_112)), a contact time that is usually inadequate to deliver all the important promotional and preventive health messages.

On the other hand, pregnant women and their newborn babies who never make any formal contacts with the healthcare system during the time of labour are faced with challenges related to practices and beliefs ([Choudhury and Ahmed 2011](#_ENREF_19)) such as early bathing of the baby, late initiation of breastfeeding, unhygienic practices ([Waiswa P, Kemigisa M et al. 2008](#_ENREF_97)) that are likely to jeopardise the health of both mother and the newborn. Moreover these risky practices are not just limited to the lower socio-economic class but have been shown to apply across all social classes ([Waiswa P, Peterson S et al. 2010](#_ENREF_98)).

## 2.4 Proposed intervention

This study is premised on the fact that educational interventions during ANC services constitute an integral component of maternal and newborn care and should therefore receive equal attention during ANC service delivery. Experiences from both Low- and High-income countries where prevention and promotional interventions emphasize educational interventions the results have been in favour of educational intervention strategies ([Nyonator K, Awoonor J et al. 2005](#_ENREF_70); [Bhutta Z, Sajid S et al. 2011](#_ENREF_10)).

This study aims to assign the task of overseeing health promotion at the health facility to a health worker with a profile that is largely promotional in nature (Health Assistant) and linking them to Community Health Workers-(known as Village Health Teams in Uganda); enhance linkages between the *professional health worker* and CHW by the use of mobile cell phones that CHWs can access to call and communicate with clinical staff based at the health facility.

We therefore propose the engagement of Community Health Workers providing educational interventions on the one hand and leaving a trained healthcare worker to concentrate on the more technical aspects of ANC on the other; while emphasising the synergy between the two in contributing towards maternal and neonatal survival; both services being delivered at the primary care facility.

Our intervention methodology is largely inspired by successful community interventions conducted in Ghana ([Nyonator K, Awoonor J et al. 2005](#_ENREF_70); [Kirkwood B, Manu A et al. 2010](#_ENREF_50)). Both these interventions conducted formative studies that guided the future implementation and used a consultative approach to gain consensus among national and sub national actors ([Hill, Manu et al. 2008](#_ENREF_38)).

With this arrangement, health workers are capacitated to directly communicate with CHWs supporting them in the decision-making capacity regarding prenatal women and newborns; make inquiries about the health of mothers and their babies, also provide reminders for return visits and due dates for deliveries.

We envisage that the presence of a specific team with a promotional profile offering educational interventions alongside a more technical health worker at the primary care facility augmented by the availability of mobile phones at the community level will stimulate prenatal care seeking, enhance uptake and utilisation of antenatal care services, institutional deliveries, improved maternal and newborn homecare practices.

## 2.5 Community-based approaches for maternal and newborn care

Another widely advocated model of care delivery for maternal and newborn survival has been community-based interventions delivered by Community Health Workers ([Thea D and Qazi S 2008](#_ENREF_83)) (CHW-referred to differently in different regions). CHW are widely used to deliver a spectrum of care packages, both preventive and promotional, sometimes curative ([Haines, Sanders et al. 2007](#_ENREF_34)) for example distribution and administration of allopathic medicines, FP methods and counselling, occasionally administering injections.

In the last decade, the use of Community health workers as a concept has once again featured within global policy debates ([Haines, Sanders et al. 2007](#_ENREF_34); [Anonymous 2010](#_ENREF_2)) in part resulting from the workforce crisis across most of sub Saharan Africa ([Kober K and Van Damme 2006](#_ENREF_51)) but also the fact that community health workers programs have demonstrated the ability to improve access to and coverage of healthcare services ([Bhutta, Samana A et al. 2008](#_ENREF_14); [Perry H, Freeman P et al. 2009](#_ENREF_75); [Cellette, Wright et al. 2010](#_ENREF_18); [Lewin S, Munabi-Babigumira S et al. 2010](#_ENREF_54)) especially in poor underserved areas.

## 2.6 The role for CHWs

However the full recognition of the community as an important actor in the process of achieving community participation has been largely marginalised ([WHO 2010](#_ENREF_113)). The extent to which community-based interventions using community health workers can contribute to an improved neonatal outcome has also been contested ([Shiffman J 2010](#_ENREF_81)) rather questioning the contextual factors that could enhance success of such programs ([De-Brouwere V, Fabienne R et al. 2010](#_ENREF_25); [Shiffman J 2010](#_ENREF_81)).

The use of community-based interventions across the world especially in low-income countries has displayed a sine-wave pattern over the last four decades. Although conceived over 50 years ago, it gained prominence after the Alma Ata declaration, but went in to oblivion in the 1990s consequent to the Structural Adjustment programs (SAPs); and re-emerges with the acute shortages in workforce for health prominent in sub Saharan Africa.

## 2.7 Conceptual flaws with CHWs

The historical evolution of CHWs is many times influenced by the local political context in a country ([Haines, Sanders et al. 2007](#_ENREF_35)) in Tanzania for example it evolved round the concept of African socialism with the slogan “A fair share of the little we have”, while in Zimbabwe, it gained prominence within the struggle for independence.

Conceptually, the general consensus is that Community health workers interventions are efficacious but have largely been a failure when attempts are made to take them to scale ([Nyonator K, Awoonor J et al. 2005](#_ENREF_70)). Reasons for failures of these programs range from organisational challenges ([Nyonator K, Awoonor J et al. 2005](#_ENREF_70)) to the poor understanding and interpretation of the term ‘community health workers’- many times used to mean Primary Health Care ([Heggenhougen and Magari 1992](#_ENREF_37)).

Roles of CHWs across countries are indeed varied; it is not possible to precisely tell what a community health worker does. This ambiguity in their roles ([Clarke, Judy D et al. 2008](#_ENREF_20)) makes it difficult to recognise an individual as a community health worker. Some people have further complicated their functions by referring to them as *generalist* and others *specialist-* ([Lewin S, Munabi-Babigumira S et al. 2010](#_ENREF_54)) terminologies that are themselves misleading. Ambiguity in role definition pits CHWs in to conflict with formal health workers leading to unhealthy competition between the two ([Frankel 1992](#_ENREF_30)) instead of the anticipated complementarities between them ([Haines, Sanders et al. 2007](#_ENREF_35)).

Training for CHW is ad hoc and conducted by health workers who already have a curative bias towards healthcare and little time to supervise them ([Frankel 1992](#_ENREF_30)). Meanwhile, attachments for practical sessions are done at the health centre and hospital without any contact with communities where deployment is expected ([Nyonator K, Awoonor J et al. 2005](#_ENREF_70)) . Although most trainings are intended primarily for preventive and promotional services, the curative training supersedes initial intentions ([Frankel 1992](#_ENREF_30)).

## 2.8 Organisation of CHWs

A major challenge with the use of community health workers has been the rather vertical approach dictated by specific disease control programs ([Oystein E 2010](#_ENREF_73)) and their subsequent isolation from formal healthcare systems. This is further aggravated by the unrealistic voluntary connotation attached to their function widely viewed to be a cheap intervention meant for poor inaccessible communities ([Uta L and Sanders D 2007](#_ENREF_89)).

Voluntarism among poor communities is an unrealistic expectation especially when expected to perform a ‘compulsory’ job ([Glenton C, Scheel I et al. 2010](#_ENREF_32)) like attending to a mother in the immediate postnatal period. CHW are usually people in the community who need and must earn a living; the concept of voluntarism therefore severely undermines the very survival of CHW interventions.

On the global scene we observe that even high-income countries for example Belgium with universal access have still retained the ‘old fashion’ of CHW concepts with home visits and communal education programs regarding child survival ([*Kind-en-Gezin* 2009](#_ENREF_48)). In Belgium these community-based structures were established in the early 1900s and still find relevance to this day.

Evidence mainly from Asia ([Bang A, Bang R et al. 1999](#_ENREF_5); [Dharma S, Osrin D et al. 2004](#_ENREF_27); [Baqui A, Shams El-Arifeen et al. 2008](#_ENREF_6)) has shown that community based interventions through trained community health workers can significantly improve neonatal survival, but very scant information about this mode of intervention for neonatal survival is available for sub Saharan Africa, in fact such studies to the magnitude and scale in Asia have not been tried in sub-Saharan Africa ([Siddhartha G and Harshpal S 2010](#_ENREF_82)). However, interventions using CHW in the provision of care to HIV patients in sub Saharan Africa have shown significant improvement in access and coverage ([Cellette, Wright et al. 2010](#_ENREF_18)).

## 2.9 The Village Health Teams: a means for health communication

In Uganda, community health workers are grouped under the label ***Village Health Team*** (VHT). The government of Uganda through its Ministry of Health has adopted and invested in the Village Health Team strategy (VHT) since 2001 as a complementary healthcare delivery model. VHTs have a mandate of advocacy, social mobilisation and communication. This policy framework has generated a renewed interest in the VHT program in Uganda. Within the current policy framework the Ministry of Health in Uganda and its partners UNICEF, WHO and UNFPA have re-launched the VHT strategy to achieve MDGs 4 and 5 in Uganda by the year 2015 ([MOH 2010a](#_ENREF_60)).

A recent study provides insight in the VHTs situation in Uganda ([MOH 2009](#_ENREF_59)). It identified the following challenges: **1**) weak administrative structure for the VHTs, **2**) absence of supportive supervision and feedback mechanisms.

This review recommends that VHT programs must remain focused and operate in collaboration with the formal healthcare system that provides a minimum package of activities, emphasising linkages with related sectors in the district. It remains however vague on *what* the VHTs should do in order to improve maternal and neonatal health, and *how* they should do it.

## 2.10 Redefining the role of CHWs

This intervention will therefore identify a health worker at the health centre with a promotional background, separate from the technical midwife, who will organise and supervise a team of CHWs within its catchment population (*see figure 1*). He/she will be responsible for health education schedules and sessions at the health facility, supervision of CHWs in the community, organising regular follow-up sessions with CHWs to discuss problems and find local adapted solutions.

Creation of such a structure for health promotion activities with a clear leadership and regular supervision will mitigate some of the challenges already identified with functioning of CHW programs: It has the potential to create a professionalised health promotion team visible to the general public and giving them a greater credibility and hence acceptance by the community.

These community workers need a well defined place in the healthcare delivery system; should obviously be linked to the formal healthcare structure ([Freeman P, Perry H et al. 2009](#_ENREF_31)) and not operate in isolation; increase their contact with the curative system through the use of novel techniques like mobile telecommunications and toll free lines and regular follow-up sessions.

A deliberate enhancement of the collective character and function of a ‘team’ within the VHT ([Vanlerberghe V, Toledo M et al. 2009](#_ENREF_90)) that creates visibility ([Cellette, Wright et al. 2010](#_ENREF_18)), emphasises the specific identity of the VHT and promotes the social recognition of their role in the healthcare system; that VHTs’ knowledge and skills have to be enhanced through continuous training and supervision sufficient enough to make them ‘experts’ in providing basic information on maternal and neonatal health without necessarily conflicting their roles with health professionals.

## 2.11 Electronic media in health-mHealth

The use of mobile phones as a means of communication is thought to be the fastest growing technology worldwide with 90% of the world’s population expected to have access to mobile phones by the end of 2010; 65% of these are expected to be in developing countries ([mhealth-Alliance 2011](#_ENREF_57)). The use of mobile phone technologies-*m*health can augment the link between communities and the formal health care system.

Increasingly the use of mobile phones in health, *m*health, has extended to make medical appointments mainly in the developed countries. The advantages with mobile phones is that the cost of owning and using mobile phones has gradually reduced over the years to less than ten percent of the cost ten years ago ([Noordam A, Kuepper B et al. 2011](#_ENREF_69)). It can reach to the most rural of users to empower the public with information and enables remote health workers to reduce inefficiencies ([Isobel 2011](#_ENREF_44)).

In Uganda the coverage of mobile phones is estimated at eight million mobile phone subscribers and access is estimated at 85% including kiosks and access through relatives and neighbours ([UDHS 2006](#_ENREF_85)).

*M*Health has been applied in providing care to people living with HIV and AIDS with a move to test its use in maternal health services in India, Bangladesh and South Africa ([Isobel 2011](#_ENREF_44)) empirical evidences of its use in maternal and newborn health services in Africa is still lacking ([Noordam A, Kuepper B et al. 2011](#_ENREF_69)).

A few studies are on-going in Africa to test the application of *mHealth* to improve maternal health services for example the Mobile Technology for Community health (MoTeCh) in Ghana ([Grameen-Foundation 2011](#_ENREF_33)) and the *‘wired mother’* in Zanzibar, Tanzania ([ENRECA-Health 2010](#_ENREF_29)) whose results are still underway.

This planned intervention therefore intends to equip CHWs at the community level with hotline mobile phones that they can use to communicate directly with professional clinicians at the Primary care level.

Clinicians will also use it to communicate health relevant information to CHWs and help them make decisions on the correct course of actions for pregnant and lactating women within the community.

# Chapter Three

# 3.0 Problem Statement, Study Justification, Conceptual framework and Objectives

## 3.1 Problem Statement

Inconsistencies in the utilisation of antenatal services among pregnant women, to a large extent, contribute to the low uptake of institutional deliveries and hence high maternal and neonatal mortality rates ([Mpembeni, Killewo J et al. 2007](#_ENREF_63); [WHO 2009](#_ENREF_112)). At the primary care level, antenatal care service provision is often biased towards provision of technical biomedical services yet health education and counselling interventions ought to be integral components of antenatal care ([WHO 2002a](#_ENREF_103); [WHO 2002b](#_ENREF_104)). Often, group health education based on generic health topics are given to ANC women; inconsiderate of the unique needs and demands of the individual pregnant woman. Health workers concentrate on recording a series of clinical observations concluded with filling in the ANC card. Examination findings and its implications for the mother, her pregnancy or/and the newborn are not interpreted and discussed ([Titaley C, Hunter C et al. 2010](#_ENREF_84)). This current approach leaves women attending ANC uninformed about the benefits of making return visits and subsequently institutional deliveries.

As such, less than 50% of women attending ANC in sub Saharan Africa receive information regarding danger signs, birth preparation or the importance of institutional deliveries ([Nikiema, Beninguisse et al. 2009](#_ENREF_67)).

Inadequate information among pregnant women contributes to low institutional deliveries and low post-natal visits, poor homecare practices and hence stagnated maternal and neonatal mortality ([Nikiema, Beninguisse et al. 2009](#_ENREF_67)).

In Uganda, up to 97% of pregnant women make first Antenatal Care attendance, only 40% complete four visits and a similar proportion utilising institutional deliveries with a dismal 17% returning for postnatal visits within six weeks post partum ([UDHS 2006](#_ENREF_85)). In Masindi and Kiryandongo district 30% make completed four ANC visits and only 23.5% come for institutional delivery ([DHO 2010](#_ENREF_28)). There is no specific data for sub Saharan Africa regarding a combined package of individual prenatal education and use of mobile phone reminders during ANC.

Indeed, a combined package of prenatal education offered by a team of CHWs at the primary care level and application of mobile phones in maternal and neonatal health has not been explored in low income countries.

## 3.2 Study Justification

Available evidence suggest that repeated health education and counselling during ANC visits has the potential to enhance timely return visits, institutional deliveries and reduce the delay in seeking for care hence to reduce maternal and newborn deaths ([Barzgar M, Sheikh M et al. 1997](#_ENREF_8); [Ochako R, Fotso J et al. 2011](#_ENREF_71); [Rahman M, Haque S et al. 2011](#_ENREF_76)). Moreover ANC attendance in Low-and Middle-income countries offers a natural facility-based contact which can be strategically leveraged to increase institutional delivery ([Yakoob M, Menezes E et al. 2009](#_ENREF_117)). At the moment, individual prenatal educational intervention is not practiced at primary healthcare facilities ([Nikiema, Beninguisse et al. 2009](#_ENREF_67)).

Evidence mainly from Asia suggest that CHWs providing maternal and neonatal promotional care at the community can lead to increased uptake and utilisation of neonatal services and reduction of neonatal mortality ([Uta L and Sanders D 2007](#_ENREF_89); [Bhutta Z, Zohra S et al. 2010](#_ENREF_12)). Studies seeking to explain the low institutional deliveries often find answers from the users’ perspective such as ‘patients’ satisfaction’ and perceived quality of care ([WHO 2005](#_ENREF_107)) moreover, knowledge about the providers’ perceptions in the literature is scant ([Nikiema B, Beninguisse J et al. 2009](#_ENREF_66); [Magoma M, Requejo J et al. 2010](#_ENREF_56)). Opportunities to optimize prenatal educational interventions for Uganda exist: 95% of pregnant women make ANC visits at least once; 75% of the population live within 5km radius from the nearest health facility; health worker density is 62% of approved positions; existing national policy on Community Health Workers’ (Village Health Teams in Uganda) and 85% of population have access to mobile telecommunication.

Educational interventions during ANC however require competences in communication, sufficient time dedicated for dialogue ([WHO 2002a](#_ENREF_103); [WHO 2002b](#_ENREF_104)) and training for the health caregiver on basic scientific knowledge, medical care and a good understanding of local issues and cultural beliefs ([Bhutta Z, Zohra S et al. 2010](#_ENREF_12); [Howe, Manu et al. 2011](#_ENREF_41)) since most of the decisions taken during pregnancy and child birth are socially mediated ([Yakoob M, Menezes E et al. 2009](#_ENREF_117)).

The proposed two formative studies will illuminate on the implementation challenges of prenatal educational interventions at the primary care facility, elaborate on the common homecare practices and form a basis for design and implementation of prenatal educational interventions offered to pregnant women at the primary care facility.

Specifically, findings and experiences from these studies will provide a basis for districts and the ministry of health to better organise and offer the comprehensive prenatal care at the primary care facilities in Uganda and broadly contribute to the reduction of maternal and neonatal mortality.

## 3.3 Conceptual frame and study Objectives

*Figure 2:*  Conceptual frame relating health workers, CHWs and community

[Constructed from ([WHO 2002b](#_ENREF_104); [WHO 2010](#_ENREF_113); [Pattinson R, Kerber K et al. 2011](#_ENREF_74))]

**Impact**

**Outputs**

**Organisational inputs**

Interface between more technical ANC interventions and Educational intervention

Inter-face between Community and the formal HS

**Study one & two**

**Study one**

- Timely return ANC;
- Completed four ANC visits
- Institutional deliveries;
- Improved homecare practices

Pre- & neo- natal women

**Formal HS**

Educational & Counselling

Services by CHWs

**Formal HS**

Technical Services by nurses and midwives

Reduction in maternal & neonatal mortality

**Study three**

COMMUNITY

## 3.4 Conceptual framework

The conceptual framework suggests a distinction between the technical midwives and nurses on the one hand and health assistants and CHWs providing less technical interventions on the other. It illustrates a balance between the more technical and less technical ANC services with a special emphasis on providing individual health education to individual pregnant women attending ANC clinics.

It also defines the role for CHWs thus giving them visibility within the healthcare system while directly linking them to the community. This model recognises the complementary roles for both technical clinical services with promotional educational interventions.

## 3.5 Study Objectives

### 3.6 General objective

This study aims at measuring the effect of a combined package of individual ANC educational interventions and the use of mobile phones on uptake and utilisation of selected maternal and newborn healthcare services provided at the primary care facility as a strategy towards reduction of maternal and neonatal mortality.

### 3.7 Specific objectives

1. Assess the knowledge and perception of primary healthcare workers in Masindi and Kiryandongo regarding the provision of educational interventions to pregnant women attending ANC clinics
2. Determine the common prenatal and newborn homecare practices among communities of Masindi and Kiryandongo
3. Measure the effect of a combined package of prenatal educational intervention and use of mobile phone reminders on timely return visits, completed four ANC attendance, institutional deliveries and newborn care practices among pregnant women attending ANC clinics in Masindi and Kiryandongo districts

# Chapter Four

# Study Methodology

## 4.1 Study site and population

The study will be done in the two districts of Masindi and Kiryandongo in western Uganda, 214 kilometres northwest of Kampala the capital city of Uganda. It is bordered by Amuru district in the north; Oyam to the northwest; Apac in the South; Hoima to the southwest and Bulisa in the Northwest. It is administratively divided in to three counties (Buruli, Kibanda and Bujenje); eight sub counties and 40 parishes. The district population is projected to be 603,000 inhabitants by 2010, 50.9% being male and 49.1% female. Its inhabitants are of heterogeneous ethnicity and nationality; the Banyoro comprising about 59%. The population is predominantly rural with about 5.43% being urban. It has a population density of 124 per square kilometres.

There are two general Hospitals and one sub district hospital, Masindi, Kiryandongo and Bwijanga respectively; 16 health centre III levels and; 26 health centre II levels (Masindi having 25 health centres while Kiryandongo has 17. In this region ANC new attendance is 89.4%; ANC fourth visit is 29.4%; IPT_2_ at 27.2% and Health facility deliveries in this region is 23.5% ([DHO 2010](#_ENREF_28)).

# 4.2 Sub-study one

**Assessing the knowledge and perception of primary health care workers in the provision of maternal and newborn healthcare: implications for the optimisation of individual prenatal educational interventions offered to pregnant women**

## 4.2.1 Introduction

Individual prenatal education offered to pregnant women is an integral component of the comprehensive maternal and newborn healthcare package and widely accepted as a sustainable model for the delivery of maternal and newborn health services ([WHO 2010](#_ENREF_113)). Conceptually, an integrated approach for maternal and newborn health has a high potential for reduction in maternal and neonatal mortality ([UNICEF 2004](#_ENREF_86); [WHO 2004](#_ENREF_106)). In this model of care a primary healthcare worker provides a series of curative, preventive and promotional interventions to a pregnant woman during ANC, through the intra-partum at delivery to the postnatal period ([WHO 2003](#_ENREF_105)).

The comprehensive package can be categorised as ‘basic care’ for normal pregnancy and ‘normal care’ for a normal newborn or ‘special care’ for a sick newborn provided at two separate physical levels: health facility and at the household ([WHO 1994](#_ENREF_101)). Crucial to this approach are: proficiency, on the part of the care provider, in curative care early problem identification, prompt and appropriate intervention in case of illness as well as interpersonal communication skills and above all sufficient time to provide quality information and counselling ([WHO 2003](#_ENREF_105)).

In practice, health workers have demonstrated less confidence and interest in providing preventive and promotional care ([Leif E, Nguyen T et al. 2009](#_ENREF_53); [Waiswa P 2010](#_ENREF_96)) like advice for premature deliveries, warmth, feeding and basic hygiene practices for the newborn ([Waiswa P 2010](#_ENREF_96)). Some health workers do not give the needed time because of urgent and competing curative services ([Haines, Sanders et al. 2007](#_ENREF_35)). Others, emanating from their biomedical trainings, perceive promotional and preventive health services like educational interventions to be restrictive ([Roberfroid D, Lefevre P et al. 2005](#_ENREF_78)) and therefore not a domain for health care professionals ([Haines, Sanders et al. 2007](#_ENREF_35)).

However, data on health worker perception towards the comprehensive care model is scant in the literature ([Howe, Manu et al. 2011](#_ENREF_41)).This study precisely aims to explore the knowledge and perceptions of primary healthcare workers regarding individual prenatal interventions in the context of the comprehensive ANC care model for maternal and newborn health; understand the factors considered important by primary healthcare workers for the poor implementation of the comprehensive ANC model especially the individual prenatal educational component of care; their frustrations with this model and seek their opinion towards alternative approaches like the engagement of Community Health Workers, that have been used in other settings, as a complementary approach to the formal health services.

## 4.2.2 Research Questions

1. What is the knowledge base of health workers regarding pre-intra and postnatal care
2. What is the perception of healthcare workers towards the provision of individual prenatal education to pregnant women

## 4.2.3 Objectives

### 4.2.4 General

To assess the knowledge and perception of primary healthcare workers regarding prenatal educational interventions offered during ANC care; understand the factors contributing to poor implementation of the comprehensive care model with the aim of designing an intervention that will engage a team of CHWs to offer individual prenatal educational interventions to pregnant women.

### 4.2.5 Specific

1. Determine the knowledge of Primary Healthcare workers regarding preventive and promotional interventions offered to pregnant women and newborn babies during the pre-and neo-natal period
2. Assess the perception of primary healthcare workers regarding CHWs offering a combined package of prenatal educational interventions and using mobile phone reminders to pregnant women during ANC clinics
3. Explore the modality of engaging a team of Community Health Workers to offer prenatal educational interventions to pregnant women at the primary care facility

# 4.2.6 Methodology

This study will be done in Masindi and Kiryandongo districts. The study design will be a descriptive exploratory cross-sectional study design employing qualitative and quantitative data collection techniques. Respondents will be health managers and health workers routinely involved in offering prenatal care to pregnant women and newborns at the primary care level (Health Centres II, III, IV and ANC clinics of the hospitals) in the districts of Masindi and Kiryandongo. The study unit of analysis will be individual health workers. In the quantitative survey, we will administer questionnaires with Multiple-Choice-Questions adapted from ([Bhutta, Darmstadt et al. 2005](#_ENREF_13); [Leif E, Nguyen T et al. 2009](#_ENREF_53)) to a total of 132 nursing assistants, nurses and midwives who are routinely engaged in providing ANC and newborn care services at the primary care facilities in the two districts of Masindi and Kiryandongo. This questionnaire makes assessment for four major aspects of maternal and newborn care: Antenatal, intra-natal, postnatal and follow-up visits in the community. These questionnaires will be administered by the PI to all eligible health.

For the qualitative technique we will conduct up to 20 Key-Informant Interviews with stakeholders at the health facility using a pre-designed interview guide (*details of guide in annex 3*). Five health managers from each district will be recruited for the interview. These respondents will be purposively selected given their specific role as health managers (*list of respondents see annex 4*)

## 4.2.7 Sample size

Preliminary assessments indicate that the staffing level for nurses, midwives and nurse assistants in the two districts could stands at about 200 staffs. The sample size calculation will be estimated using the formulae for cross-sectional studies [$Z^{2}$PQ]/$\delta^{2}$]; substituting the formula-assuming a health worker knowledge of **50%**, with a sampling error of **9%** an estimated sample size of 120 health workers will be recruited. Considering a 10% non response we obtain a sample size of 132 health workers. All midwives working in the two districts, all nurses and nursing assistant in health centres II, III and IV and nurses working in the outpatients and children’s wards will be eligible to participate in the study. A total of 132 health workers from both districts will be randomly selected. A list of all health workers providing services in the two districts will be obtained from their respective health sub districts and stratified by the different cadres (Nursing assistants, nurses and midwives) and a proportionate number will be sampled. For both the quantitative and qualitative studies health workers will be visited in their respective health centres and data will be collected by the Principal Investigator.

## 4.2.8 Study variables

- Perceptions of health workers about comprehensive maternal and newborn care
  - Provision of technical ANC services for example taking and recording BP, weight, screening for HIV integrated with,
  - Provision of less technical and more promotional services mainly health education
- Perception of health workers about working with Community Health Workers
- Knowledge of health workers about pre-natal care
  - Dietary requirement for pregnant women
  - Danger signs during pregnancy
  - Birth preparation
- Knowledge of health workers about neo-natal care
  - Hygiene and cord care
  - Warmth for the newborn
  - Early initiation and exclusive breast feeding
  - Recognition of danger signs

## 4.2.9 Data collection

The PI will administer both qualitative and quantitative questionnaires to eligible health workers.

A double data entry will be made in to epidata version 3.02 computer program, checked, cleaned and exported to stata version 10.0 for analysis.

Data will be transcribed verbatim and analysed for major themes and emerging sub themes using NVivo computer software program. All data collection tools will be pretested twice for suitability and necessary adjustments done prior to field interventions. Written informed consent will be obtained from all participants in the study.

## 4.2.10 Data analysis plan

Quantitative data will be analysed and presented as descriptive statistics in terms of proportions of health workers who will obtain a minimum acceptable score; a second part will stratify scores according to the different health cadres for comparison purposes. We will capture the unique health workers’ perspectives towards provision of individual educational interventions for prenatal women by the health workers on the one hand and by CHWs on the other. Meanwhile for the qualitative data contents will be analysed and presented according emerging themes and these will be used to triangulate with the quantitative information gathered.

Results from this study will illuminate knowledge gaps and major implementation challenges with delivering educational and counselling interventions to prenatal women during ANC at the primary care facilities. It will further provide information on alternative ways of engaging community health workers in the provision of educational interventions at the primary care level. And finally it will suggest ways of utilising mobile phones to enhance return prenatal visits, increase institutional deliveries and improve newborn homecare practices.

### ****4.2.11 Sub study one-Summary of methodology****

| **Objectives** | **Methods** | **Indicator/data to be collected** | **Target population** | **Sample size** |
| --- | --- | --- | --- | --- |
| 1. **1.** Determine the knowledge of Primary Healthcare workers regarding preventive and promotional interventions offered to pregnant women and newborn babies during the pre-and neo-natal period | **Structured Interviews** | **Knowledge of health workers regarding pre-and neo-natal prevention and promotional intervention** | **Health workers routinely engaged in providing pre-and neo-natal health** | **Random sampling (132 health workers)** |
| **2.** Assess the perception of primary healthcare workers regarding the provision of individual prenatal educational interventions to pregnant women during ANC clinics   1. **3.** Explore the modality of engaging a team of Community Health Workers to offer individual prenatal educational interventions to pregnant women at the primary care facility | **Key-Informant Interviews** | **Perception of stakeholders** | **DHO, MS, PNO, manager for maternity unit of hospital, manager for Community health Department of hospitals, and manager for ANC clinic of hospital** | **Purposive sampling up to 20 stakeholders (health managers and health care providers) from Masindi and Kiryandongo** |

# 4.3 Sub-study two

**Prevalence of common pre-and neo-natal homecare practices their rationale and determinants among communities of Masindi and Kiryandongo districts in Western Uganda**

## 4.3.1 Introduction

Homecare givers for maternal and newborns very often engage in potentially harmful practices like applying substances on the cord, providing pre-lacteal feeds to the newborn and other unhygienic practices ([Ingunn M, Wamani H et al. 2007](#_ENREF_43); [Kesterton and Cleland 2009](#_ENREF_47); [Waiswa P, Stefan P et al. 2010](#_ENREF_99)). Moreover health seeking behaviours for maternal, child and newborn services in sub Saharan Africa remains very low and erratic ([WHO/UNICEF 2009](#_ENREF_115); [King R, Mann V et al. 2010](#_ENREF_49)).

Low uptake of services aggravated by delay in seeking for care significantly compromise maternal and neonatal survival outcomes leading to preventable deaths ([Bazzano A, Kirkwood B et al. 2008](#_ENREF_9)). Maternal and newborn care practices in the community are deeply rooted in tradition and myths surrounding pregnancy and neonates ([Damstadt G, Uzma U et al. 2006](#_ENREF_23); [Bazzano A, Kirkwood B et al. 2008](#_ENREF_9); [Kesterton and Cleland 2009](#_ENREF_47)).

Behaviour change and health seeking in relation to maternal and newborn health is pivotal in morbidity and mortality reduction strategies ([Bhutta, Darmstadt et al. 2005](#_ENREF_13); [Wade A, Osrin D et al. 2006](#_ENREF_95)). Successful implementation of educational intervention packages therefore requires in depth knowledge of prevalent pre-and neo-natal homecare practices within the local context and their influences on maternal and newborn care and care-seeking behaviour ([Bhutta, Darmstadt et al. 2005](#_ENREF_13); [Kesterton and Cleland 2009](#_ENREF_47)).

This study is specifically designed to explore and quantify common homecare practices, their rationale and determinants; analyse their harmful or beneficial characteristics and highlight areas for change or enhancement to inform the individual prenatal educational intervention strategies planned during ANC at the primary care level. While similar studies have been done elsewhere in Uganda ([Waiswa P, Peterson S et al. 2010](#_ENREF_98)), they site limitations related to the small and homogenous population in the Demographic and Health Surveillance Site (DHSS) where these studies were conducted. Our study will be conducted in Masindi and Kiryandongo with a diverse ethnic composition.

# 4.3.2 General objective

To understand the common pre-and neo-natal homecare practices; determine their magnitude, identify those potentially harmful and healthy practices in order to inform the future educational intervention offered to pregnant women at the primary care level.

## 4.3.3 Specific objective

1. Determine the common pre-and neo-natal homecare practices and their determinants among lactating women in Masindi and Kiryandongo districts
2. Investigate the rationale for the different homecare practices offered to pregnant women and newborn babies during the pre-,intra- and immediate post-natal period in the districts of Masindi and Kiryandongo
3. Explore homecare experiences received by lactating women during the pre-and neo-natal period in the district of Masindi and Kiryandongo

##

## 4.3.4 Methodology

This will be a cross-sectional population-based survey among lactating women, Traditional Birth Attendants and elderly caregivers. Data collection techniques will include Structured and In-Depth Interviews with lactating mothers, Key Informant Interviews with elderly care givers and Traditional Birth Attendants (TBAs).

The Key Informant Interviews with elderly care givers and TBAs will precede the structured interviews with lactating mothers so that new emerging themes can be incorporated in to the structured interview with lactating mothers. Elderly care givers and TBAs will be purposively identified based on their routine engagement in the provision of pre-and neo-natal care services to individual women in the communities.

### 4.3.5 Sample size and technique

The formulae for cluster surveys will be used to estimate the maximum number of clusters and sample size. Using the formula,

C= [Z^2^PQ] D/$\delta^{2}b]$ Where,

- Z, the standard normal deviate at 95% confidence which is 1.96
- P, the prevalence of homecare practices in the region taken to be 42% (0.42) [good cord care ([Waiswa P, Stefan P et al. 2010](#_ENREF_99))]
- Q, (1-p) is 0.58
- D, the design effect taken to be 2,
- $\delta$, a sample error of 5%
- b, the average number of individuals to be sampled per cluster = 40 (derived from 4.85% of expected deliveries per population),

Substituting, the number of clusters needed for the survey is 18.7, implying that a minimum sample size of 748; adjusted for 10% non response we will recruit 823 participants from 21 clusters (see annex 5).

### 4.3.6 Sampling procedure

For the quantitative survey, clusters will be stratified at the parish level; a list of all parishes will be obtained from the planning unit of the districts and 21 of them will be selected by a simple random technique using computer generated random numbers. At the parish level all lactating women with babies up to four months will be enumerated and a list generated. From this list a random sample proportionate to the total number will be identified and included in to the study. A written consent will be obtained from each participating lactating woman. Structured interview questionnaire (*see annex 6 for details*) will be administered by trained research assistants to all lactating women who provide consent to participate in the study.

This study will be conducted in Masindi and Kiryandongo districts in western Uganda. The questionnaire guide will be pre-tested twice to ensure suitability.

In the qualitative study, six elderly caregivers and six traditional birth attendants will be identified with the help of Local council I chairpersons or their representative from any of the 21 parishes sampled. Thirty lactating mothers stratified by age groups (adolescents 10-20 years and adults above 20 years) will be selected for In-Depth Interviews. All these will be individual interviews with open-ended questions. Written informed consent will be obtained from participants and the interview administered on acceptance (*see annex 7 for details of question guide)*. While we plan to interview a total of six elderly care givers and six TBAs the absolute number will be determined when we arrive at saturation point-when no more new practices and explanations are volunteered in subsequent interviews.

For the quantitative survey, lactating mothers having babies up to sixty days old (about two months) will be eligible for the study (see annex 5 for sampling procedure). The unit of analysis will be individual lactating mothers.

The number of lactating mothers to be interviewed per cluster will be allocated proportionate to the number of eligible lactating mothers available in the selected parishes.

Eligible lactating mothers will be identified by the use of village-bicycle scouts assisted by the local council I chairpersons (LC I) who will enumerate all eligible lactating mothers and generate a list; from this list a proportionate number from each of the parishes will be sampled using a simple random selection technique.

## 4.3.7 Study variables

- Maternal homecare practices
  - Nutritional requirements
  - Initiation of ANC attendance
  - Birth preparations
  - Exertion during pregnancy
- Neonatal home care practices
- Care during delivery
  - Care for the cord (cutting and cleaning)
  - Warmth for the newborn
  - Initiation of breast feeding
  - Provision of pre-lacteal feeds
- Harmful maternal and newborn practices
- Healthy maternal and newborn practices

## 4.3.8 Data collection and analysis plan

For both qualitative and quantitative data collection techniques research assistants familiar with the local dialect (Runyoro, Luo and Kiswahili) and possessing at least a degree in social sciences or public health having experience in social research will be recruited and trained for three days on the use of available tools and techniques. Pre-testing will be done twice to ensure suitability of the tools. Qualitative data from the Key Informant Interviews with elderly caregivers, TBAs and lactating women will be audio taped, transcribed verbatim and translated in to English, themes regarding prenatal and newborn homecare practices will be identified and described. A detailed description of the different care practices will be explored from elderly care givers and TBAs; these practices will be elaborated and broadly categorised according to prenatal, intra- and post-natal periods. The computer software program NVivo will be used. We will use content analysis to analyse data from TBAs and elderly caregivers and lactating mothers. Analysis will focus on making a detailed description of maternal and newborn care practices and the rationale for these care practices in the homes. For the In-Depth Interviews manual analysis of experiences of the different age groups adolescents (young adolescent ages 10-13 years; middle age 14-16 years and older adolescents ages 17-20) and older women over 20 years will be considered.

In the quantitative data with lactating mothers research assistants will be recruited and trained on the use of data collection tools; questionnaires will be pre-tested twice to ensue suitability of data collection tool; double data entry will be done in *epidata version 3.02*, cleaned and exported to *stata version 10.0* for analysis. Results will be presented in terms of descriptive statistics in proportions and rates. In a second level using bivariate data analysis, we will test for association between socio-demographic characteristics and the different maternal and newborn care practices using odds ratios and confidence intervals to test for levels of significance.

### ****4.3.9 Summary of objectives and methodologies for sub study two****

| **Objectives** | **Methods** | **Indicator/data to be collected** | **Target population** | **Sample size** |
| --- | --- | --- | --- | --- |
| 1. Determine the common pre-and neo-natal homecare practices and their determinants among lactating women in Masindi and Kiryandongo districts | **Key Informant Interviews** | **Common homecare practices** | **TBAs, Elderly caregivers** | **6 TBAs and 6 Elderly caregivers-3 each from Masindi & Kiryandongo** |
|  | **Structured Interviews** | **Different homecare practices received by lactating mothers during the pre-intra-and neonatal period** | **Lactating women** | **823 lactating mothers** |
| 1. Explore the rationale for homecare practices offered to pregnant women and newborn babies during the pre-,intra- and immediate post-natal period in the districts of Masindi and Kiryandongo | **Key Informant Interviews** | **Rationale/explanations for the common homecare practices offered to pregnant women and their newborn babies** | **TBAs. Elderly care givers** | **6 TBAs and 6 Elderly caregivers-3 each from Masindi & Kiryandongo** |
| 1. Document detailed experiences of care received by lactating women during the pre-and neo-natal period | **In-Depth Interviews** | **Detailed experiences of different age groups of lactating mothers regarding care offered to them during pre-intra-and immediate postnatal period** | **Sub groups of lactating women (adolescents 10-19 years and adults-20 and above)** | **Total: 30 lactating women-20 adolescents and 10 adult lactating women** |

# 4.4 Sub study three-the intervention

**Engaging Community Health Workers to provide a combined package of prenatal education interventions at primary care facilities with the use of mobile phone reminders from health workers and measure its effects on timely return visits, completion of four ANC visits, institutional deliveries and newborn care practices-a community intervention trial**

## 4.4.1 Introduction

The ultimate intervention will be based on data obtained from the two formative surveys and; based on our own knowledge about the context of Masindi and Kiryandongo and the subject of maternal and newborn care. The first study with health workers will inform the design of re-orienting health services at the antenatal clinic, while the community survey will form a basis for the appropriate educational messages to be delivered at the ANC clinics.

Primary outcomes of interest in this study are timely return ANC visits, completed four ANC visits, institutional delivery, and neonatal homecare practices. Secondary outcomes are post intervention community and health workers perceptions about the educational intervention offered by CHWs at the primary care facility.

The educational intervention will consist of **i)** a promotional team of community health workers supervised by a health assistant providing educational interventions at the primary care facility and; **ii)** the promotional team at the health centre using mobile phone reminders through Community Health Workers to pregnant women at the households.

## 4.4.2 General objective

Based on the findings from our initial studies regarding maternal and newborn health, we aim to: measure the effect of a combined package of CHWs offering educational interventions with use of mobile cell phone reminders on selected maternal and newborn indices.

**4.4.3 Specific**

1. Determine the effect of CHWs offering a combined package of prenatal education and using mobile phone reminders on timely return visits, completed four ANC visits, institutional deliveries and newborn care practices in Masindi and Kiryandongo districts
2. Explore post-intervention perception of lactating women towards the application of the combined package of prenatal educational intervention and mobile phone reminders

# 4.4.4 Methodology

### 4.4.5 Study design

We will conduct a community trial involving two arms: a community intervention trial.

1. One arm receives the routine ANC care available in the health facilities (standard care); this normally involves the provision of technical ANC services (history taking, palpation, PMTCT), group health education sometimes, and appointment for the next visit; rarely providing targeted or individualised health education
2. A second arm will receive in addition a *promotional team* comprising of a staff with a promotional profile (in this case health assistant) and three other CHWs providing educational interventions to augment the technical ANC intervention provided by qualified staffs. Health workers and CHWs will have in their possession mobile cell-phones. The mobile phones will be used by health workers to call CHWs to make reminders about return ANC visits for prenatal women, due dates for institutional deliveries, as well as providing additional educational information to pregnant women through the CHWs and any other consultations the CHW may want to make with health workers.

*Study population:*

The population to be studied will be pregnant women in Masindi and Kiryandongo districts who enrol for antenatal care in the 16 selected health centres for the two trial arms, with each trial arm utilising 8 health centres

### 4.4.6 Inclusion and exclusion criteria:

*Inclusion:*

All pregnant women found to be up to 28 weeks of pregnancy in Masindi and Kiryandongo districts registered for ANC in the 16 selected health centres will be eligible participants. Gestational age will be estimated using the Last Normal Menstrual Period (LNMP) as volunteered by the pregnant woman and verified by palpation of height of fundus.

Pregnant women sampled for inclusion in to the study will be followed until one month after the termination of pregnancy and they will be assessed for the primary outcomes of interest: **i)**  *timely return ANC visits* **ii)** *completed four ANC visits*, **ii)** *institutional deliveries* and **iii)** *homecare practices for newborns*. The end point for the follow-up of study participants will be up to four weeks after termination of pregnancy (the neonatal period).

Although early initiation of ANC in the first trimester is known to allow sufficient time for delivering repeated educational messages, the gestation age of 28 weeks has been purposely chosen because a significant proportion of women (about 45%) in this region of Masindi and Kiryandongo make their first ANC visit in the second trimester ([DHO 2010](#_ENREF_28)).

## Exclusion criteria:

i) Pregnant women above 28 weeks attending ANC in the 16 selected health centres of Masindi and Kiryandongo will be excluded

(ii) All pregnant women in Masindi and Kiryandongo who will not have enrolled for Antenatal Care visits in the targeted health centres by the time the two trail arms will be conducted.

(iii) Pregnant women in Masindi and Kiryandongo districts who would have enrolled for ANC in the selected health centres but found to be bed ridden or mentally disturbed.

All pregnant women recruited in to the study will be requested to provide two or three possible phone contacts through which they can be reached. These telephone contacts will be utilised during the follow-up stage in case the women do not make return visits. All health workers providing ANC care at the primary care facility will receive training in counselling. A standard operating procedure for the educational intervention at the ANC clinic to pregnant women will be developed (see annex 7.13), these messages will be adapted to suit the individual context and incorporate findings from formative surveys.

During ANC visits, qualified staff will first provide the more technical care to pregnant women and record all details on the patients card; this information will further be utilised by the team comprising of HA and CHWs to provide the targeted educational intervention; the promotional team will also engage the pregnant women to discuss in to details recommended aspects of care during pregnancy and newborn care like diet, birth preparation, danger signs in pregnancy, the advantages of delivering in the health facility and birth plans, care for the newborn including hygiene, breastfeeding care for the cord and warmth for the newborn.

### 4.4.7 Study variables

## Independent/factor:

(i) Routine ANC care available in the health facilities (standard care);

(ii) *Promotional team* giving additional education care to pregnant women with periodic mobile phone reminders.

*Dependent/outcome variables*

1. Timely return ANC visits (return within seven days of the scheduled appointment date)
2. Completion of the four recommended ANC visits
3. Institutional deliveries
4. Newborn care practices:
5. Provision of warmth to the newborn
6. Initiation of breastfeeding within one hour of birth
7. Care for the cord
8. Immunization for the newborn-Polio 0 & 1; BCG within the neonatal period

We will, at the end of the study, assess perceptions of individual women who have participated in this intervention towards the integrated package by administering the tool described by Howie and others ([Howie J, Heaney D et al. 1998](#_ENREF_42)) elaborated in annex 15 (the Patient-Enablement Initiative (PEI).

### 4.4.8 Sample size

Using the formula for cluster randomised trials suggested by Hayes and Bennett ([Hayes and Bennett 1999](#_ENREF_36)),

**n = D(Z_α/2_ + Z_β_)^2^[π_0_(1- π_0_) + π_1_(1- π_1_)]/ (π_0 +_  π_1_)^2^** where

- **n** = number of participants in each arm,
- **D** = the design effect of 2.5,
- **Z_α/2_** = 1.96,
- **Z_β_ = 0.84,**
- **π_0_ =** current rate of institutional deliveries, 23.5%
- **π_1_** = estimated rate of institutional deliveries after the intervention, 33.5%

Substituting in the formula, we obtain a sample size of 788; allowing for a 10% loss-to-follow up we estimate a total population of 868 participants per arm. Further substituting in the formula for required number of cluster, C

**C=1+(Z_α/2_ + Z_β_)^2^[π_0_(1- π_0_)/n + π_1_(1- π_1_)/n + k^2^(π_0_^2^ _+_ π_1_^2^)]/ (π_0 +_ π_1_)^2^**

Substituting for n=868, we arrive at a cluster number of 8 per arm; two arms will therefore require 1,736 participants distributed between 16 health centres; implying an average of 108 pregnant women enrolled per health centre for the entire four months. This will necessitate recruiting an average of 27 pregnant women per health centre per month.

This sample is based on the following assumptions:

- A degree of error, 0.05
- (95% confidence interval, **Z_a_** =1.96),
- the power to detect a significant difference between two comparison groups of 80% (**Z_a/2_** =0.84) to detect a 10% difference;
- **π_0_**, the current prevalence of institutional deliveries 23.5% ([DHO 2010](#_ENREF_28));
- **π_1_**, expected prevalence of institutional deliveries after the intervention 33.5% and
- A design effect **D,** 2.5 and
- Coefficient of variation between clusters of the same group **k_m_,** 0.25

### 4.4.9 Sampling procedure

Sixteen health centres in total will be proportionally selected from both Masindi and Kiryandongo district with Masindi contributing to 10 and Kiryandongo contributing to 6 HCs. A community trail involving two arms will be tried.

*Selection of the experimental units (pregnant women)*

Ten and six health centres will be proportionally selected from Masindi and Kiryandongo district giving a total of 16 heath centres in the trail.

From the 16 selected health centres, 8 of these will be purposively assigned to each of the two arms to minimize contamination. In each of the 8 health centres on each trial arm, 868 pregnant women with 28 weeks of pregnancy or below will be enrolled as they come in giving a total of 1736 in this trial. Health centres on two extremes of the districts will be assigned to either the intervention or the control group in order to avoid contamination between the two.

*Data Collection tools and procedures*

Semi-structured questionnaires written in English but administered in Runyoro will be used by research assistants.

*Training of research assistants*

Data collectors will be recruited and trained on data collection methods for two days. Training will include how to conduct individual interviews and data collection skills

**4.4.10 Data management and analysis**

At the end of each trail day, data will be collected, sorted and checked for consistency and for completeness. Open-ended questions will be categorized. All data collected will be entered in Epi-data soft ware package and exported to stata 8.2 soft ware package for more analysis. This analysis will be done in stages: univariate analysis ,where frequencies of variables will be generated and tabulated to establish the extent of use of these ANC services in the standard health centre setting compared to the second arm where extra education, and telephone remainders will be made. This level will also show the factors influencing timely return ANC visits, completion of the four recommended ANC visits, institutional deliveries and newborn care practices (Provision of warmth to the newborn, Initiation of breastfeeding within one hour of birth, Care for the cord and Immunization for the newborn-Polio 0 & 1; BCG within the neonatal period). At bi-variate level of analysis, cross tabulations will be run to establish associations between variables of interest. Chi-square tests will be used to establish significant associations. At multi variate analysis, all variables that will be found to be statistically significant at bi-variate ,will be subjected to the multivariate model (logistic) to control for confounding. The analysis will be run to get Odds Ratio values, 95%CI for the OR, and the respective p-values will be presented. Finally the primary outcomes of interest will be compared between the two arms i.e. a standard arm and the new trial arm upon which conclusions will be drawn.

*Quality Control*

To control quality of data to be collected,(completeness, correctness and consistency), the following measures will be undertaken:

Research assistants will be selected and trained by the principal investigator, questionnaires will be pre-tested for one day in a few selected health centres in Masindi and Kiryandongo. Completed questionnaires will be collected and checked by the principal investigator. Data collection will be carried out under strict supervision of the principal investigator. Data editing will be done and corrections will be made there at the selected health centres.

### 4.4.11 Study profile: sub study three

Figure 6

Masindi District

Kiryandongo District

Districts

HCs

16 Health Centres (VI, III & II)

Control Arm:

8 HCs (n=**868**):

Intervention Arm

8 HCs (n=**868**):

Study Arms

Recruitment & follow-up

**4 months**

Primary Outcomes

Outcome measures among study participants:

1. Timely ANC return visits
2. Completed 4 visits
3. Institutional deliveries
4. Homecare newborn care practices
5. Immunisation status of the newborn

868 Participants per arm Total = **1,736**

Follow-up

**10 months**

# 5.0 Ethical issues and considerations

Clearance will be obtained from the Institutional Review Board of MakSPH and the National Council of Science and Technology in Uganda. Authorisation to perform this research in the two districts will be obtained from both the national Ministry of Health and the district authorities and health managers. Details and benefits of this survey will be presented to the district authorities in a half-day seminar. Informed written consent will be obtained from the health workers and other participating community members (lactating women, TBAs, elderly care givers). Assent will be obtained from senior members of a household in case participants are less than 18 years. Confidentiality will be maintained personal identifications will be stored and only accessed by the study team.

Details of the study will be explained to all participants and they will be informed to feel free to participate or not in the study with no undesirable consequences to them.

# 5.1 Study limitations

1. This study focuses on prenatal mothers attending ANC services at the primary healthcare centres; systematically excluding the 5-10% of pregnant women who may not make any prenatal contact with the health facilities during the current pregnancy.
2. Some pregnant women make first ANC visit late in the third trimester, the primary target for this study are pregnant women reporting with gestation age of up to 28 weeks. For limitations one and two different intervention strategies are required to target this category of pregnant women who may have their peculiar needs different from the ones targeted by this study.
3. The risk of contamination across the study arms -this will be minimised by purposively allocating health centres in the extremes of the districts to either of the two arms.

# 5.2 References

Agho E, K., J. Dibley M, et al. (2011). "Determinants of exclusive breastfeeding in Nigeria." BMC: Pregnancy & Childbirth **11**(2).

Anonymous (2010). Global Consultation on Community Health Workers 29-30 April 2010-Montreux - Switzerland Concept Note. Global Health workforce alliance, World Health Organisation, Geneva.

Bagui A, H., K. Williams E, et al. (2007). "Newborn care in rural Uttar Pradesh." Indian Journal of Paediatrics **73**(3): 241-247.

Bahl, R., S. Qazi, et al. (2010). "Why Is Continuum of Care from Home to Health Facilities Essential to Improve Perinatal Survival?" Seminars in perinatology **34**(6): 477-485.

Bang A, T., A. Bang R, et al. (1999). "Effect of home-based neonatal care and management of sepsis on neonatal mortality: field trial in rural India." Lancet **354**: 1955-1961.

Baqui A, H., Shams El-Arifeen, et al. (2008). "Eff ect of community-based newborn-care intervention package implemented through two service-delivery strategies in Sylhet district, Bangladesh: a cluster-randomised controlled trial." Lancet **371**: 1936-1944.

Barber, S., L, and P. J. Gertler (2008). "Empowering women to obtain high quality care: evidence from an evaluation of Mexico's conditional cash transfer program." Health Policy and Planning **24**: 18-25.

Barzgar M, A., R. Sheikh M, et al. (1997). "Female Health workers boost primary care." World Health Forum. **18**(2): 202-210.

Bazzano A, N., R. Kirkwood B, et al. (2008). "Beyond symptom recognition: care-seeking for ill newborns in rural Ghana." Tropical Medicine and International Health **13**: 183-189.

Bhutta Z, A., Sajid S, et al. (2011). "Improvement of perinatal and newborn care in rural Pakistan through community-based strategies: a cluster-randomised eff ectiveness trial." Lancet **377**: 402-412.

Bhutta Z, A. and L. Seema, Eds. (2005). Global Forum Update on research for health 2005. Health research to achieve the millennium development goals. Child Health: how can health research make a difference? , Pro-book London.

Bhutta Z, A., L. Zohra S, et al. (2010). "Global Experience of Community Health Workers for Delivery of Health Related Millenium Development Goals: A Systematic Review, Country Case Studies, and Recommendations for Integration into National Health Systems." World Health Organisation, Geneva **Global Health Work Force Alliance**.

Bhutta, Z., A,, G. L. Darmstadt, et al. (2005). "Community-based interventions for improving perinatal and neonatal health outcomes in developing countries: a review of the evidence." PEDIATRICS **115**(2 Suppl): 519-617.

Bhutta, Z., A,, Samana A, et al. (2008). "Alma-Ata: Rebirth and Revision 6: Interventions to address maternal, newborn, and child survival: what diff erence can integrated primary health care strategies make?" Lancet **372**: 972-989.

Byaruhanga R, N., J. Nsungwa-Sabiiti, et al. (2010). "Hurdles and opportunities for newborn care in rural Uganda " Midwifery: doi:10.1016/j.midw.2010.1002.1005

Carroli, G., C. Rooney, et al. (2001). "How effective is antenatal care in preventing maternal mortality and serious morbidity? An overview of the evidence." Paediatr Perinat Epidemiol **15 Suppl 1**: 1-42.

Carroli, G., J. Villar, et al. (2001). "WHO systematic review of randomised controlled trials of routine antenatal care." Lancet **357**(9268): 1565-1570.

Cellette, F., A. Wright, et al. (2010). "Can the deployment of community health workers for the delivery of HIV services represent an effective and sustainable response to health force shortages? Results of multicountry study." AIDS **24**(Suppl 1): S45-S57.

Choudhury, N. and S. M. Ahmed (2011). "Maternal care practices among the ultra poor households in rural Bangladesh: a qualitative exploratory study." BMC Pregnancy Childbirth **11**: 15.

Clarke, M., Judy D, et al. (2008). "Community health workers in South Africa: Where in this maze do we find ourselves?" SAMJ **98**(9): 680-681.

Costello, A., V. Filippi, et al. (2007). "Research challenges to improve maternal and child survival." Lancet **369**: 1240-1243.

Daly, P. and F. Saadah (1999). "Indonesia: Facing the challenge to reduce Maternal Mortality." Watching Brief: East Asia and the Pacific Region(3): 1-6.

Damstadt G, L., Uzma U, et al. (2006). "Review of domiciliary newborn-care practices in Bangladesh." Journal of Health Population and Nutrition **24**(4): 380-393.

Darmstadt G, L., H. Mohamed H, et al. (2008). "Practices of rural Egyptian birth attendants during the antenatal, intranatal and the early neonatal periods." Journal of Health Population and Nutrition **26**(1): 36-45.

De-Brouwere V, Fabienne R, et al. (2010). "Access to maternal and perinatal health services: lessons from successful and less successful examples of improving access to safe delivery and care of the newborn." Tropical Medicine and International Health **doi:10.1111/j.1356.2010.02558.x**.

de Graft-Johnson, J., K. Kerber, et al. (2006). "The Maternal, Newborn and Child Health Continuum of Care." (Opportunities for Africa's newborns).

Dharma S, M., Osrin D, et al. (2004). "Effect of a participatory intervention with women’s groups on birth outcomes in Nepal: cluster-randomised controlled trial." Lancet **364**: 970-979.

DHO (2010). "Masindi district annual plan and budget." (Masindi district local government).

ENRECA-Health (2010). "Wired mothers - use of mobile phones to improve maternal and neonatal health in Zanzibar." Available at: <http://www.enrecahealth.dk/archive/wiredmothers/(Accessed:> 07-05-2011).

Frankel, S., Ed. (1992). The Community Health Worker: Effective programmes for Developing countries. Overview, Oxford Medical Publication.

Freeman P, B. Perry H, et al. (2009). "Accelerating progress in achieving the millennium development goal for children through community-based approaches'." Global Public Health,, First published on: 03 November 2009 (iFirst) **DOI: 10.1080/17441690903330305**.

Glenton C, B. Scheel I, et al. (2010). "The female community health volunteer programme in Nepal: Decision makers' perceptions of volunteerism, payment and other incentives." Social Science & Medicine **doi:10.1016/j.socscimed.2010.02.034**.

Grameen-Foundation (2011). "Empowering the poor: Information and Communication Technology (ICT) Innovation." Accessible at: <http://www.grameenfoundation.org/what-we-do/empowering-poor(date> accessed 07-05-2011).

Haines, A., D. Sanders, et al. (2007). "Achieving child survival goals: potential contribution of community health workers." Lancet **369**(9579): 2121-2131.

Haines, A., D. Sanders, et al. (2007). "Achieving child survival goals: potential contribution of community health workers." Lancet **Published online March 6, 2007 DOI:10.1016/S0140-6736(07)60325-0**.

Hayes, R. J. and S. Bennett (1999). "Simple sample size calculation for cluster-randomized trials." Int J Epidemiol **28**(2): 319-326.

Heggenhougen, K., H, and F. Magari, M., Eds. (1992). The Community Health Worker: Effective Program for developing countries. Community Health Workers in Tanzania, Oxford Medical Publisher.

Hill, Z., A. Manu, et al. (2008). "How did formative research inform the development of a home-based neonatal care intervention in rural Ghana?" J Perinatol **28 Suppl 2**: S38-45.

Hoestermann C, F., L,, Ogbaselassie G, et al. (1996). "Maternal mortality in the main referral hospital in The Gambia, West Africa." Tropical Medicine and International Health **1**(5): 710-717.

Hogan M, C., Kyle J F, et al. (2010). "Maternal mortality for 181 countries, 1980–2008: a systematic analysis of progress towards Millennium Development Goal 5." Lancet **375**: 1609-1623.

Howe, L. D., A. Manu, et al. (2011). "Developing a community-based neonatal care intervention: a health facility assessment to inform intervention design." Paediatr Perinat Epidemiol **25**(2): 192-200.

Howie J, G., R,, J. Heaney D, et al. (1998). "A Comparison of a Patient Enablement Instrument (PEI) against two established satisfaction scales an outcome measure of primary care consultation." Family Practice **15**(2): 165-171.

Ingunn M, S., E,, Wamani H, et al. (2007). "Low adherence to exclusive breastfeeding in Eastern Uganda: A community-based cross-sectional study comparing dietary recall since birth with 24-hour recall." BMC:Paediatrics **7**(10).

Isobel, C. (2011). "Sezing the mobile health opportunity." http//blogs.cfr.org/coleman/2011/05/03/seizing-the-mobile-health-opportunity/ **accessed 07/05/2011**.

Jones G SR, Black RE, et al. (2003). "How Many Child Deaths Can We Prevent This Year?" Lancet **362**(9377): 65-71.

Kapp, C. (2008). "Joy Lawn: Saving Newborns babys lives." Lancet **372**: 1141.

Kesterton, A., J, and J. Cleland (2009). "Neonatal care in rural Karnataka: healthy and harmful practices, the potential for change." BMC: Pregnancy & Childbirth **9**(20): doi:10.1186/1471-2393-1189-1120.

*Kind-en-Gezin* (2009). "The Child in flander 2009." <http://www.kindengezin.be/img/child-in-flanders-2009.pdf(Accessed> 04-07-2011).

King R, Mann V, et al. (2010). "Knowledge and reported practices of men and women on maternal and child health in rural Guinea Bissau: a cross sectional survey." BMC: Public Health **10**(319).

Kirkwood B, R., Manu A, et al. (2010). "NEWHINTS cluster randomised trial to evaluate the impact on neonatal mortality in rural Ghana of routine home visits to provide a package of essential newborn care interventions in the third trimester of pregnancy and the first week of life: trial protocol." Trials **11**: 58.

Kober K and W. Van Damme (2006). Expert patients and AIDS care. A literature review on expert patient programmes in high-income countries, and an exploration of their relevance for HIV/AIDS care in low-income countries with severe human resource shortages. Berlin and Antwerp 2006.

Lawn J, E., Pyande M, et al. (2006). "Africa's newborns-counting them and making them count." (Opportunities for Africa's newborns).

Leif E, N. Nguyen T, et al. (2009). "Evidence-based practice in neonatal health: knowledge among primary health care staff in northern Viet Nam." BMC: Human Resources for Health **7**(36).

Lewin S, Munabi-Babigumira S, et al. (2010). "Lay health workers in primary and community health care for maternal and child health and the management of infectious diseases (Review)." The Cohrane Collaboration.

Lubega, M., X. Nsabagasani, et al. (2009). "Policy and Practice, lost in transition: Reasons for high drop-out from pre-antiretroviral care in resource-poor setting of eastern Uganda." Health Policy **95**(2): 153-158.

Magoma M, Requejo J, et al. (2010). "High ANC coverage and low skilled attendance in a rural Tanzanian district: a case for implementing a birth plan intervention." BMC: Pregnancy & Childbirth **10**(13).

mhealth-Alliance (2011). "Mobilising innovation for global health." [www.mhealthalliance.org/ourwork/technology](http://www.mhealthalliance.org/ourwork/technology) **accessed 07-05-2011**.

Mikey R, Glenn L, et al. (2008). "Community participation: lessons for maternal, newborn, and child health." The Lancet **372**: 962-971.

MOH (2009). "Situation Analysis Village Health Teams Uganda 2009." Government of Uganda, Ministry of Health Kampala, Uganda.

MOH (2010a). "Situation analysis of Newborn Health in Uganda: Current status and opportunities to improve care and survival." Government of Uganda, Ministry of Health Kampala, Uganda.

MOH (2010b). "HEALTH SECTOR STRATEGIC PLAN III 2010/11-2014/15

" Government of Uganda, Ministry of Health Kampala, Uganda.

MOH (2010c). "The second National Health Policy: promoting people's health to enhance socio-economic development." The Republic Uganda **Ministry of Health**.

Mpembeni, R. N., Z. Killewo J, et al. (2007). "Use pattern of maternal health services and determinants of skilled care during delivery in Southern Tanzania: implications for achievement of MDG-5 targets." BMC Pregnancy Childbirth **7**: 29.

Mutyaba S, T. and A. Mmiro F (2001). "Maternal morbidity during labor in Mulago hospital." International Journal of Gynecology & Obstetrics **75**(1): 79-80.

Newlands D, Yugbare-Belemsaga D, et al. (2008). "Assessing the costs and cost-effectiveness of a Skilled Care Initiative in rural Burkina Faso." Tropical Medicine and International Health **13**(Suppliment I): 61-67.

Nikiema B, Beninguisse J, et al. (2009). "Providing information on pregnancy complications during antenatal visits: unmet educational needs in sub Saharan Africa." Health Policy and Planning **24**: 367-376.

Nikiema, B., G. Beninguisse, et al. (2009). "Providing information on pregnancy complications during antenatal visits: unmet educational needs in sub-Saharan Africa." Health Policy Plan **24**(5): 367-376.

Nirmala N, Prasanta T, et al. (2010). "Improving Newborn Survival in Low-income Countries: Community-Based Approaches and Lessons from South Asia." PLoS Med **7**(4): e1000246.doi:1000210.1001371/journal.pmed.1000246.

Noordam A, M., M. Kuepper B, et al. (2011). "Improvement of Maternal Health Services through the use of mobile phones." Tropical Medicine and International Health.

Nyonator K, F., K. Awoonor J, W,, et al. (2005). "The Ghana Community-based Health Planning and Services Initiative for scaling up service delivery innovation." Health Policy and Planning **20**(1): 25-34.

Ochako R, C. Fotso J, et al. (2011). "Utilization of maternal health services among young women in Kenya: Insights from the Kenya Demographic and Health Survey, 2003." BMC: Pregnancy & Childbirth **11**(1).

Ornella L, Seipati Mothebesoane-Anoh, et al. (2006). "ANC: Opportunities for Africa's Newborns."

Oystein E, O. (2010). "The impact of global health initiatives on trust in health care provision under extreme resource scarcity: presenting an agenda for debate from a case study of emergency obstetric care in Northern Tanzania." Health Research Policy and Systems **8**(14): doi:10.1186/1478-4505-1188-1114.

Pattinson R, Kerber K, et al. (2011). "Stillbirths: how can health systems deliver for mothers and babies?" Lancet **377**(9777): 1610-1623.

Perry H, Freeman P, et al. (2009). How Effective Is Community-Based Primary Health Care in Improving the Health of Children?A Review of the Evidence. Summary Findings Report to the Expert Review Panel Community-Based Primary Health Care Working Group, International Health Section American Public Health Association.

Rahman M, E. Haque S, et al. (2011). "Noninstitutional Births and Newborn Care Practices Among Adolescent Mothers in Bangladesh." Journal of Obstetric, Gynecologic, & Neonatal Nursing **40**(3): 262-273.

Renay W, Ronsmans C, et al. (2003). "Labour complications remain the most important risk factors for perinatal mortality in rural Kenya." Bulletin of the World Health Organisation **81**(7).

Roberfroid D, Lefevre P, et al. (2005). "Perceptions of growth monitoring and promotion among an international panel of District Medical Officers " Journal of Health Population and Nutrition **23**(3): 207-214.

Ronsmans C and G. Wendy J (2006). "Maternal Mortality: who, where, where and why." Lancet **368**: 1189-1200.

Schellengberg J A, Victoria C G, et al. (2003). "Inequities among the very poor: healthcare for children in rural southern Tanzania." Lancet **361**: 561-566.

Shiffman J (2010). "Issue attention in global health: the case of newborn survival." Lancet **375**: 2045-2049.

Siddhartha G and S. Harshpal S (2010). "Home visits by community health workers to prevent neonatal deaths in developing countries: a systematic review." Bulletin of the World Health Organisation **88**: 658-666B.

Thea D and Qazi S (2008). "Neonatal mortality--4 million reasons for progress." Lancet **371**(9628): 1893-1895.

Titaley C, R., L. Hunter C, et al. (2010). "Why do some women still prefer traditional birth attendants and home delivery?: a qualitative study on delivery care services in West Java Province, Indonesia." BMC: Pregnancy & Childbirth **10**(43).

UDHS (2006). "Uganda Demographic and Health Survey." Uganda Bureau of Statistics, Kampala Uganda **Government of Uganda**.

UNICEF (2004). "What works for children in South East Asia, Newborn care: An overview." United Nations Childrens fund, Health and Nutrition office, Regional office for South East Asia.

UNICEF (2009). "The state of the World's children 2009." available on: <http://www.unicef.org/sowc09/docs/SOWC09-FullReport-EN.pdf> **Accessed 07-05-2011**.

UNO (2000). "55/2 United Nations Millennium Declaration." United Nations General Assembly fifty fith session.

Uta L and Sanders D (2007). "Community Health Workers: What do we know about them? The state of the evidence on programmes, activities, costs and impact on health outcomes of using community health workers." World Health Organisation, Geneva.

Vanlerberghe V, E. Toledo M, et al. (2009). "Community involvement in dengue vector control: cluster randomised trial." BMJ **338:b1959**(doi:10.1136/bmj.b1959).

Victora C, G., Craig E R, et al. (2010). "Global report on preterm birth and stillbirth (4 of 7): delivery of interventions." BMC: Pregnancy & Childbirth **10**(Supp 1): S 4.

Villar J, Carroli G, et al. (2001). "Patterns of routine antenatal care for low-risk pregnancy." Cochrane Database Syst Rev(4): CD000934.

Villar, J., H. Ba'aqeel, et al. (2001). "WHO antenatal care randomised trial for the evaluation of a new model of routine antenatal care." Lancet **357**(9268): 1551-1564.

Vishwajeet K, Aarti K, et al. (2010). "Behavior Change for Newborn Survival in Resource-Poor Community Settings: Bridging the Gap Between Evidence and Impact." Seminars in perinatology **34**(6): 446-461.

Wade A, Osrin D, et al. (2006). "Behaviour change in perinatal care practices among rural women exposed to a women's group intervention in Nepal." BMC: Pregnancy & Childbirth **6**(20): doi:10.1186/1471-2393-1186-1120.

Waiswa P (2010). Understanding Newborn Care in Uganda-Towards Future Interventions. Thesis for doctoral degree. Doctoral Thesis for doctoral degree (PhD), Makerere University, Karolinska Institutet.

Waiswa P, Kemigisa M, et al. (2008). "Acceptability of evidence-based neonatal care practices in rural Uganda - implications for programming." BMC Pregnancy Childbirth **8**: 21.

Waiswa P, Peterson S, et al. (2010). "Poor newborn care practices - a population based survey in eastern Uganda." BMC Pregnancy Childbirth **10**: 9.

Waiswa P, Stefan P, et al. (2010). "Poor newborn care practices - a population based survey in eastern Uganda." BMC: Pregnancy & Childbirth **10**(9).

Warren C, Daly P, et al. (2006). "Postnatal Care."

WHO (1994). "Essential Newborn care: Maternal and Newborn Health." World Health Organisation, Geneva **Report of a technical Working Group**(WHO/FRH/MSM/96_13).

WHO (1996). "Revised 1990 Estimates of Maternal Mortality. WHO and UNICEF." World Health Organisation, Geneva.(WHO/FRH/MSM/96.11).

WHO (2002a). "Standards for Maternal and neonatal care development." World Health Organisation, Geneva **Department of Making pregnancy safer**.

WHO (2002b). "WHO Antenatal care randomised trial: Manual for the implementation of the new model." World Health Organisation, Geneva **Department of Reproductive Health and research family and community health**.

WHO (2003). "Integrated management of pregnancy and childbirth: Managing newborn problems; a guide for doctors, nurses and midwives." Department of Reproductive Health and Research, World Health Organisation, Geneva.

WHO (2004). "Roadmap for accelerating the attainment of the MDGs related to maternal and newborn health in Africa." World Health Organisation: Afro Region, Geneva.

WHO (2005). "Making every mother and child count." The World Health Report 2005.

WHO (2006b). "Strategic Approach to Improving Maternal and Newborn Survival and Health. Ensuring skilled care for every birth." World Health Organisation, Geneva.

WHO (2007). "Maternal Mortality in 2005. Evidence developed by WHO, UNICEF, UNFPA and The World Bank." World Health Organisation, Geneva.

WHO (2008a). "Hidden deaths of the world's newborn babies." Bulletin of the World Health Organisation **86**(4): 250-251.

WHO (2008b). "World Health Report 2008. Primary Health Care-Now More Than Ever." World Health Organisation, Geneva.

WHO (2009). "World Health Report 2009." World Health Organisation, Geneva.

WHO (2010). "Working with Individuals, Families and Communities to improve Maternal and newborn health." Bulletin of the World Health Organisation, department of Making Pregnancy Safer.(WHO/MPS/09.04).

WHO, I. F. (2004). "Making pregnancy safer: the critical role of the skilled attendant. A joint statement by WHO, ICM and FIGO." World Health Organisation, Geneva Switzerland.

WHO/UNICEF (2009). "Home visits for the newborn child: strategy to improve survival." Bulletin of the World Health Organisation(WHO/FCH/CAH/09.02).

Xiaoning L, Hong Yan, et al. (2010). "The evaluation of “Safe Motherhood” program on maternal care utilization in rural western China: a difference in difference approach." BMC: Public Health **10**(566).

Yakoob M, Y., V. Menezes E, et al. (2009). "Reducing stillbirths: behavioural and nutritional interventions before and during pregnancy." BMC Pregnancy Childbirth **9 Suppl 1**: S3.

Zarocostas, J. (2009). "Rich Nations are not giving enough for development goals to be met, says UN." BMJ **339**(b:2765).

| **Activity** | **Status (Not started, Ongoing, Completed)** | **Start Date/Expected Start Date** | **Completion Date/Expected Completion Date** |
| --- | --- | --- | --- |
| Ethical clearance |  | February 2012 | March 2012 |
| Conduct Sub Study One and Two | Data Collection, Data entry and transcription & Data analysis | March 2012 | April 2012 |
| Data analysis, Manuscripts for Sub studies one & two |  | May 2012 | September 2012 |
| Sub Study Three | Not yet Started | Expected to begin July 2012 | To End Oct 2013 |
| Data analysis and manuscripts for Sub study three |  | Expected to begin December 2012 | To End January 2014 |
| Dissertation writing and submission of thesis |  | March 2014 | July 2014 |
| Defence |  |  | November 2014 |

## ****Annex**** ****1**** Knowledge and perception of PHC workers

Adapted from ([Leif E, Nguyen T et al. 2009](#_ENREF_53)) for sub study one

**Optimization of individual prenatal education offered to pregnant women in Masindi and Kiryandongo district, Western Uganda: A community Intervention trial**

**Cadre of health worker**

1. Nurse Assistant,
2. Nurse,
3. Midwife circle appropriately

**Which level of healthcare are you currently working in?**

1. Hospital
2. HC IV
3. HC III

Which department are you currently deployed in?

1. Maternity, **2.** ANC, **3**. FP
2. Children, **5.** Male, **6**. Female, **7**. OPD

**Prenatal care:**

1. **Is it important for a pregnant woman to attend Antenatal care clinics at the health facility?**
2. Yes,
3. No,
4. I have not opinion,
5. **When should a woman make the first antenatal care visit during pregnancy?**
6. Immediately she misses her menstrual period,
7. After she has missed her period for two consecutive months,
8. After three months, d) Any time she wishes,
9. I have no opinion
10. **Who do you think should offer Antenatal care services to pregnant women? (*circle as many as you think)***
11. A midwife,
12. A Nurse,
13. A medical Doctor,
14. TBA,
15. Relative,
16. I have no opinion
17. **How many times should a pregnant woman attend antenatal care during the entire period of the pregnancy?**
18. At least once,
19. At least two times,
20. At least three times,
21. At least four times,
22. Any number of times she wishes,
23. I have no opinion
24. **What are the recommended routine interventions during antenatal clinics? (*circle all possible answers)***
25. History taking,
26. Physical examinations,
27. Laboratory examinations,
28. Health education,
29. Assessment for referral,
30. I have no opinion,
31. Routine prophylaxis
32. **Do you think you should provide health education to a pregnant woman every time she visits the antenatal clinic?**
33. No, only once in the first visit,
34. No, only once in the last visit,
35. Yes only once during the entire pregnancy
36. Yes for each visit,
37. Not necessary to give health education at all,
38. I have no opinion
39. **What are some of the vital information during pregnancy that you can discuss with a pregnant woman? (*circle all those you think are appropriate*)**
40. Dangers and complications during pregnancy,
41. Birth preparation,
42. Caring for the newborn baby,
43. The advantages of delivering in the health facility,
44. I have no opinion

1. **When a pregnant woman makes her first visit to the ANC clinic what are some of the important danger signs related to pregnancy that you will explain to her? (*circle as many as you think)***
2. Swelling of the feet and face,
3. Excessive vomiting,
4. Increased appetite for food,
5. I have no opinion
6. **What are some of the parameters that you will routinely measure during all prenatal visits? (*circle as many as you think*)**
7. Weight,
8. Blood Pressure,
9. Height of fundus,
10. I have no opinion

**Breastfeeding**

1. **When is it most appropriate to initiate breastfeeding after birth?**

**a**) Within the first hour,

**b)** 1-6 h after birth,

**c)** 6-12 h,

**d)** More than 12 h after birth,

**e)** I have no opinion,

**f)** Other.

1. **If the mother experiences that she has not enough breast milk during the first few days after delivery, what advice do you give her?**

**a)** To give formula while waiting for milk production to start,

**b)** To breastfeed more frequently,

**c)** To give rice water, herbal fluids or honey water while waiting for milk production to start,

**d)** Advise the mother to ask someone else to breastfeed her baby: for example, a neighbour who has enough breast milk,

**e)** I have no opinion,

**f)** Other.

1. **For how long should a mother continue to exclusively breastfeed her child?**

**a)** 1 month,

**b**) 2 months,

**c)** 4 months,

**d)** 6 months,

**e)** More than 6 months,

**f)** I have no opinion,

**g)** Other.

1. **According to you, after how long should a mother stop breastfeeding her child completely?**

**a)** 6 months,

**b)** 12 months,

**c)** 18 months,

**d)** 24 months,

**e)** More than 2 years,

**f)** I have no opinion,

**g)** Other

**Immediate postnatal care**

1. **Shortly after birth a healthy newborn baby should be able to: cry loud, have a pink colour to the skin, breathe evenly and have a respiratory rate of 40 – 60 breaths per minute. How would you take care of a baby that does not have these signs shortly after birth? (*circle as many as you think)***

**a)** Dry baby with cloth,

**b)** Use bag and face mask to help baby with respiration,

**c)** Suction of nose and mouth if necessary,

**d)** Slapping baby,

**e)** Pour cold water over baby,

**f)** I have no opinion,

**g)** Other.

1. **What can be done to prevent newborn children from bleeding?**

**a)** Breastfeeding the child,

**b)** Not necessary to give any drugs,

**c)** Give Vitamin K,

**d)** Give Vitamin K_1_,

**e)** I have no opinion,

1. **Do you know what dose of Vitamin K_1_ to give to a newborn baby according to recommendations in the National Guidelines?**

**a)** 0.5 mg, **b)** 1 mg, **c)** 2 mg, **d)** 5 mg, **e)** 10 mg,

**f)** I have no opinion, **g)** Other.

**Infection management**

1. **A newborn child can get eye infections after delivery. Which of the following alternatives would you use to prevent this from occurring? (*circle as many as you think*)**

**a)** Do not apply anything,

**b)** Apply breast milk in the babies’ eyes,

**c)** Clean eyes with sterile water,

**d)** Apply eye drops (silver nitrate) after cleaning eyes,

**e)** I have no opinion,

**f)** Other.

1. **Taking care of the umbilical cord of a newborn after delivery is important. Which of the following alternatives would you consider as important? (*circle as many as you think*)**

**a)** Always clean your hands before touching the cord,

**b)** Cut the cord with a clean instrument (for example, a razor blade),

**c)** Use any sharp instrument for cutting the cord,

**d)** After cutting the cord, apply traditional herbs/medicines,

**e)** Always put a bandage around the cord,

**f)** I have no opinion,

**g)** Other.

1. **What approach would you use to handle an umbilical cord that has any of the following signs: bad smell, oozing blood, small rashes around the umbilical area? (*circle as many as you think)***

**a)** Leave to dry,

**b)** Clean with water and soap,

**c)** Clean with iodine solution,

**d)** Apply antibiotic powder,

**e)** Refer to hospital,

**f)** I have no opinion,

**g)** Other.

**Temperature/Low birth weight**

1. **What is, in your opinion, the best way to stabilise the temperature of a newborn baby? (*circle as many as you think*)**

**a)** Bathing the baby in water of appropriate temperature,

**b)** By putting on clothes and cover head,

**c)** Having the baby skin-to-skin with her/his mother,

**d)** Keep the baby in a room with a temperature of 28-30°C,

**e)** Have the baby close to heat (radiator, fire, etc.),

**f)** I have no opinion,

**g)** Other.

1. **What is the definition of low birth weight for newborn child?**

**a)** Weight is less than 3000 grams,

**b)** Weight is less than 2500 grams,

**c)** Weight is less than 1500 grams,

**d)** Weight is less than 1000 grams,

**e**) I have no opinion,

**f)** Other

1. **What alternatives are important when taking care of a low birth weight baby? (*circle as many as possible)***

**a)** Bath the baby often,

**b)** Start breastfeeding early and frequently,

**c)** Keep the child warm,

**d)** Prevent infection from developing,

**e)** I have no opinion,

**f)** Other.

**Home visits**

1. **Why is it important to conduct home visits to mothers and their newborn babies after delivery? (*circle as many as possible*)**

**a)** To ask and examine the mother about her physical and psychological status,

**b)** To ask the mother about the baby’s behaviour (feeding, stools and urination, umbilical cord condition, etc.),

**c)** Examine the baby regarding icterus, (itching) weight, skin, umbilical cord, temperature, etc,

**d)** I have no opinion, **e)** Other

1. **When is the optimal time for health care personal to conduct the first home visit to a mother and her baby after delivery?**

**a)** Not important with home visits,

**b)** During the first 3 days,

**c)** Between day 3 and day 7,

**d)** Between day 8 and day 14,

**e)** I have no opinion,

**f)** Other.

1. **Who should conduct a home visit to a mother and her newborn baby after delivery? (*circle as many as you think)***
2. No one,
3. A village health worker,
4. A nurse,
5. A midwife,
6. A doctor,
7. I have no opinion,
8. Other

**The end,**

Thank you for your time

## Annex 2 Interview guide for FGD for sub study one

***2.1 Prevention and Promotional activities during ANC***-what are the activities that you routinely conduct during antenatal care at the health facility; (here we probe for history taking, physical examinations, laboratory examinations, Ferrous, Albendazole prophylaxis, health education, assessment for referral,

**What vital health education messages** are provided at this stage of pregnancy in addition to other curative services like Tetanus vaccination, test for Syphilis and HIV; explore for any birth preparation messages (clothing, finances, the relative who will be assisting her during labour, in case of emergency delivery); approaches for clean delivery, use of sterile instruments, tying of the cord, application of substance to the cord, ensuring warmth for the baby, initiation of breast feeding, any specific messages regarding warmth, breast feeding and hygiene, pre-lacteal feeding; what advice is given to the mother about care for the mother and the new born baby (in terms of feeding, hygiene, warmth and care for the cord

***2.5 what are the main challenges you face with this process***-What are/is their frustration with the process of providing the comprehensive ANC package - curative as well as preventive and promotional healthcare services to pregnant mothers and newborn babies; explore for the workload, the time needed to provide the necessary information; the challenges with late arrivals of the mothers and the newborns

***2.6 What you think about VHT performing some of these functions***-What is their opinion about sharing the burden of work with VHTs; what can the VHTs do to bridge this gap; which aspects of care do they think CHWs can do better; which aspects of care are the PHC workers are more comfortable with; how can the two separate roles be harmonised;

***2.7 What relationship they advocate for***-What kind of relationship would they want to see between the formal healthcare system and the CHW/VHTs; what they think about separation of roles between PHC workers and the VHTs; would they encourage consultations from VHT members, or referrals from the VHT; how would they like these referral system to function

***2.8 Possible linkage with formal healthcare system***-how do they think linkages can be created; what activities can the formal healthcare system perform together with the VHTs; how can the functions of the VHTs be monitored; how can the VHTs be supported to function better; how can VHTs enhance the uptake and utilisation of the services organised at the formal health system to reduce on the delays at home

***2.9 How telecommunication can enhance this linkage***-Is there any role for telecommunications in enhancing the linkage between the formal healthcare system and the VHTs; can the telephone system be used for consultations with the first-line health services; is this likely to work; can mobile phones be used to provide reminders to pregnant women through the VHTs; what are the challenges this intervention is likely to encounter; are they (the health workers) ready to be consulted by phone

***3.0*** **Any other comments that you think are relevant to this discussion but I have not mentioned**

## Annex 3 interview guide for KIIs for sub study one

***2.1 Prevention and Promotional activities during ANC***-what are the activities that you routinely conduct during antenatal care at the health facility; (here we probe for history taking, physical examinations, laboratory examinations, Ferrous, Albendazole prophylaxis, health education, assessment for referral,

**What vital health education messages** are provided at this stage of pregnancy in addition to other curative services like Tetanus vaccination, test for Syphilis and HIV; explore for any birth preparation messages (clothing, finances, the relative who will be assisting her during labour, in case of emergency delivery); approaches for clean delivery, use of sterile instruments, tying of the cord, application of substance to the cord, ensuring warmth for the baby, initiation of breast feeding, any specific messages regarding warmth, breast feeding and hygiene, pre-lacteal feeding; what advice is given to the mother about care for the mother and the new born baby (in terms of feeding, hygiene, warmth and care for the cord

***2.5 Their frustration with this process***-What are/is their frustration with the process of providing the comprehensive ANC package - curative as well as preventive and promotional healthcare services to pregnant mothers and newborn babies; explore for the workload, the time needed to provide the necessary information; the challenges with late arrivals of the mothers and the newborns

***2.6 What they think about VHT performing some of these functions***-What is their opinion about sharing the burden of work with VHTs; what can the VHTs do to bridge this gap; which aspects of care do they think CHWs can do better; which aspects of care are the PHC workers are more comfortable with; how can the two separate roles be harmonised;

***2.7 What relationship they advocate for***-What kind of relationship would they want to see between the formal healthcare system and the CHW/VHTs; what they think about separation of roles between PHC workers and the VHTs; would they encourage consultations from VHT members, or referrals from the VHT; how would they like these referral system to function

***2.8 Possible linkage with formal healthcare system***-how do they think linkages can be created; what activities can the formal healthcare system perform together with the VHTs; how can the functions of the VHTs be monitored; how can the VHTs be supported to function better; how can VHTs enhance the uptake and utilisation of the services organised at the formal health system to reduce on the delays at home

***2.9 How telecommunication can enhance this linkage***-Is there any role for telecommunications in enhancing the linkage between the formal healthcare system and the VHTs; can the telephone system be used for consultations with the first-line health services; is this likely to work; can mobile phones be used to provide reminders to pregnant women through the VHTs; what are the challenges this intervention is likely to encounter; are they (the health workers) ready to be consulted by phone

## Annex 4 Focal persons for the Key-Informant Interviews for sub study one

| **S/N** | **Officer to be Interviewed** | **Number** |
| --- | --- | --- |
| 1 | District Health Officer | 2 |
| 2 | Medical Superintendent | 2 |
| 3 | Officer in charge of the Community Health Department | 2 |
| 4 | Principal Nursing Officer | 2 |
| 5 | Head of Unit Maternity | 2 |
| 6 | Head of Unit Antenatal Unit of hospital | 2 |
| 7 | Midwives Based at the health centres II, III, IV | 8 |
|  | **TOTAL** | **20** |

## Annex 5 Sampling procedure for sub study two

Districts Masindi & Kiryandongo

Parishes (**21**)

**3** Villages per Parish

Lactating women with babies up to two months or sixty days at the time of the study **(823)**; regardless of parity and where delivery took place for semi-structured interview

Purposively identify **6** TBAs from any of the parishes and **6** elderly home caregiver from any of the parishes selected for KII interview;

Identify **30** lactating mothers from each of the following age groups: 10-13 years; 14-16 yrs; 17-19 yrs and 20 years and above for In-Depth Interviews

## Annex 6: data collection guide for Structured Interview for lactating women sub study two

Data collection (adapted from ([Bagui A, Williams E et al. 2007](#_ENREF_3); [Bazzano A, Kirkwood B et al. 2008](#_ENREF_9); [Darmstadt G, Mohamed H et al. 2008](#_ENREF_24); [Kesterton and Cleland 2009](#_ENREF_47))

**Optimization of individual prenatal education offered to pregnant women in Masindi and Kiryandongo district, Western Uganda: A community Intervention trial**

Data collection tool for lactating women;

**A: Socio-demographic characteristics**

1. Code number..........................................................................................
2. Name of household Head........................................................................
3. Age of Respondent in completed years (lactating woman)....................
4. Parity of respondent 1, 2, 3, 4, 5, over 5
5. Highest education level attained:
6. Non
7. Primary;
8. Secondary;
9. High school;
10. Tertiary;
11. Marital status:
12. Married
13. Single
14. Separated
15. Divorced
16. Widowed
17. What do you do for a living:
18. Nothing
19. Housewife
20. Farming/gardening
21. Selling in the market/petty trade
22. Business woman
23. Salaried
24. Did you attend any ANC during the last pregnancy:
25. Yes, 2. No
26. Number of ANC visits made with the last pregnancy:
27. 0,
28. 1,
29. 2,
30. 3,
31. 4
32. More than 4
33. Where did this last delivery take place:

**1)** Home;

**2)** Health facility;

**3)** On the way to health Facility

1. How old is the baby now? Age in completed weeks
2. 0-1 week **2)** 2-4 weeks **3)** 5-8 weeks **4)** 9-13 weeks
3. Number of living children: 1, 2, 3, 4, 5, 6, more than 6
4. Number of deceased children: 0,1, 2, 3, 4, 5, 6, more than 6

(*if deceased babies is* ***0*** *then skip to section* ***B***)

1. At what ages did the deceased children die?

**a) First death,**

1) Died as a still birth

2) Within 24 hours

3) After 24 hours but within 72 hours

4) After 72 hours but within 7 days

5) After 7 days but within 28 day

6) After 28 days

**b) Second death,**

**1**) Died as a still birth

**2**) Within 24 hours

**3**) After 24 hours but within 72 hours

**4**) After 72 hours but within 7 days

**5**) After 7 days but within 28 day

**6**) After 28 Days

**c) Third death,**

**1**) Died as a still birth

**2**) Within 24 hours

**3**) After 24 hours but within 72 hours

**4**) After 72 hours but within 7 days

**5**) After 7 days but within 28 day

**6**) After 28 Days

13. Indicate where the deceased babies were were born from:

**1)** At home

**2)** At health facility

**3)** On the way to Health facility

**B: Data on the last pregnancy**

1. During your last pregnancy is there any food that was forbidden for you to eat?

1=Yes

2=No

1. If YES why?

1) will make the baby grow too big

2) will cause the baby to come out;

3) other, Specify...................

1. During this pregnancy were you advised not to take part in certain activities?

1=Yes

2=No

1. If YES, please *(circle all that apply)*:

1) Not going to the well to fetch water

2) Not using a pit latrine

3) Not engaging in sexual intercourse

4) Other, please Specify...................

1. Did you make any preparation for this delivery?

1=Yes

2=No

1. If YES, what specific preparations did you make? *(Circle all that apply)*

**1**) Secured warm dresses and other items for the baby like basin,

**2**) mama kit, mackintosh,

**3**) saved some money,

**4**) decided where to deliver from,

**5**) selected a caretaker for the remaining children while I am away ,

**6**) other, please specify

**C: Information on Delivery**

1. Mode of delivery of the current baby:

**1**) Spontaneous Vaginal delivery;

**2**) C/S

**3**) vacuum;

4) Forceps

1. Place of delivery:

**1)** health centre,

**2)** hospital

**3)** home

**4**) on the way to Health Facility

**5)** At the TBAs home

1. Who attended to you during delivery

**1**) By trained health worker

**2**) A close relative

**3**) By TBA

**4)** Unattended to

1. Timing of labour-in your view was this labour:

**1)** Normal

**2)** Longer than usual

**3)** Too long

**4)** Too short

**5)** No idea

1. Did you develop any intra-partum complication: *(Circle all that apply)*
2. too long labour
3. bleeding
4. swelling of the feet
5. convulsions
6. ruptured uterus
7. obstructed labour
8. non

**D. Clean delivery**

**T**

1 What instrument was used for cutting the cord?

1. New razorblade
2. Sterilised instrument
3. Used/old razor blade
4. A knife
5. Other sharp objects used in the household
6. What instrument was used to tie the cord?
7. Clean thread was used
8. Application of spirit/alcohol
9. Thread from household cloth
10. Pieces of used cloth
11. What substance was applied on the cord?
12. Application of powder on the stump
13. Application of animal waste
14. Application of soot powder
15. Salt water
16. Herbal medicines
17. Nothing

**E. Warmth**

1. At what stage after delivery was the newborn dried up

1. Immediately before the placenta was delivered
2. Immediately after delivery of the placenta
3. After the mother was cleaned and wrapped
4. After the whole process of the delivery was completed
5. Not dried, just wrapped

2 .At what stage was the newborn wrapped up in a warm cloth

1. Immediately before the placenta was delivered
2. Immediately after the placenta was delivered
3. Long after the delivery was competed

3. What was the timing of the first bath after delivery?

1. Immediately after delivery;
2. Not immediately but within 6 hours;
3. 7-23 hours after;
4. Second day after delivery or later

**F. Assessment of newborn**

1. Was there any need for Resuscitation of the newborn?

1=Yes

2=No?

2. If YES, what method was used for resuscitation?

1. Hanged the legs up,
2. Sprinkled cold water,
3. Mouth-to-mouth resuscitation
4. Slap the baby
5. Oxygen
6. Nothing

3. How was the weight of the newborn assessed?

1. Using a weighing scale
2. Estimation by looking at the size
3. Not done

4.1. If baby was premature how was assessment of prematurity done? (*If the response is NO then skip to section* ***G)***

1. Size of the baby
2. Told by health practitioner
3. Weight of the baby
4. Gestation age of the pregnancy
5. Anthropometric measurements

4.2. If Anthropometric measurements which one?

1. Feet
2. Arm
3. Head
4. thigh

5. Was there any special care given to this premature newborn baby?

1=YES

2=NO

6. If YES, can tick more than one option.

1. Extra warmth
2. Extra feeds
3. Frequent feeds
4. Less frequent bathing
5. No bathing at all
6. Keeping the baby indoor only
7. Specific traditional performances

**G. Care-seeking practices**

1. Did you make any postnatal visits to the health facility

1=Yes

2=No

1. How many postnatal visits so far have you made: 1,2,3,4
2. How early was the first visit
3. Within the first 24 hours after delivery
4. After 24 hours but before 72 hours
5. After 72 hours but before one week
6. After one week
7. After one month

**H. Care seeking**

Newborn immunisation

1. Is the newborn already immunised for

1. Polio zero 1=Yes 2=No
2. BCG 1=Yes 2=No
3. Polio 1 1=Yes 2=No
4. DPT HEB+Hib (pentavalent) 1=Yes 2=No

2.Did this newborn fall ill in the last three months? 1=Yes 2=No)

(*if NO conclude the interview*)

3. If yes how did you detect the illness? *(Circle all that apply)*

1. Failure to feed
2. Excessive crying
3. Less activity
4. Warm to touch

4.What immediate actions did you take? *(Circle all that apply)*

1. Attention to VHT
2. To Health centre
3. Seek medication at home
4. Traditional healers
5. Went to private clinic
6. Did nothing

5.How soon after the onset of illness did you seek for care?

1. Within one hour
2. Within six hours
3. Within 24 hours
4. After 24 hours
5. What in your view was the cause of illness?
6. Evil spirit
7. Some evil neighbours or relatives
8. Bad air
9. The infected umbilical stump
10. Acquired from place of delivery
11. Acquired from mother
12. No idea

Thank you for your time

## Annex 7: Key Informant Interview guide with elderly caregivers and traditional birth attendant for sub study two

1. *Introducing the subject*

You have been a Traditional Birth Attendant/care provider for many years now; and for sure you have had a long experience working with pregnant women; conducting deliveries and caring for newborn babies. Today, I want to learn how care is provided for a pregnant woman here at home: before delivery, during the labouring process and how delivery is conducted here in the home, how the cord is handled and the other activities that people normally perform until the baby is two months old. What traditional rituals if any, are performed along this process?

1. *Climate setting*

How many years have you worked as a traditional birth attendant? How did you become a traditional birth attendant? Do you enjoy doing the work of a traditional birth attendant? How many deliveries have you conducted this year alone? Who invites you to conduct a delivery in the home? Do the family members come to you here or you go to the home of the labouring woman? What happens if it is in the night?

1. *Antenatal period (prohibition from eating some foods or engaging in certain activities)*

Sometimes pregnant women are advised not to eat certain foods or perform certain tasks while pregnant, what are the foods that pregnant women are not allowed to eat here in your village? Why are they stopped from eating these types of foods-does it harm them or harm the baby? (If there is none probe for what she has heard that is prohibited and why)

1. *During labour-intra-partum care*

When a pregnant woman goes in to labour where is she made to labour and deliver from? In the home-in the house, under the tree or a separate shelter is built purposely for the delivery? If it is during the day or at night (you are exploring for cleanliness and warmth and any traditional explanations for the actions)

During the delivery of the baby and when the baby has come out, what happens next? Who cuts the cord? At what stage is the cord cut? What instruments are used for cutting the cord? Who provides the instruments for cutting the cord-(the family of the labouring woman yourself) And how is the bleeding from the cord of the baby stopped?

1. *Post-partum*

What about the placenta-who delivers the placenta out of the woman’s womb? Please explain to me how this is done to make it come out of the woman. (in case hesitant probe-Do you have to pull it out or you wait for it to come out by itself)

At this stage that the cord is not yet cut and the baby is out what do you do with the baby? Where do you place him/or her? After the cord has been cut what happens with the baby? After delivering the placenta what happens with the placenta? And after delivery of the placenta what next do you do for the mother.

After all these the cord of the baby remains fresh for some days with a wound at the stump. Does this worry you at home? What is done to make the wound to heal properly?

Have you had any experience when sometimes babies come out when they are very tired-when you are the one assisting the pregnant woman with delivery how will you know that a baby is tired? And when you notice that the baby is tired, what actions do you take for the baby?

The baby is expected to feed-at what time do you start to feed the baby? Usually there is no breast milk at this time-what do you do feed for the baby

What advice do you give to the mother or attendant before leaving her to go home and rest?

Sometimes the newborn will fall sick (before reaching even one month)-have you had an experience when a newborn is sick? How can you notice that a newborn could actually be sick? What do you do for the parents of the sick baby and what do you do for the sick baby? What do other people do for such a sick newborn?

## Annex 8: Interview guide for In-Depth Interviews with sub groups of lactating women

The information we are interested in are the dos/do not; the care and advise received and from whom this support is coming.

1. Tell me about the pregnancy of this baby that you are carrying-how was it like
2. What about the labour and delivery-what are your memories about this event in your life
3. Tell me about the immediate post-natal period-what kind of services/support/advises did you receive for yourself and for the baby?

## Annex 9: Sub study three Flow-diagram for educational intervention

**Arm 1**

Routine ANC & Newborn Services only

Absence of individualised prenatal health education; no CHW deployed; no mobile phones

**Primary outcomes**

1. Timely return visits
2. Completed Four ANC visits
3. Institutional Deliveries
4. Improved newborn care practices and newborn immunisation

**Outcomes**

Maternal mortality

Neonatal Mortality

**Arm 2**

Designated staff:

1. Health Assistant and
2. a team of 3 CHWs)

For educational intervention at the health facility

1. CHW using Mobile phones

Presence of individualised prenatal health education offered by CHWs; use of mobile phones to communicate vital information between CHWs and Health workers, provide reminders for return dates and delivery dates

## Annex 10: List of eligible Health Centres by HSD

| **S/N** | **HSD** | | |  |
| --- | --- | --- | --- | --- |
|  | **Buruli** | **Bujenje** | **Kibanda** |  |
| 1 | Kimengo | Budongo | Mutunda |  |
| 2 | Kilanyi | Ikoba | Diika |  |
| 3 | Pakanyi | Nyantonzi | Panyadoli |  |
| 4 | Nyakitibwa | Mihembero | Diima |  |
| 5 | Kyatiri | Ntooma | Karungi |  |
| 6 | Kigezi | Bwijanga | Kigumba |  |
| 7 | Kibwona | Kikingura | Katulikire |  |
| 8 | Kijunjubwa | Nyabyeya | Apodorwa |  |
| 9 | Kitanyaata | Kyamaiso | Mpuumwe |  |
| 10 | Kijenga | Kisalizi | Masindi port |  |
| 11 | Kibyamu |  |  |  |
| **Totals** | **11** | **10** | **10** | **31** |

# Consent form

## Annex 11: Consent form for Health workers and managers

**Introduction:** My Name is (........Interviewer), and I am part of the team conducting a study to assess the current situation of maternal and newborn care in Masindi and Kiryandongo districts.

**Purpose:** maternal and newborn health has been and remains a major health problem in Masindi and Kiryandongo and indeed Uganda as a whole. We know that this problem can be solved by knowing what goes on the health facilities as well as in the homes. However at this point we do not exactly know what the health workers/the community members think about maternal and newborn health. We also do know that conditions affecting pregnant women and newborns in this area are a grave matter and usually life-threatening, with important consequences to the families and communities. The aim of this study is therefore to assess the current knowledge and perceptions of health workers regarding maternal and newborn health services at the primary healthcare facilities.

**Research procedure:** we are unable to visit every individual health worker due to several other constraints and so will only take a representative sample of health workers in both Masindi and Kiryandongo districts. You have been selected to participate in this exercise. We will ask you specific questions regarding the routine care provided to pregnant women and newborns at the health facilities

**Benefits of this study:** this study will help us to understand the current provision of prenatal and newborn healthcare the challenges experienced by health workers and the reasons considered by health workers to be important in the provision of health care to pregnant women the newborn babies and the extent of knowledge among health workers regarding pregnant women and newborn babies

**Potential harm from this study:** there is no harm that we foresee by you participating in this study.

**Voluntary participation:** Participation in this study is voluntary. If you accept to take part then I will ask you specific questions; you are not obliged to answer any of the questions if you do not feel comfortable about it. You are free to withdraw from this interview at any point without any undesirable consequences to your career or welfare.

**Confidentiality:** we shall ensure confidentiality of the information that you provide; your names will not be written anywhere during this interview. All information you provide will be kept away and will only be accessed by the investigator for purposes of this study. Your decision to participate or not will not in any way jeopardise your career or relationships within the health system. If you have any questions please feel free to ask me

**Acceptance:** the interview will take about 30 minutes to answer all the questions. Before we proceed I would like to seek your permission to participate in this interview. Do you accept to participate in this interview for the survey?

(Circle) Yes or No

I have received a full explanation about this survey and understand its purpose and objectives. I understand the details and hereby agree to participate in the survey

Signature of respondent Date

Signature of interviewer Date

In case of any doubts please call Richard Mangwi on mobile number 0772 829 377 or John Ssempebwa on 0703 944404 the chairman of the Higher Degrees and Ethics Committee of the School of Public Health *Annex 12: Consent form for Elderly care givers and Traditional Birth Attendants*

**Introduction:** My Name is (........Interviewer), and I am part of the team conducting a study to assess the current situation of maternal and newborn care in Masindi and Kiryandongo districts.

**Purpose:** maternal and newborn health has been and remains a major health problem in Masindi and Kiryandongo and indeed Uganda as a whole. We know that this problem can be solved by knowing what goes on the health facilities as well as in the homes. However at this point we do not exactly know what the health workers/the community members think about maternal and newborn health. We also do know that conditions affecting pregnant women and newborns in this area are a grave matter and usually life-threatening, with important consequences to the families and communities. The aim of this study is therefore to assess the current knowledge and perceptions of health workers regarding maternal and newborn health services at the primary healthcare facilities.

**Research procedure:** we are unable to visit every individual Elderly care giver/Traditional Birth Attendant due to several other constraints and so will only take a representative sample for both Masindi and Kiryandongo districts. You have been selected to participate in this exercise. We will ask you specific questions regarding the care that you routinely provide to prenatal and newborns that you attend to; and the reasons for providing this care in the communities.

**Benefits of this study:** this study will help us to understand what kind of care is provided to prenatal and newborn babies in the homes and how care is provided to prenatal women and newborn babies in this community.

**Potential harm from this study:** there is no harm that we foresee by you participating in this study. These questions are not meant to pass any judgment towards the work that you are doing but rather to appreciate your role in providing care to prenatal and newborn babies. The information that you provide will therefore not be used to judge your practices as wrong or right

**Voluntary participation:** Participation in this study is voluntary. If you accept to take part then I will ask you specific questions; you are not obliged to answer any of the questions if you do not feel comfortable about it. You are free to withdraw from this interview at any point without any undesirable consequences to your status in this community.

**Confidentiality:** we shall ensure confidentiality of the information that you provide; your names will not be written anywhere during this interview. All information you provide will be kept away and will only be accessed by the investigator for purposes of this study. Your decision to participate or not will not in any way jeopardise your relationships within the health system. If you have any questions please feel free to ask me

**Acceptance:** the interview will take about 45 minutes to answer all the questions. Before we proceed I would like to seek your permission to participate in this interview. Do you accept to participate in this interview for the survey?

(Circle) Yes or No

I have received a full explanation about this survey and understand its purpose and objectives. I understand the details and hereby agree to participate in the survey

Signature of respondent Date

Signature of interviewer Date

In case of any doubts please call Richard Mangwi on mobile number **0772 829 377** or John Ssempebwa on **0703 944404** the chairman of the Higher Degrees and Ethics Committee of the School of Public Health

**TBAs & Elderly care givers Runyoro translation**

1. **EKIHANDIKO EKYOSABA ORUKUSA HABWO KUSERURIRIZA HABAKAZI ABAINA ENDA ABAGENDA OMWIRO OKUBAKERERA**

**EKYEYANJUURA**

Amabara gange banyeta ………………………, Ndi omu abakurabanganamu nibecumitiriza ebye endolera eyabakazi abe enda hamu ne enkerembe omunyamasaza ya Masindi na Kiryandongo

**OMUGASO GWOKWECUMITIRIZA KUNU**

Endolera eya abakazi benda ne enkerembe eikaire eri mbi kandi ekyali mbi, mu Masindi no omu Uganda yoona. Tukimanyire nti ekizibu kinu kisobora kuhwaho kakuba tukoraginira hamu na abakyara abenda.

Tukwetaga tubasomesege obu barabaga baizire kubakebera.Nyija kumara akasumi nituhanura hamu habikukwatana ha enda yawe egi , nebindi byoona ebikwatagana na abakazi abaina enda.

Twija nokuhanura omulingo gwokwikara nobwomezi burungi obu oine enda, okwetekaniriza enkerembe yawe .Tukwija kutegeka hamu okuzara kwawe kandi nkoku owetegekera ebizibu byoona. kakusagwa Oija .Twiija kuhanuramu nkoku balolera enkerembe, obwo nezarwa ,nekyazarwa. Ekigenderwa ekikuru kiri oikale kurungi,ozale kurungi kandi no omwana abe kurungi.

**EMIGENDERE EYO OKWICUMITIRIZA KUNU**

Tutukusobora kukaguza abakazi benda boona habwokuba ebyo okukozesa bike. Tukomeremu bataito mu Kiryandongo na omu Masindi.

Iwe oli omu owa akaruru oukakomeremu.Tukwija kuhanura habikukwatanaha enda yawe ,okuzara kwawe, nendolera ye enkerembe yawe, omumaka gawe.

Nyija kwikara nikusura kumara omwezi gumu ,oija nkumpa enamba eyesimu yawe niyo nyekonyezege.

**OMUGASO OGWOKWECUMITIZA KUNU**

Okwecumitiriza kunu kukwija kutuyamba kwetegereza mulingo ki abakazi aba-ebenda bagoboora mukusomesebwa kandi mani ki agaturayongeramu habyo okulorera abakazi benda,hakuzalisibwa kwabu,hamu ne endolera eyenkerembe zabu.

**BIZIBU KI EBIKUSOBORA KURUGA OMUKWECUMITIRIZA KUNU**

Tiharoho kabi koona akakusobora kuruga habwokwetaba omukwecumitiriza kunu. Ebikaguzo binu tibiri babwokusera akabi baitu habwokuyamba abakazi benda ,hamu nenkerembe.

**EBYOKWETABA MU KWECUMITIRIZA KUNU**

Okwetweta mukwecukiriza kunu kuli kwobugabe.Tiharoho kuhambirizibwa kwona.Oboraba oyekirize nyija kukaguza ebikaguzo binu.Okwikirizibwa kugarukamu ebyokusobora rundi obyosimire.Okwikiribwa ebikaguzo ebindi kubireka rundi kwemereza orubazo .

**OKWECUMITIRIZA KUNU KWENSITA**

Ebituranya hanu bikusigara biri byensita.Busaho ondi wena arabisoma habwokuba nkubyahura hara numo. Ebyokungambira tibija kutabaijura endolera yawe hairwaro .Obu oraba oine ekikaguzo kyona oine obugabe kukikaguza.

**EBWOKWIRIZA KWETABA OMUKWECUMITIRIZA KUNU**

Okwecumitiriza kunu kusobora kumara edakika 25 .tutakandikire mbaire ninkusa orukusa nkukaguze.Okirize nkukaguze?

Ego, rundi nangwa.

Mazire kusomesebwa kandi nayetegeza ebikukwatana hakwecumitiriza kunu,omugaso gyakwo kandi nekigenderwa .

Omukono gwokukaguzibwa……………………………………………….Ebiro Byomwezi…………………………………………

Omukono gwokukaguza……………………………………………………Ebiro Byomwezi…………………………………………..

Obworaba oina okugurukya gurukya rundi oina ekindi kyona ekyokyenda kumanya terera; Mangwi Richard hasiimu enu  [**0772829377**](tel:0772829377) rundi Ssempebwa John  hasiimu enu **0703944404** Omukuru wa katebe akakulira  ebyengeso ha somero ya Public Health

## Annex 13: Consent form for prenatal women attending ANC clinic at primary care facility

**Introduction:** My Name is (........Interviewer), and I am part of the team conducting a study to assess the current situation of maternal and newborn care in Masindi and Kiryandongo districts.

**Purpose:** Maternal and newborn health has been and remains a major health problem in Masindi and Kiryandongo and indeed Uganda as a whole. We know that this problem can be solved by holding discussions with pregnant mothers. We want to provide health relevant information during your clinic days here at the health facility during all the subsequent visits that you will make to the clinic. I will spend some time with you to discuss your pregnancy and other matters related to pregnancy in general. I will also discuss with you how to keep healthy during this pregnancy and how to prepare for the coming baby. We will together discuss your birth plans and how to prepare for any emergencies that may arise during your pregnancy. I will discuss with you about caring for the newborn baby during delivery, and immediately after delivery. The aim of this intervention is to ensure that you have a safe pregnancy, safe delivery and a health new baby.

**Research procedure:** we are unable to discuss this with all pregnant women due to several other constraints and so will only take a representative sample for both Masindi and Kiryandongo districts. You have been selected to participate in this exercise. We will discuss with you issues to do with your pregnancy, delivery and caring for the newborn while at home. We will keep in touch with you up to one month after your delivery. You will give us any telephone contact that we can use to contact you during this period of time up to one month after your delivery

**Benefits of this study:** this study will help us to understand how these discussions with pregnant mothers can help us to improve pregnancy, delivery and caring for the newborn baby while at home.

**Potential harm from this study:** there is no harm that we foresee by you participating in this study. These questions are not meant to pass any judgment towards you and your pregnancy but rather to find ways of helping you to have a healthy pregnancy, safe delivery and a healthy newborn baby.

**Voluntary participation:** Participation in this study is voluntary. If you accept to take part then I will ask you specific questions; you are not obliged to answer any of the questions if you do not feel comfortable about it. You are free to withdraw from this interview at any point without any any compromise to the care that you will receive from this health facility.

**Confidentiality:** We shall ensure confidentiality of the information that you provide. All information you provide will be kept away and will only be accessed by the investigator for purposes of this study. Your decision to participate or not will not in any way jeopardise your relationships within the health system and subsequent care that you will receive. If you have any questions please feel free to ask me

**Acceptance:** the discussions will take about 25 minutes. Before we proceed I would like to seek your permission to participate in this interview. Do you accept to participate in this interview for the survey?

(Circle) Yes or No

I have received a full explanation about this survey and understand its purpose and objectives. I understand the details and hereby agree to participate in the survey

Signature of respondent Date

Signature of interviewer Date

In case of any doubts please call Richard Mangwi on mobile number 0772 829 377 or John Ssempebwa on **0703944404** the chairman of the Higher Degrees and Ethics Committee of the School of Public Health

Runyoro translation for ANC women attending ANC

1. **EKIHANDIKO EKYOSABA ORUKUSA HABWO KUSERURIRIZA HABAKAZI ABAINA ENDA ABAGENDA OMWIRO OKUBAKERERA**

**EKYEYANJUURA**

Amabara gange banyeta ………………………, Ndi omu abakurabanganamu nibecumitiriza ebye endolera eyabakazi abe enda hamu ne enkerembe omunyamasaza ya Masindi na Kiryandongo

**OMUGASO GWOKWECUMITIRIZA KUNU**

Endolera eya abakazi benda ne enkerembe eikaire eri mbi kandi ekyali mbi, mu Masindi no omu Uganda yoona. Tukimanyire nti ekizibu kinu kisobora kuhwaho kakuba tukoraginira hamu na abakyara abenda.

Tukwetaga tubasomesege obu barabaga baizire kubakebera.Nyija kumara akasumi nituhanura hamu habikukwatana ha enda yawe egi , nebindi byoona ebikwatagana na abakazi abaina enda.

Twija nokuhanura omulingo gwokwikara nobwomezi burungi obu oine enda, okwetekaniriza enkerembe yawe .Tukwija kutegeka hamu okuzara kwawe kandi nkoku owetegekera ebizibu byoona. kakusagwa Oija .Twiija kuhanuramu nkoku balolera enkerembe, obwo nezarwa ,nekyazarwa. Ekigenderwa ekikuru kiri oikale kurungi,ozale kurungi kandi no omwana abe kurungi.

**EMIGENDERE EYO OKWICUMITIRIZA KUNU**

Tutukusobora kukaguza abakazi benda boona habwokuba ebyo okukozesa bike. Tukomeremu bataito mu Kiryandongo na omu Masindi.

Iwe oli omu owa akaruru oukakomeremu.Tukwija kuhanura habikukwatanaha enda yawe ,okuzara kwawe, nendolera ye enkerembe yawe, omumaka gawe.

Nyija kwikara nikusura kumara omwezi gumu ,oija nkumpa enamba eyesimu yawe niyo nyekonyezege.

**OMUGASO OGWOKWECUMITIZA KUNU**

Okwecumitiriza kunu kukwija kutuyamba kwetegereza mulingo ki abakazi aba-ebenda bagoboora mukusomesebwa kandi mani ki agaturayongeramu habyo okulorera abakazi benda,hakuzalisibwa kwabu,hamu ne endolera eyenkerembe zabu.

**BIZIBU KI EBIKUSOBORA KURUGA OMUKWECUMITIRIZA KUNU**

Tiharoho kabi koona akakusobora kuruga habwokwetaba omukwecumitiriza kunu. Ebikaguzo binu tibiri babwokusera akabi baitu habwokuyamba abakazi benda ,hamu nenkerembe.

**EBYOKWETABA MU KWECUMITIRIZA KUNU**

Okwetweta mukwecukiriza kunu kuli kwobugabe.Tiharoho kuhambirizibwa kwona.Oboraba oyekirize nyija kukaguza ebikaguzo binu.Okwikirizibwa kugarukamu ebyokusobora rundi obyosimire.Okwikiribwa ebikaguzo ebindi kubireka rundi kwemereza orubazo .

**OKWECUMITIRIZA KUNU KWENSITA**

Ebituranya hanu bikusigara biri byensita.Busaho ondi wena arabisoma habwokuba nkubyahura hara numo. Ebyokungambira tibija kutabaijura endolera yawe hairwaro .Obu oraba oine ekikaguzo kyona oine obugabe kukikaguza.

**EBWOKWIRIZA KWETABA OMUKWECUMITIRIZA KUNU**

Okwecumitiriza kunu kusobora kumara edakika 25 .tutakandikire mbaire ninkusa orukusa nkukaguze.Okirize nkukaguze?

Ego, rundi nangwa.

Mazire kusomesebwa kandi nayetegeza ebikukwatana hakwecumitiriza kunu,omugaso gyakwo kandi nekigenderwa .

Omukono gwokukaguzibwa……………………………………………….Ebiro Byomwezi…………………………………………

Omukono gwokukaguza……………………………………………………Ebiro Byomwezi…………………………………………..

Obworaba oina okugurukya gurukya rundi oina ekindi kyona ekyokyenda kumanya terera; Mangwi Richard hasiimu enu  [**0772829377**](tel:0772829377) rundi Ssempebwa John hasiimu enu  **0703944404** Omukuru wa katebe akakulira  ebyengeso ha somero ya Public Health

| Annex 14: Budgetsi) Budget Sub study one: Assessing the primary health care workers' knowledge-base and perceptions about educational interventions during ANC clinics | | | | | | | | | | |
| --- | --- | --- | --- | --- | --- | --- | --- | --- | --- | --- |
| **Phase** | | **Activity Detail** | | **Number of Officers** | **Rate** | | **Number of days** | | **Totals** | |
| **Preparatory Phase** | | IRB costs | | 1 | 600,000 | | 1 | | 600,000 | |
|  |  | Uganda National Council of Science & Technology | | 1 | 720,000 | | 1 | | 720,000 | |
|  |  | Identify and Constitute Research Team Assistants etc | | 1 | 30,000 | | 1 | | 30,000 | |
|  |  | Per diems for Research Assistants | | 5 | 60,000 | | 10 | | 3,000,000 | |
|  |  | Per diems for Researcher | | 2 | 90,000 | | 10 | | 1,800,000 | |
|  |  | Training of Research Assistants | | 5 | 60,000 | | 3 | | 900,000 | |
|  |  | Pretesting of Study tools | | 1 | 100,000 | | 2 | | 200,000 | |
|  |  | Correction & Assembly of necessary study tools | | 200 | 300 | | 1 | | 60,000 | |
| **Field Costs** | | Local Car hire | | 2 | 80,000 | | 10 | | 1,600,000 | |
|  |  | Car Hired from Kampala | | 1 | 150,000 | | 12 | | 1,800,000 | |
|  |  | Fuel to and from Kampala | | 1 | 200,000 | | 2 | | 400,000 | |
|  |  | Fuel for Local field Travels | | 3 | 50,000 | | 10 | | 1,500,000 | |
| **Data Handling** | | Data entry & Cleaning | | 2 | 350,000 | | 7 | | 4,900,000 | |
|  |  | Data analysis | | 1 | 2,000,000 | | 1 | | 2,000,000 | |
|  |  | Manuscript writing | | 1 | 150,000 | | 1 | | 150,000 | |
| **Dissemination,**  **Manuscript writing and Publication** | | Discussions of results with District Officials | | 20 | 10,000 | | 2 | | 400,000 | |
|  |  | Discussion of results at the School of Public Health | | 1 | 100,000 | | 1 | | 100,000 | |
|  |  | Discussion of results at the MOH | | 20 | 50,000 | | 1 | | 1,000,000 | |
|  |  | Preparation of Manuscript for Publication | | 1 | 600,000 | | 2 | | 1,200,000 | |
|  | |  | |  |  | |  | | **22,360,000** | |
| Budget Sub study two: Determine prevalent prenatal and newborn homecare practices, their determinants and rationale | | | | | | | |  | |  |
| **Phase** | **Activity Detail** | | **Number of Officers** | | **Rate** | **Number of days** | | **Totals** | |  |
| **Preparatory Phase** | Uganda National Council of Science & Technology | | 1 | | 720,000 | 1 | | 720,000 | |  |
|  | IRB costs | | 1 | | 600,000 | 1 | | 600,000 | |  |
|  | Identify and Constitute Research Team Assistants etc | | 1 | | 30,000 | 1 | | 30,000 | |  |
|  | Per diems for Research Assistants | | 12 | | 60,000 | 10 | | 7,200,000 | |  |
|  | Per diems for Researchers | | 2 | | 90,000 | 10 | | 1,800,000 | |  |
|  | Training of Research Assistants | | 12 | | 60,000 | 4 | | 2,880,000 | |  |
|  | Pretesting of Study tools | | 1 | | 100,000 | 2 | | 200,000 | |  |
|  | Correction & Assembly of necessary study tools | | 200 | | 300 | 1 | | 60,000 | |  |
| **Field Costs** | Local Car hire | | 2 | | 80,000 | 10 | | 1,600,000 | |  |
|  | Car Hired from Kampala | | 1 | | 150,000 | 12 | | 1,800,000 | |  |
|  | Fuel to and from Kampala | | 1 | | 200,000 | 2 | | 400,000 | |  |
|  | Fuel for Local field Travels | | 3 | | 50,000 | 10 | | 1,500,000 | |  |
| **Data Handling** | Data entry & Cleaning | | 2 | | 350,000 | 7 | | 4,900,000 | |  |
|  | Data analysis | | 1 | | 2,000,000 | 1 | | 2,000,000 | |  |
|  | Manuscript writing | | 1 | | 150,000 | 1 | | 150,000 | |  |
| **Dissemination, Manuscript writing and Publication** | Discussions of results with District Officials | | 20 | | 10,000 | 2 | | 400,000 | |  |
|  | Discussion of results at the School of Public Health | | 1 | | 100,000 | 1 | | 100,000 | |  |
|  | Discussion of results at the MOH | | 20 | | 50,000 | 1 | | 1,000,000 | |  |
|  | Preparation of Manuscript for Publication | | 1 | | 600,000 | 2 | | 1,200,000 | |  |
| **TOTAL** |  | |  | |  |  | | **28,540,000** | |  |

| iii) Sub study three: Community trial: Intervention to enhance prenatal educational interventions by CHW at the primary healthcare level | | | | | |  |
| --- | --- | --- | --- | --- | --- | --- |
| **Phase** | **Activity Detail** | **Number of Officers** | **Rate** | **Number of days** | **Totals (Ug Shs)** | **Sub Totals** |
| **Preparatory Phase** | Uganda National Council of Science & Technology | 1 | 720,000 | 1 | 720,000 |  |
|  | Pre-visits to the district to discuss formative results with local team | 2 | 3,400,000 | 2 | 13,600,000 |  |
|  | Constitution of the local team | 2 | 250,000 | 2 | 1,000,000 |  |
|  | Presentation of study protocol to district officials | 25 | 12,000 | 2 | 600,000 |  |
|  | Discussions of terms of reference for local team | 80 | 12,000 | 4 | 3,840,000 |  |
|  | Roles and functions of team members | 80 | 12,000 | 2 | 1,920,000 |  |
|  | IRB costs | 1 | 600,000 | 1 | 600,000 |  |
|  | procurement of mobile cell phones | 36 | 100,000 | 1 | 3,600,000 |  |
|  | maintenance of mobile connectivity | 36 | 100,000 | 12 | 43,200,000 |  |
|  | Stationary costs for data files | 20 | 3,000 | 200 | 12,000,000 |  |
|  | Two-Monthly Field visits by PI | 1 | 3,500,000 | 6 | 21,000,000 |  |
|  | Correction & Assembly of necessary study tools | 200 | 300 | 1 | 60,000 | 102,140,000 |
|  |  |  |  |  |  |  |
| **Field Costs** | Local Car hire | 2 | 80,000 | 10 | 1,600,000 |  |
|  | Car Hired from Kampala | 10 | 150,000 | 5 | 7,500,000 |  |
|  | Fuel to and from Kampala | 10 | 200,000 | 2 | 4,000,000 |  |
|  | Fuel for field work | 12 | 50,000 | 5 | 3,000,000 |  |
|  | Monthly wages for CHWs | 22 | 10,000 | 12 | 2,640,000 |  |
|  | Compensation for HA supervising CHWs | 18 | 10,000 | 12 | 2,160,000 |  |
|  | Additional incentives for additional work done by Primary care facility staff | 22 | 5,000 | 12 | 1,320,000 |  |
|  | Transport refund for monthly review sessions with HA | 22 | 5,000 | 12 | 1,320,000 |  |
|  | Procurement of essential supplies and medicines at Primary care centres participating in the study | 15 | 100,000 | 12 | 18,000,000 |  |
|  | Monthly review of study progress | 20 | 12,000 | 12 | 2,880,000 |  |
|  | Fuel for Routine fort-nightly field visits by HA | 2 | 30,000 | 24 | 1,440,000 |  |
|  | Compensation of Health centre staff for additional work | 80 | 5,000 | 12 | 4,800,000 | 50,660,000 |
|  |  |  |  |  |  |  |
| **Assessment of Intervention** | Identify and Constitute Research Team Assistants etc | 1 | 30,000 | 1 | 30,000 |  |
|  | Per diems for Research Assistants | 12 | 60,000 | 10 | 7,200,000 |  |
|  | Per diems for Researchers | 2 | 90,000 | 10 | 1,800,000 |  |
|  | Training of Research Assistants | 12 | 60,000 | 4 | 2,880,000 |  |
|  | stationary for data collection and tools | 2 | 500,000 | 2 | 2,000,000 |  |
|  | Pretesting of Study tools | 1 | 100,000 | 2 | 200,000 | 14,110,000 |
|  |  |  |  |  |  |  |
| **Data Handling** | Data entry & Cleaning | 2 | 350,000 | 17 | 11,900,000 |  |
|  | Data analysis | 1 | 5,000,000 | 2 | 10,000,000 |  |
|  | Manuscript writing | 1 | 150,000 | 1 | 150,000 |  |
| **Dissemination and Manuscript writing and Publication** | Discussions of results with District Officials | 20 | 10,000 | 2 | 400,000 |  |
|  | Discussion of results at the School of Public Health | 1 | 100,000 | 1 | 100,000 |  |
|  | Discussion of results at the MOH | 20 | 50,000 | 1 | 1,000,000 |  |
|  | Preparation of Manuscript for Publication | 1 | 600,000 | 2 | 1,200,000 | 24,750,000 |
|  |  |  |  |  |  |  |
| **TOTAL** |  |  |  |  | **191,660,000** | **191,660,000** |

# Annex 15: SOPs: Standard Operating Procedure and intervention packages

### i) Standard operating procedure and intervention package for educational intervention during ANC

**(**[**Bhutta Z, Sajid S et al. 2011**](#_ENREF_10)**)**

When the pregnant women enter the clinic room

1. Identify pregnant women with all of the following conditions:
2. First visit of the current pregnancy,
3. Not more than 28 weeks of gestation
4. Administer the consent form
5. If she consents then open a file for her
6. Request for her ANC card
7. Enter all personal details available on the ANC card in to the file
8. Record any telephone contacts that you can use to access her in future

name, age, ethnicity, parity, LNMP, EDD, gestation age of the first visit, history of previous pregnancy outcome of the previous pregnancy (baby – alive or dead, mode of delivery), distance from the health facility, marital status, history of previous ANC attendance-Number of attendance, delivery (home, health facility, TBA etc).

Note the date for the next appointment, (all this information will be used to tailor the discussions)

-if she consistently attended all the expected milestones-then you find out what her motivation was to do so, and whether she plans to do the same for the current pregnancy;

- If not consistent-discuss the reasons for inconsistencies and help her to find a solution,

- diet during pregnancy-find out what foods are readily consumed and what she can easily afford, what foods could be prohibited during pregnancy (caution about antagonising cultures-you can find another acceptable alternative source of the food value) and discuss the importance for balances in diet, fruits, green vegetables, proteins and carbohydrates,

- Discuss work related activities-how much she does and who helps her with the routine household and garden chores.

- Discuss importance of ANC even when she has been attending consistently, the advantages of delivering in the health facility even when she has been delivering at the health facility,

- Birth preparation-clean clothes for the newborn (emphasis that clean does not mean new!-even old rages can be patched together washed clean, clean environment for the newborn,

- caring for the cord, dangers of applying substances to the cord,

- Pre-lacteal feeds-and its disadvantages-explain how breast milk eventually comes and the usefulness of colostrum.

- Discuss bathing of the new baby how soon and how to delay (delay up to 24 hours),

- Immunisation of the child, polio 0, BCG etc.

- Recognition of a sick child-hot to touch, refusing to feed, crying unusually,

All these information should be derived from discussions with the pregnant woman-not as a set of instructions to be strictly followed-compromises should be made at every stage-all that is agreed upon should be detailed down in the file. The counsellor should moderate the discussions, if the pregnant woman has come with a partner or a caretaker; both of them should be involved in the discussions even if the care taker is not pregnant-if there is a caretaker present then this should be recorded and state the relationship between them.

Tick the areas that were discussed, mark with a cross what you did not discuss with her, note down important decisions that you have agreed upon with the participant-you will use this as a reference point in subsequent meetings and discussions

Ensure that the discussion does not exceed 30 minutes unless there is expressed interest from the participant to continue. And this expression should be noted down in the file.

For subsequent visits indicate appointment dates and actual revisit dates

Later on follow-up for delivery and record where she has delivered from

Assess for the different practices outlined annex 6 will be used to assess the following outcomes

Place of delivery, Cutting of the cord, care for the cord, initiation of breast feeding, provision of pre-lacteal feeds, in case of illness how it was recognised, what action was taken, how soon was the care seeking from onset of disease or when the decision was made that the child was unwell, what facility did she take the child, outcome, at the periphery of assessment the researcher will want to know whether the child is still alive just at one month.

### ii) Standard Operating procedure for the use of mobile cell phones

1. Only voice messages will be deployed in this intervention
2. Phones will be programmed to connect between CHWs, HAs, Health workers on call at the primary care facility and the reference hospital
3. Only the health centre staff cell phones will directly connect to the hospital in case of emergency evacuation of a pregnant woman or newborn baby
4. Community Health workers living in the community will own the mobile phones
5. CHWs will dial the midwife or health staff on duty to make any enquiries regarding pregnancy or newborn care as and when the need may arise;
6. CHWs will receive health specific advice from the technical staff based at the health facility-(a specific health staff will be scheduled on-call in order to respond to such enquires from the CHWs)
7. Midwifes will dial CHWs in case of any specific prenatal woman who may need a closer follow-up for her pre-existing medical condition or who requires a closer follow-up given specific complication with the current pregnancy
8. Midwife will also call CHWs to make reminders for ANC return visits and due dates for delivery for pregnant women
9. Midwives or health worker on-call will dial the emergency hospital number in case of evacuation of prenatal or newborn baby or both

### iii) Standard Operating Procedure for Health Assistants based at the health facility

1. Health assistant will draw a timetable for all three CHWs who will provide regular prenatal education interventions at the health facility
2. He/she will make routine fortnightly field visits to the CHWs providing services at the primary care facility
3. He/she will organise monthly face-to-face sessions with CHWs providing educational interventions at the health facility
4. He/she will ensure that the forth ANC first attendant is appropriately sampled
5. Ensures that all details regarding the prenatal woman is accurately entered and archived by the CHWs

Ensures recordings of the monthly sessions with CHWs are recorded and archived to be shared with the PI during the bi-monthly field visits

**Annex 15:** HOUSEHOLD EMPOWERMENT-‘ENABLEMENT’

The Patient-Enabling-Initiative PEI tool, adapted from ([Howie J, Heaney D et al. 1998](#_ENREF_42)), will be used to assess secondary outcome measures ([Lewin S, Munabi-Babigumira S et al. 2010](#_ENREF_54)): This tool will be administered to those women who have attended the intervention arm

Household Empowerment tool

|  | **As a result of your interaction with the VHT did you feel you** | **Much better** | **better** | **Same or less** |
| --- | --- | --- | --- | --- |
| 1 | Are able to **cope** with your routines during pregnancy, delivery and caring for newborn care? |  |  |  |
| 2 | Are able to **understand** what is going on during pregnancy, labour and your newborn during the next pregnancy? |  |  |  |
| 3 | Were you able to **cope** with the pregnancy, delivery and care for the newborn? |  |  |  |
| 4 | Were **able** to keep yourself and the newborn healthy? |  |  |  |
|  |  | **Much more** | **more** | **Same or less** |
| 5 | Were **confident** about your health and the health of the newborn? |  |  |  |
| 6 | Were able to take care of yourself and the newborn? |  |  |  |
